# X-ray Diffraction Analysis and Williamson-Hall Method in USDM Model for Estimating More Accurate Values of Stress-Strain of Unit Cell and Super Cells (2 × 2 × 2) of Hydroxyapatite, Confirmed by Ultrasonic Pulse-Echo Test

**DOI:** 10.3390/ma14112949

**Published:** 2021-05-30

**Authors:** Marzieh Rabiei, Arvydas Palevicius, Amir Dashti, Sohrab Nasiri, Ahmad Monshi, Akram Doustmohammadi, Andrius Vilkauskas, Giedrius Janusas

**Affiliations:** 1 Faculty of Mechanical Engineering and Design, Kaunas University of Technology, LT-51424 Kaunas, Lithuania; sohrab.nasiri@ktu.edu (S.N.); andrius.vilkauskas@ktu.lt (A.V.); 2 Department of Materials Science and Engineering, Sharif University of Technology, Tehran 11365-9466, Iran; a.dashty@merc.ac.ir; 3 Department of Materials Engineering, Isfahan University of Technology, Isfahan 84154, Iran; dr.ahmad.monshi@gmail.com; 4 Materials and Energy Research Center (MERC), Meshkin-Dasht, Karaj 31787-316, Iran; hivadoostmohammadi91@gmail.com

**Keywords:** Williamson-Hall (W-H), uniform stress deformation model (USDM), Young’s modulus, X-ray diffraction, hydroxyapatite, planar density, ultrasonic pulse-echo

## Abstract

Taking into account X-ray diffraction, one of the well-known methods for calculating the stress-strain of crystals is Williamson-Hall (W–H). The W-H method has three models, namely (1) Uniform deformation model (UDM); (2) Uniform stress deformation model (USDM); and (3) Uniform deformation energy density model (UDEDM). The USDM and UDEDM models are directly related to the modulus of elasticity (E). Young’s modulus is a key parameter in engineering design and materials development. Young’s modulus is considered in USDM and UDEDM models, but in all previous studies, researchers used the average values of Young’s modulus or they calculated Young’s modulus only for a sharp peak of an XRD pattern or they extracted Young’s modulus from the literature. Therefore, these values are not representative of all peaks derived from X-ray diffraction; as a result, these values are not estimated with high accuracy. Nevertheless, in the current study, the W-H method is used considering the all diffracted planes of the unit cell and super cells (2 × 2 × 2) of Hydroxyapatite (HA), and a new method with the high accuracy of the W-H method in the USDM model is presented to calculate stress (σ) and strain (ε). The accounting for the planar density of atoms is the novelty of this work. Furthermore, the ultrasonic pulse-echo test is performed for the validation of the novelty assumptions.

## 1. Introduction

Young’s modulus (E) plays an important role in the characterization of mechanical properties, and accurate knowledge of the engineering values of elastic modulus is vital for understanding materials’ stiffness [1]. Recently, ceramic materials have been favored in industrial applications, because they exhibit good mechanical properties, such as high elastic moduli and high hardness [2]. One of the well-known bio ceramics is hydroxyapatite, which has bioactive properties very close to natural bone mineral and it has special biological significance [3]. The chemical formula of hydroxyapatite is Ca_10_(PO_4_)_6_(OH)_2_ and it differs very little from bone tissue [4,5]. Understanding the mechanical properties of hydroxyapatite during the crystallization and growth stages of the synthesis processes is important because the Young’s modulus affects the growth of hydroxyapatite crystal in mechanically strained environments directly [6]. Therefore, paying attention to the mechanical properties and structural geometry of hydroxyapatite can be helpful for research and industrial applications. Paying attention to the details of the structural geometry of hydroxyapatite is essential for employing an easy, cost-effective and reliable method to determine the Young’s modulus. In this research, we have developed a method based on the linear regression of the Young’s modulus of each plane of the crystal lattice versus planar density to obtain a reliable total Young’s modulus of materials. Hence, in this study, for the first time, we calculated the exact planar density derived from the diffracted planes of hydroxyapatite in unit cells and super cells (2 × 2 × 2). Then, we determined and investigated the total Young’s modulus of samples. Furthermore, to determine the effect of cell size on the Young’s modulus, an extensive, exact calculation of the planar density of super cells (2 × 2 × 2) of hydroxyapatite, and comparing it with the result obtained from unit cell calculations, was performed. The Williamson-Hall (W-H) method is a procedure to analyse stress and strain derived from X-ray diffraction. Ultrasonic pulse-echo is a scan representation commonly used for thickness measurement and sizing of the defect in an ultrasonic test in which the signal is reflected from a discontinuity in a test of material structure. This test is performed in this study for the confirmation of the validity of the novelty assumptions. The configuration of the ultrasonic pulse echo is called the acoustic sound energy and localizes the discontinuities or defect indication. In addition, ultrasonic pulse echo measurements can be used to determine the elastic constant and elastic compliance of compounds [7]. According to the W-H method, the basic calculation for the plot can be performed by using the XRD data. The big problem with utilizing the W-H method in the USDM model is attributed to the values of Young’s modulus in the equation. Because in all studies and research, the values of Young’s modulus have been reported for one sharp peak of an XRD pattern or the average values of Young’s modulus; both of them have an error because the values are not representative of whole diffracted planes. For example, Ratan et al. calculated the stress (σ) and strain (ε) of cadmium selenide (CdSe) nanoparticles and they considered the average value of Young’s modulus from the Williamson-Hall (W-H) method in the USDM model [8]. Furthermore, Khorsand et al. reported the Young’s modulus value of ZnO nanoparticles, considering a sharp peak of X-ray diffraction of ZnO [9]. In another study, Rabiei et al. presented the USDM model of the Young’s modulus value for hydroxyapatite and the Young’s modulus value was considered an average of diffracted planes [5]. Moreover, Madhavi et al. utilized the USDM model of the Young’s modulus value for VO_2_ doped ZnS/CdS composite nanopowder and they calculated the Young’s modulus value derived from average data. Rameshbabu et al. submitted the W-H method in the USDM model for calculating stress and strain values of hydroxyapatite and they also utilized the average value of Young’s modulus [10]. In all research that has utilized the W-H method in the USDM model, the Young’s modulus values of the investigated materials are reported as an average value or selected through the literature, and reported values are not inclusive of high accuracy. Interestingly, the values of the elastic compliance constant of studied materials were also derived from the literature, whereas each material has a special elastic compliances constant related to itself; therefore, with reference to the literature and utilizing the compliance constant values of studies, the accuracy of the report would be decreased. In addition, the W-H method is well suited to calculating and estimating the stress and strain of materials [10]. By applying our method and deploying the results of the W-H method, the stress-strain of a sample can be calculated with high accuracy. We have used both DFT calculations and ultrasonic measurements to compare and evaluate the validity of the proposed results (details are in the Supplementary materials). Overall, the evaluated results and the extracted values of this study were in good agreement with the theoretical, experimental and literature values.

## 2. Methods

The synthesis route of hydroxyapatite powder is explained completely in part 2 of the Support Information (preparation of hydroxyapatite powder).

### 2.1. Structural Analyses of Synthesized Hydroxyapatite

**Figure 1:**
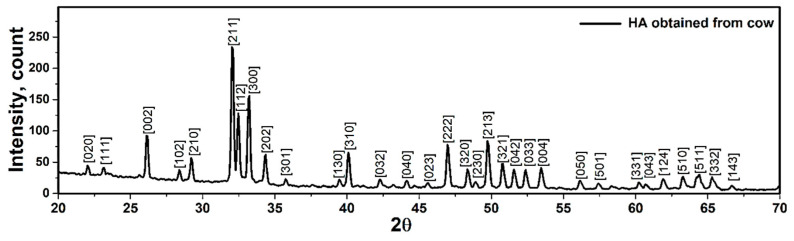
X-ray diffraction pattern of hydroxyapatite synthesized at 950 °C.

The XRD pattern of synthesized hydroxyapatite powder is shown in Figure 1. The XRD pattern exhibits several diffraction peaks, which can be indexed as the hexagonal hydroxyapatite. The XRD pattern was evaluated based on X’pert and the pattern was in agreement with the standard XRD peaks of hydroxyapatite (ICDD 9-432). Similar observations were reported in References [11,12]. In addition, crystallographic parameters and details of each diffracted plane of hydroxyapatite were evaluated by X’pert and the values are tabulated in Table 1 and Table S1.

According to Table S1, the values of the distance between planes are calculated by Equation (1). In this equation, h, k and l are indices of each plane, and a, c and d are lattice parameters and distance of planes, respectively [13].

(1) $$\frac{1}{d^{2}}=\frac{4}{3}\;\left(\frac{h^{2}+\mathrm{hk}+k^{2}}{a^{2}}\right)+\frac{l^{2}}{c^{2}}$$

**Figure 2:**
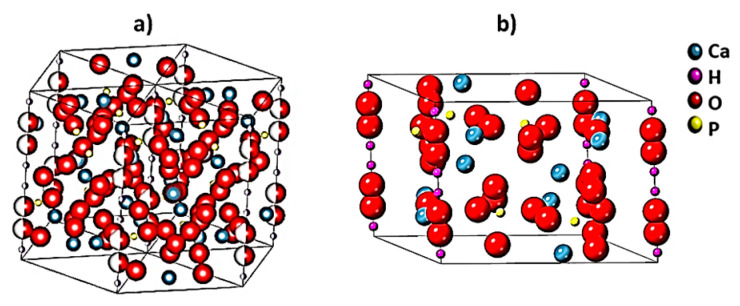
Schematic representation of (**a**) hydroxyapatite unit cell and (**b**) the hydroxyapatite structure extracted by cif file.

Hydroxyapatite has a hexagonal system with a P6_3_/m space group and has little deviation from stoichiometry [14]. Figure 2 shows a sketch of a unit cell of hexagonal hydroxyapatite and a cif file of synthesized hydroxyapatite. There are two different situations of calcium ions and, in total, 18 ions are closely packed to create the hexagonal structure. At each hexagonal corner, a calcium ion is restricted by 12 calcium ions shared with 3 hexagons. Void spaces between two hexagons are filled with three phosphate tetrahedral per unit cell. Ions in hydroxyapatite can be interchangeably replaced with biologically useful ions due to the inherent versatility of this crystal structure and can also be referred to as doping. In addition, the substitution of calcium, phosphate and/or hydroxyl ions is possible [15]. Notably, the specific feature of hydroxyapatite is related to the ${\mathrm{OH}}^{-}$ ions forming inner channels along the c axis. This property plays an important role in its mechanical and physical properties [16]. In addition, the Edax analysis of synthesized hydroxyapatite is presented in Figure S2. According to the EDX analysis, the value of the Ca/P ratio for hydroxyapatite obtained from bovine bone was recorded to be 1.60. In addition, thermal decomposition of hydroxyapatite into tricalcium phosphate and tetra calcium phosphate was not observed during the sintering, as in References [17,18], so the hydroxyapatite was successfully synthesized.

### 2.2. Planar Density of Unit Cell and Super Cells (2 × 2 × 2) of Hydroxyapatite

**Figure 3:**
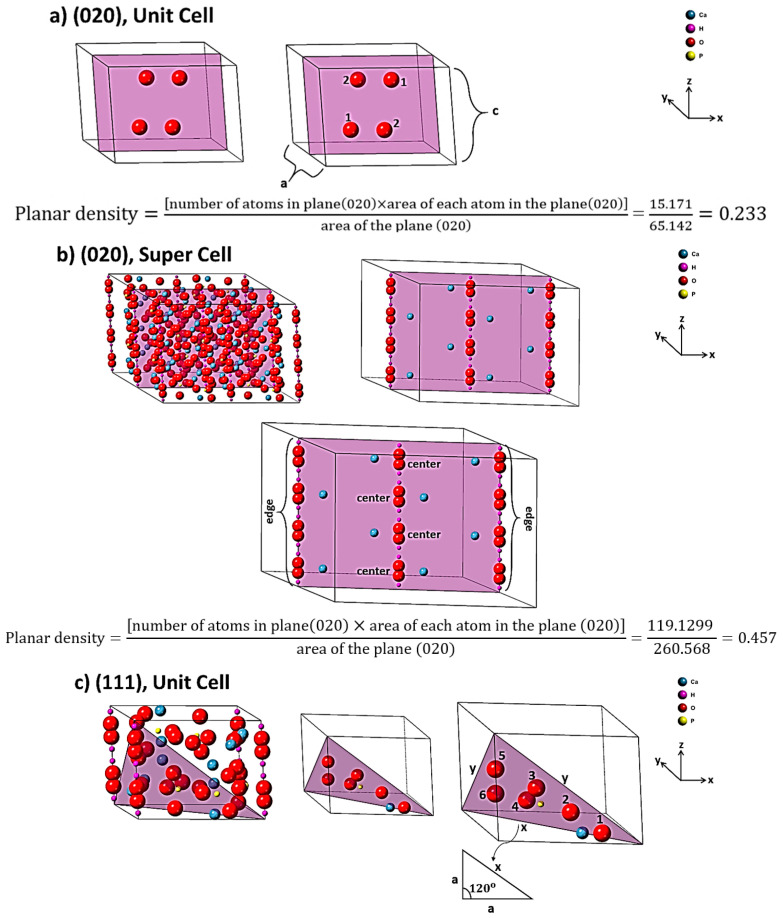

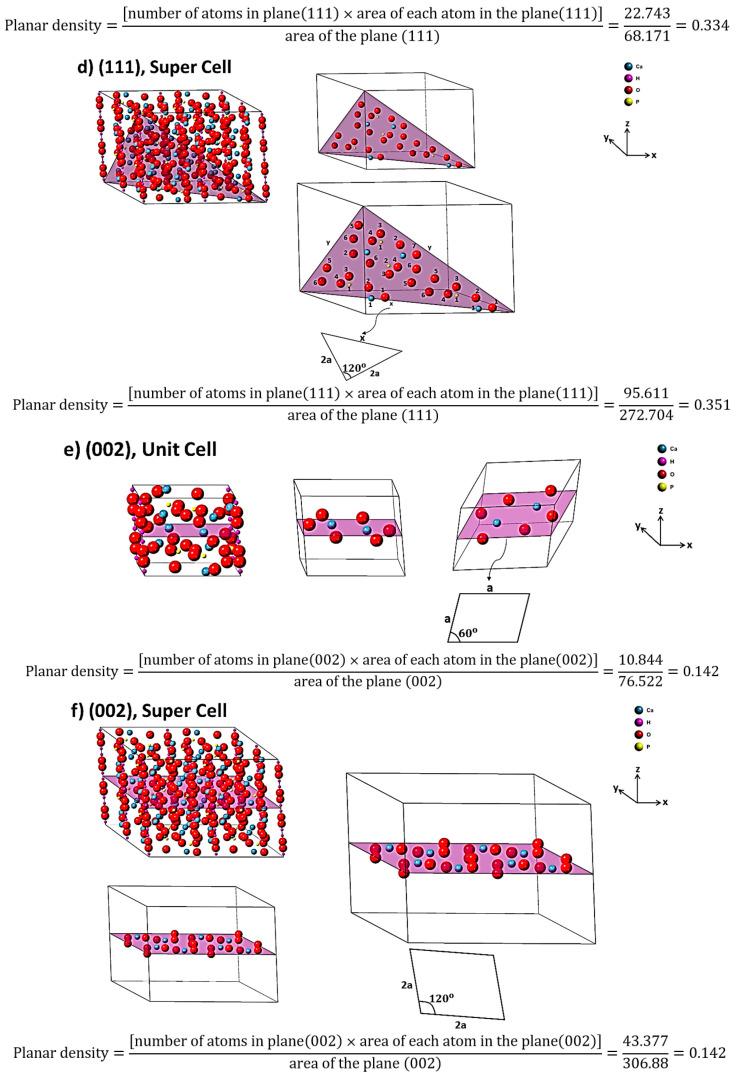

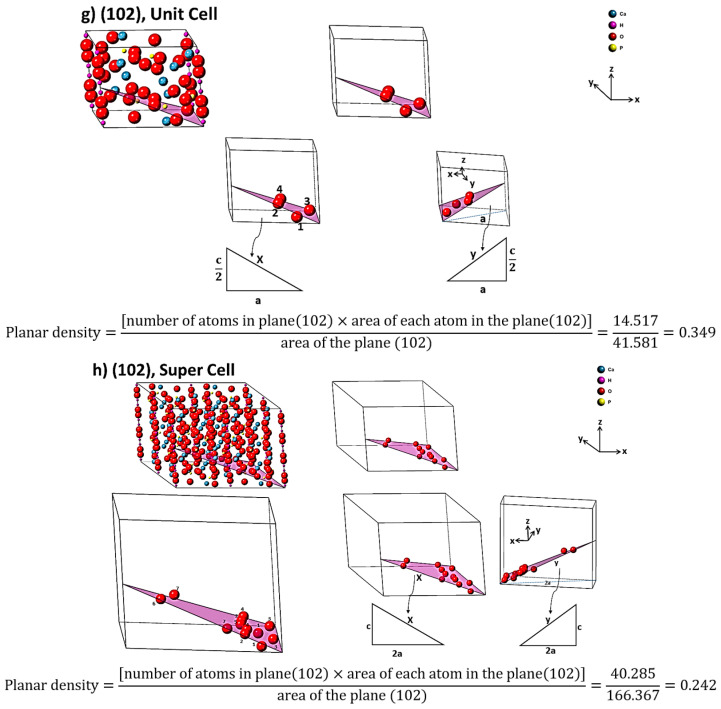
Array and position of the involved atoms such as (**a**) (020) unit cell, (**b**) (020) super cell, (**c**) (111) unit cell, (**d**) (111) super cell, (**e**) (002) unit cell, (**f**) (002) super cell, (**g**) (102) unit cell and (**h**) (102) super cell (the calculations are in the Supplementary materials).

According to the resulting list of planes by X-ray diffraction (Figure 1, Table S1), the planar density values of each diffracted plane in unit cell and super cells (2 × 2 × 2) of hydroxyapatite are calculated in Figure 3, Figures S6 and S7. To calculate the planar density values, diffracted planes were selected from a low angle to a high angle in tandem. It is worth mentioning that the matrix of all super cell lattices was considered to be 2 × 2 × 2. According to the center of atoms, the planar density is calculated by the area of the atoms in the plane divided by the total area of that plane [19].

### 2.3. Elastic Stiffness Constant and Elastic Compliance of Hydroxyapatite

Hooke’s law is shown in Equation (2); the stress corresponds to the strain for small displacements. It is the basic form, that this symmetry can be converted to the six items of stress (σ) and strain (ε) [20].

(2) $$\left(\begin{array}{c} \begin{array}{c} \sigma_{\mathrm{xx}}\\ \sigma_{\mathrm{yy}} \end{array}\\ \sigma_{\mathrm{zz}}\\ \sigma_{\mathrm{yz}}\\ \sigma_{\mathrm{zx}}\\ \sigma_{\mathrm{xy}} \end{array}\right)=\left(\begin{array}{c} \begin{array}{c} c_{11}\\ c_{21} \end{array}\\ c_{31}\\ c_{41}\\ c_{51}\\ c_{61} \end{array}\begin{array}{c} \begin{array}{c} c_{12}\\ c_{22} \end{array}\\ c_{32}\\ c_{42}\\ c_{52}\\ c_{62} \end{array}\begin{array}{c} \begin{array}{c} c_{13}\\ c_{23} \end{array}\\ c_{33}\\ c_{43}\\ c_{53}\\ c_{63} \end{array}\begin{array}{c} \begin{array}{c} c_{14}\\ c_{24} \end{array}\\ c_{34}\\ c_{44}\\ c_{54}\\ c_{64} \end{array}\begin{array}{c} \begin{array}{c} c_{15}\\ c_{25} \end{array}\\ c_{35}\\ c_{45}\\ c_{55}\\ c_{65} \end{array}\begin{array}{c} \begin{array}{c} c_{16}\\ c_{26} \end{array}\\ c_{36}\\ c_{43}\\ c_{56}\\ c_{66} \end{array}\right)=\left(\begin{array}{c} \begin{array}{c} \varepsilon_{\mathrm{xx}}\\ \varepsilon_{\mathrm{yy}} \end{array}\\ \varepsilon_{\mathrm{zz}}\\ \varepsilon_{\mathrm{yz}}\\ \varepsilon_{\mathrm{zx}}\\ \varepsilon_{\mathrm{xy}} \end{array}\right)$$

Additionally, Hooke’s law can be written (Equation (3)):

(3) $$\sigma_{\mathrm{xx}}=C_{11}\varepsilon_{\mathrm{xx}}+C_{12}\varepsilon_{\mathrm{yy}}+C_{13}\varepsilon_{\mathrm{zz}}+C_{14}\varepsilon_{\mathrm{yz}}+C_{15}\varepsilon_{\mathrm{zx}}+C_{16}\varepsilon_{\mathrm{xy}}\;\sigma_{\mathrm{yy}}=C_{21}\varepsilon_{\mathrm{xx}}+C_{22}\varepsilon_{\mathrm{yy}}+C_{23}\varepsilon_{\mathrm{zz}}+C_{24}\varepsilon_{\mathrm{yz}}+C_{25}\varepsilon_{\mathrm{zx}}+C_{26}\varepsilon_{\mathrm{xy}}\;\sigma_{\mathrm{zz}}=C_{31}\varepsilon_{\mathrm{xx}}+C_{32}\varepsilon_{\mathrm{yy}}+C_{33}\varepsilon_{\mathrm{zz}}+C_{34}\varepsilon_{\mathrm{yz}}+C_{35}\varepsilon_{\mathrm{zx}}+C_{36}\varepsilon_{\mathrm{xy}}\;\sigma_{\mathrm{yz}}=C_{41}\varepsilon_{\mathrm{xx}}+C_{42}\varepsilon_{\mathrm{yy}}+C_{43}\varepsilon_{\mathrm{zz}}+C_{44}\varepsilon_{\mathrm{yz}}+C_{45}\varepsilon_{\mathrm{zx}}+C_{46}\varepsilon_{\mathrm{xy}}\;\sigma_{\mathrm{zx}}=C_{51}\varepsilon_{\mathrm{xx}}+C_{52}\varepsilon_{\mathrm{yy}}+C_{53}\varepsilon_{\mathrm{zz}}+C_{54}\varepsilon_{\mathrm{yz}}+C_{55}\varepsilon_{\mathrm{zx}}+C_{56}\varepsilon_{\mathrm{xy}}\;\sigma_{\mathrm{xy}}=C_{61}\varepsilon_{\mathrm{xx}}+C_{62}\varepsilon_{\mathrm{yy}}+C_{63}\varepsilon_{\mathrm{zz}}+C_{64}\varepsilon_{\mathrm{yz}}+C_{65}\varepsilon_{\mathrm{zx}}+C_{66}\varepsilon_{\mathrm{xy}}.$$

The elastic stiffness determines the response of crystal to an externally applied stress or strain and provides information about bonding characteristics and mechanical and structural stability [21]. The hydroxyapatite system has five elastic constants (Equation (4)). Therefore, the values of five independent C_ij_, can be named C_11_, C_12_, C_13_, C_33_, C_44_.

(4) $$\mathrm{Hydroxyapatite}\;\mathrm{matrix}\left|\begin{array}{cccccc} C_{11} & C_{12} & C_{13} & 0 & 0 & 0\\ C_{12} & C_{11} & C_{13} & 0 & 0 & 0\\ C_{13} & C_{13} & C_{33} & 0 & 0 & 0\\ 0 & 0 & 0 & C_{44} & 0 & 0\\ 0 & 0 & 0 & 0 & C_{44} & 0\\ 0 & 0 & 0 & 0 & 0 & \frac{1}{2}\left(C_{11}-C_{12}\right) \end{array}\right|$$

The crystallographic nature of the hexagonal structure is shown in Figure S3. Furthermore, for conventional hexagonal systems, such as hydroxyapatite, the relationship between $C_{\mathrm{ij}}$ and $S_{\mathrm{ij}}$ is introduced in Equations (5)–(9) [22,23].

(5) $$S_{11}=\frac{1}{2}\;\left(\frac{C_{33}}{C_{33}\left(C_{11}+C_{12}\right)-2{\left(C_{13}\right)}^{2}}+\frac{1}{C_{11}-C_{12}}\right)$$

(6) $$S_{12}=\frac{1}{2}\;\left(\frac{C_{33}}{C_{33}\left(C_{11}+C_{12}\right)-2{\left(C_{13}\right)}^{2}}-\frac{1}{C_{11}-C_{12}}\right)$$

(7) $$S_{33}=\frac{C_{11}+C_{12}}{C_{33}\left(C_{11}+C_{12}\right)-2{\left(C_{13}\right)}^{2}}$$

(8) $$S_{13}=-\frac{C_{13}}{C_{33}\left(C_{11}+C_{12}\right)-2{\left(C_{13}\right)}^{2}}$$

(9) $$S_{44}=\frac{1}{C_{44}}\;$$

According to the Equations (5)–(9), to obtain S values C values are needed. We have used two approaches to obtain C values: First, theoretical calculations were performed via the CASTEP model of materials studio software version 6.0 in the GGA level of theory with a PBE basis set (Figure S4). The second approach is based on ultrasonic measurements (Table S2). The complete set of five elastic stiffness constant values (C_11_, C_12_, C_13_, C_33_ and C_44_) of the samples was found from ultrasonic measurements of the phase velocity anisotropy. The stiffness constant values were recorded by utilizing Equations (10)–(15). In these equations, ρ and V are density of sample and velocity, respectively [24,25,26,27,28].

(10) C11=ρV112, C22=ρV222

(11) C66=ρV122=ρV212, C55=ρV132=ρV312

(12) C12=(C11+C66−2ρV12122)(C22+C66−2ρV12122)− C66 

(13) C44=ρV232=ρV322

(14) C13=(C11+C55−2ρV13132)(C33+C55−2ρV13132)−C55

(15) C33=ρV332

For measuring the velocities, the standard ultrasonic pulse-echo ASTM E797/E797-M-15 was accomplished according to Reference [26]. In this study, the immersion procedure via water between probe and sample was utilized and the effect of pressure derived from a hand placed into the probe was decreased. In addition, the longitudinal frequency probe was 5.4 MHz, and to decrease the extension and depreciation of waves, especially the more energetic waves, a lens with a specific curvature was utilized according to the standard of Reference [28]. To measure the value of C66, a high amplitude of curve was carried out; therefore, changing the rotation angles was useful. To create the transverse waves, a probe of 2.3 MHz and a high viscos interface material (honey) were used and finally the pressure on the sample was adjusted by obtaining the better and smoother curve [29]. Taking into account each point of the samples, a three-dimensional axis, such as X_1_, X_2_ and X_3,_ can be performed. In this case, according to Figure S5, X_1_ is the radial coordinate, X_2_ is the circumferential coordinate and X_3_ is the axial coordinate. $V_{i/j}$ is the denoted velocity of an ultrasound wave that can be propagated in the X_i_ direction with particle displacements in the Xj direction simultaneously. $V_{i/j}$, with the same i and j, is longitudinal and with i ≠ j being transverse waves. For measuring quasi-longitudinal or quasi-transverse velocity (Vij/ij), specimens should be cut (bezel) on the edges toward surfaces of perpendicular X directions (Figure S5). The obtained values of velocities are shown in Table S2. On the other hand, $C_{11}$ is in good agreement with the longitudinal distortion and longitudinal compression/tension; thus, $C_{11}$ can be introduced as the hardness. Moreover, the transverse distortion depends on the $C_{12}$, and $C_{12}$ comes from the transverse expansion related to the Poisson’s ratio. Additionally, $C_{44}$ corresponds to the shear modulus, and $C_{44}$ is in the settlement with $C_{11}$ and $C_{12}$ [22,30]. Accordingly, the shear modulus is proportional to the Burgers vector and the Young’s modulus; in addition, dislocation density is in agreement with the Young’s modulus [31,32].

In the ultrasonic method, longitudinal and transverse waves were utilized for measuring the Young’s modulus value [33,34]. According to this method (Equation (16)), based on the velocity of ultrasound waves and density of sample, the Young’s modulus value was determined.

(16) $$E=\frac{{\rho c}_{l}^{2}[3\left(\frac{c_{l}}{c_{t}}{)}^{2}-4\right]}{{\left(\frac{c_{l}}{c_{t}}\right)}^{2}-1}$$

In Equation (16), ρ, $c_{l}$ and $c_{t}$ are density, velocity of longitudinal and transverse ultrasound waves tandemly. Furthermore, according to Equation (17), the velocity of longitudinal and transverse waves can be registered by determining the length of specimen and the differences between two echoes (t = t_2_ − t_1_) in the signals [35].

(17) $$c=\frac{2L}{t}$$

Here, L is the length of the sample and t is the difference between two echoes, and the density of the sample can be detected by measuring the mass and volume of the sample [36]. Additionally, with the substitution of Equations (16) and (17), the main equation for the calculation of Young’s modulus is Equation (18).

(18) $$E=\frac{4\rho {\left(\frac{L}{t_{s}}\right)}^{2}\left(3t_{s}^{2}-4t_{l}^{2}\right)}{t_{s}^{2}-t_{l}^{2}}$$

$t_{s}$ and $t_{l}$ are differences between two echoes in longitudinal and transverse waves separately [37]. The results of the theoretical calculation, and the experimental measurements of elastic stiffness constant values of hydroxyapatite (from the literature and this study), are shown in Table 2. In addition, taking into account Equations (10)–(15), the elastic compliance values of this study were calculated and are recorded in Table 3. As a result, the theoretical values were in good agreement with the theoretical values extracted by References [38,39]. The replicated values (five times) for measuring the time of transverse and longitudinal waves of the samples are presented in Table 4.

### 2.4. The Young’s Modulus versus Planar Density of Unit Cell and Super Cells (2 × 2 × 2) of Hydroxyapatite

X-ray diffraction has provided data on diffracted planes and the location of atoms in each plane. In the previous section, the planar density of each diffracted plane was calculated and it could play an important role in the mechanical properties of each plane. The Young’s modulus of each plane (E_hkl_) of a hydroxyapatite lattice can be calculated as Equation (19) [5,43]. In this equation, h, k and l are the plane indices, a and c are the lattice parameters, C and S are the elastic stiffness constant and elastic compliance, respectively. The values of Young’s modulus of 32 diffracted planes of hydroxyapatite in unit cells and super cells (2 × 2 × 2),$E_{\left(h_{1}k_{1}l_{1}\right)},\;E_{\left(h_{2}k_{2}l_{2}\right)},\;E_{\left(h_{3}k_{3}l_{3}\right)\;}\ldots \ldots \ldots \ldots .,\;E_{\left(h_{32}k_{32}l_{32}\right),}$ related to the literature and the present study, are reported in Table S3.

(19) $$E_{\mathrm{hkl}}=\frac{{\left[h^{2}+\frac{{\left(h+2k\right)}^{2}}{3}+{\left(\frac{\mathrm{al}}{c}\right)}^{2}\right]}^{2}}{S_{11}{\left(h^{2}+\frac{{\left(h+2k\right)}^{2}}{3}\right)}^{2}+S_{33}{\left(\frac{\mathrm{al}}{c}\right)}^{4}+\left(2S_{13}+S_{44}\right)\left(h^{2}+\frac{{\left(h+2k\right)}^{2}}{3}\right){\left(\frac{\mathrm{al}}{c}\right)}^{2}}$$

**Figure 4:**
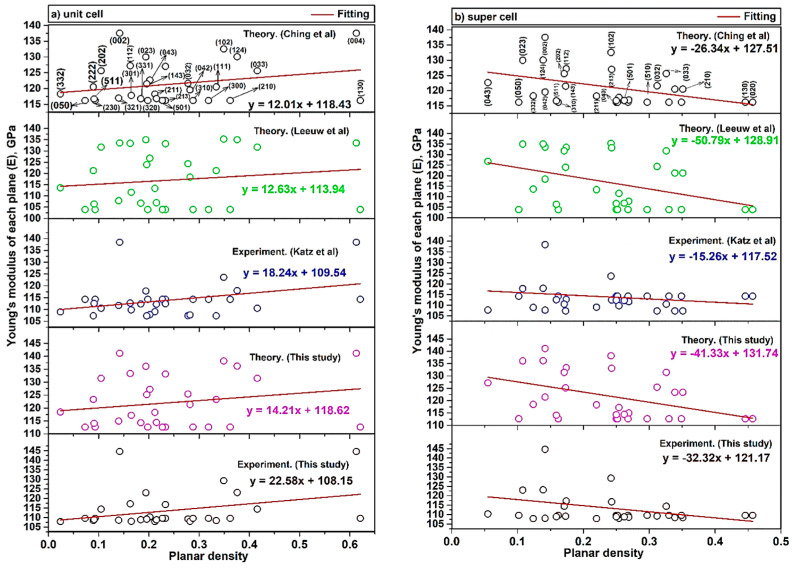
Young’s modulus of each plane (**a**) unit-cell (**b**) super cells (2 × 2 × 2) of hydroxyapatite extracted by XRD patterns and planar density.

By using the least squares method between the Young’s modulus and the planar density of diffracted planes (based on our proposed method), the Young’s modulus value of hydroxyapatite was determined with high precision. Consequently, the calculation of C and S parameters for crystalline materials is essential for the application of this method. To show the feasibility and accuracy of our proposed method for determining the Young’s modulus, the values of Young’s modulus of each plane versus the planar density of the unit cell and supercells (2 × 2 × 2) are depicted in Figure 4. The Young’s modulus values (intercept) of the unit cell and super cells (2 × 2 × 2) of hydroxyapatite, obtained from the least squares method, are tabulated in Table 5.

According to the uncertainty measurement (the measurements were replicated five times (Table 4)) and Equation (18), the Young’s modulus value gained 113.08 ± 0.14 GPa by ultrasonic measurement. This value is in good agreement with the reported values of our study in Table 5. In this study, the difference between theory and experiment values for both unit cell and super cells (2 × 2 × 2) are identical. This difference for unit cell and super cells (2 × 2 × 2) are 10.47 GPa and 10.57 GPa, respectively. This means that the theoretical calculation is valid and, by reducing it by about ~10 GPa, the experimental values can be obtained.

## 3. Result and Discussion

### 3.1. Positive and Negative Slope Values of Unit Cell and Super Cells (2 × 2 × 2)

The difference of Young’s modulus values in the unit cell and super cells (2 × 2 × 2) is attributed to the locations of atoms. The calculated slope is a negative value in super cells (2 × 2 × 2). In the first aspect, it is clear that the slope is dependent on the planar density of diffracted planes, so the fitting based on the planar density of super cells with a matrix of eight unit cells could submit a better result of intercept [35]. It is because eight cells besides each other are completed and have more symmetry than two cells (Figure S8) [35]. In the second aspect, the reason for the positive slope in the unit cell and the negative slope in the super cells (2 × 2 × 2) is related to the defects (imperfections) including point defects (vacancies, substitutional and interstitials), line defects (screw and edge dislocation), surface defects (grain boundaries) and volume defects (lack of order of atoms due to amorphous region in a very tiny area). The effect of these imperfections is more effective in super cells (2 × 2 × 2), while the unit cell is more ideal and less affected by these imperfections. This means that when the density of atoms in planes is increased, lower forces for dislocation motion are required and the strength will be decreased and, consequently, in super cells (2 × 2 × 2) the slope is negative. In the case of super cells, when the number of atoms is increased, by the increase of planar density, the effect of dislocation motion is increased; the strength and Young’s modulus will be decreased so the slope is negative. The intercept of the fitting line is a value of Young’s modulus, which can show the Young’s modulus of the plane with zero planar density as a plane without any specific atom. Therefore, a discussion of defects and imperfections cannot be considered for such a plane without an atom at the origin and so, in this case, the Young’s modulus value of the unit cell is less than that of the super cells (2 × 2 × 2) and consequently the plane without an atom is more realistic in a smaller area of a unit cell than in a wider area of the super cells. This means that the intercept in a unit cell is closer to the real values of the Young’s modulus. As mentioned above, the planar density depends on the array and position of atoms into the plane and the situation of the planes. For illustration, according to Figure 4b, the planar density of (210) > (510) > (102) > (023) > (043), because sequencing of the planes is not related to the diffraction (XRD), but it depends on the planar density values. With this method, it can be possible to consider two or more planes with similar planar density values; for example, in super cells (2 × 2 × 2), planar density values of (040) and (331) are equal to 0.250. The control of the displacement and deformation process of the atoms in the planes are associated with the dislocation networks. The work or energy (W) for the movement of the atoms in each plane corresponds to Equation (20) [44,45].

W = Gb^2^l (20)

In this equation, G, b and l are shear modulus, Burgers vector and dislocation length, respectively. In addition, by merging Equations (20) and (21), Equation (22) can be registered. In Equations (21) and (22), E and ν are Young’s modulus and Poisson’s ratio tandemly.

(21) $$G=\frac{E}{2\left(1+\nu \right)}$$

(22) $$W=\frac{E}{2\left(1+\nu \right)}\;b^{2}l$$

There are several studies on the properties of hydroxyapatite, especially with regard to biocompatibility and bioactivity, and these properties are dependent on the identification and recognition of hydroxyapatite structures [46,47] For example, when atoms are widely spaced, such as corner atoms (Ca) in (111) super cells (2 × 2 × 2), atoms require higher values of applied force for approaching, so knowledge on the planes of hydroxyapatite structure can be helpful for doping metals, polymers and other ceramics, for aims such as the controlled release of protein and also the fabrication of bioactive monolithic fragments in the biomaterials industry [48,49]. In addition, the hydroxyl ion is in the center of each unit cell. The hydroxyl group in the center of the hydroxyapatite lattice is surrounded by three calcium ions per hexagon, forming a ring (six calcium ions). A chord is formed by the structure, as these rings are responsible for many properties of hydroxyapatite, especially the biocompatibility and the position of the hydroxyl group in the planes have considerable importance for doping mechanisms [15,50].

### 3.2. Williamson-Hall Method in USDM Model

**Figure 5:**
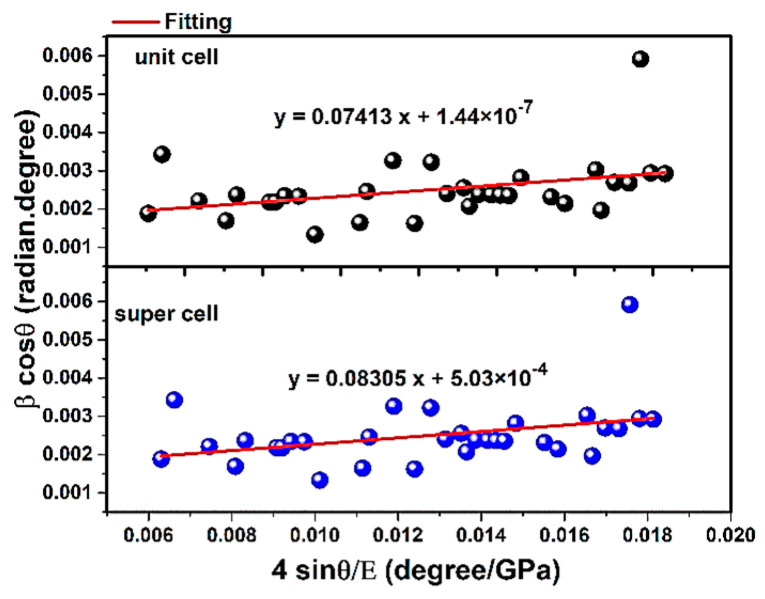
W-H in USDM model and plot of unit cell and super cells (2 × 2 × 2) of hydroxyapatite.

The W-H method has been used for determining different elastic properties. The best procedure is to mathematically reduce the errors and obtain the values of the Young’s modulus by all the diffracted peaks, using the least squares method. The W-H method is a simplified integral expanse and, taking into account the peak width, strain-induced broadening is specified [51]. Taking into account the W-H method in the USDM model, Young’s modulus values are examined. It is clear that in Equation (23) and Figure 5, the term of $\frac{4\sin\theta }{E_{\mathrm{hkl}}}$ is along the X-axis and the term of $\beta_{\mathrm{hkl}}.\cos\theta$ is along the Y-axis individually.

(23) $$\beta_{\mathrm{hkl}}.\cos ϴ=(\frac{K\lambda }{L})+4\sigma \frac{\sin ϴ}{E_{\mathrm{hkl}}}$$

Here, $\beta_{\mathrm{hkl}}$ is the broadening peak from (hkl) plane and, in this study, the instrumental broadening is taken as 0.02 degree for each diffracted peak and is subtracted from $\beta_{\mathrm{hkl}}$ values before multiplying to $\frac{\pi }{180}$ to convert the degree to radian. In this model, the condition is performed to calculate the strain and the average Young’s modulus. The average E value has been calculated in the research and studies on using the W-H method, but it is subject to errors, because if the average values of the Young’s modulus are considered (through Equation (19)), the final value would be far from the standard value of Young’s modulus in each peak extracted by X-ray diffraction. In addition, in some studies the Young’s modulus value is considered a value that is listed in the literature but is not associated with the prepared materials. As an illustration, based on the study in Reference [10], which refers to the use of the W-H method to calculate the crystal size and Scherrer analysis of hydroxyapatite, the average value of Young’s modulus has a larger deviation than the actual value of the Young’s modulus of hydroxyapatite. The line broadening of the diffracted peak is extracted with Crystal Diffract 6.7.2.300 software. According to Figure 5, the slope values are associated with the stress (σ). The values obtained were positive, and the positive values of intrinsic strain and stress can prove the tensile stress and strain, and if the values were negative, they are associated with compressive stress and strain. Additionally, the resulting values of stress (σ) and strain (ε) by utilizing the W-H method in the USDM model are shown in Table 6. The value of σ is in good agreement with the values obtained from Reference [52].

## 4. Conclusions

In this study, crystal of hydroxyapatite was successfully synthesized with the thermal treatment process. Moreover, the position of atoms and extracted planar density values of each diffracted plane (32 planes) of hydroxyapatite were determined and calculated for the first time. In addition, a new method based on the relationship between the measurement of elastic modulus and atomic planar density of crystalline hydroxyapatite, consisting of a unit cell and super cells (2 × 2 × 2), was fully presented. According to the method of this study, the slope of modules of elasticity against planar density in super cells (2 × 2 × 2) was negative; the reason was related to imperfections, and this means that when the density of atoms in the planes is increased, lower forces are required for the dislocation motion. As a result, a plane without an atom is more realistic in a smaller area of the unit cell than a larger area of the super cells (2 × 2 × 2), and this means that the intercept of the unit cell is closer to the real values of the Young’s modulus in the hydroxyapatite lattice. Moreover, a comparison of theoretical and experimental data of the Young’s modulus of hydroxyapatite showed that there is a small difference between the values for both unit cell and super cells (2 × 2 × 2), namely 10.47 GPa and 10.57 GPa, respectively; this means that the theoretical calculation is valid and, by decreasing by about 10 GPa, the experimental value can be obtained. Furthermore, the Young’s modulus values of hydroxyapatite in the unit cell and super cells (2 × 2 × 2) were achieved at 108.15 and 121.17 GPa tandemly. Finally, one of the applications of the presented method was carried out in this study and the Williamson-Hall method in the USDM model can be used to minimize the errors in the least squares method and to obtain the correct elastic modulus of hydroxyapatite, which is much more accurate than the average value.

## Figures and Tables

**Table 1:** Crystallographic parameters of hydroxyapatite structure.

Crystal System	a (Å)	c (Å)	Cell $\mathrm{Volume}\;(Å{)}^{3}$	Crystal Density (g/cm^3^)	Space Group
HCP	9.400	6.930	530.301	3.140	P6_3_/m

**Table 2:** Theoretical and experimental values of the Elastic constant of hydroxyapatite.

Stiffness Constant (C), (Gpa)	Theory. from Ref [40] (Ching et al.)	Theory. from Ref [41] (Leeuw et al.)	Experiment. from Ref [42] (Katz et al.)	Theory. This Study	Experiment. This Study
$C_{11}$	140.00	134.40	137.00	139.58	135.78
$C_{12}$	42.40	48.90	42.50	48.03	49.21
$C_{13}$	58.30	68.50	54.90	61.22	56.62
$C_{33}$	174.80	184.70	172.00	181.08	179.22
$C_{44}$	47.50	51.40	39.60	50.93	41.73

**Table 3:** Theoretical and experimental values of the Elastic compliance of hydroxyapatite.

Compliance Constant (S), (Gpa)	Theory. (Ching et al.)	Theory. (Leeuw et al.)	Experiment. (Katz et al.)	Theory. This Study	Experiment. This Study
$S_{11}$	0.008607002	0.009621806	0.008752330	0.008881114	0.009126549
$S_{12}$	−0.001638900	−0.002074100	−0.001829681	−0.002041879	−0.002424797
$S_{13}$	−0.002324030	−0.002799231	−0.002209613	−0.002312227	−0.002117248
$S_{33}$	0.007271063	0.007490496	0.007224509	0.007085868	0.006917516
$S_{44}$	0.021052632	0.019455253	0.025252525	0.019634793	0.023963575

**Table 4:** Resulted values of time for transverse and longitudinal waves of samples.

Number of Measurement	$t_{s}\;(µs)$	$t_{l}\;(µs)$	L (mm)
1	6.26	2.52	11.59
2	6.27	2.50	11.61
3	6.26	2.51	11.59
4	6.25	2.53	11.57
5	6.24	2.54	11.55

**Table 5:** Young’s modulus values of unit cell and super cell lattices of hydroxyapatite.

Study	Young Modulus (E), (Gpa) in This Method (Intercept Value)	
	Unit Cell	Super Cells (2 × 2 × 2)
Theory. (Ching et al.)	118.43	127.51
Theory. (Leeuw et al.)	113.94	128.91
Experiment. (Katz et al.)	109.54	117.52
Theory. (This Study)	118.62	131.74
Experiment. (This Study)	108.15	121.17

**Table 6:** Mechanical properties values of unit cell and super cells (2 × 2 × 2) of hydroxyapatite.

Mechanical Properties			
Structure	σ (GPa)	ε	$E^{a}$ (GPa)
**Unit cell**	0.07413	0.00068	108.15
**Super cells (2 × 2 × 2)**	0.08305	0.00068	121.17

a: Experiments of Young’s modulus values (This Study).

## Data Availability

Data sharing is not applicable.

## Supplementary Materials

The following are available online at https://www.mdpi.com/article/10.3390/ma14112949/s1, Figure S1: Images of production route of hydroxyapatite obtained from cow bones, Table S1: Crystallographic parameters of XRD pattern related to the natural hydroxyapatite obtained from cow bones, Figure S2: EDX spectrum and stoichiometric composition of hydroxyapatite obtained from cow bones, Figure S3: (a) Raw hexagonal structure, (b) fundamental axis, (c) principal crystallographic directions and (d) between the orthogonal axes and crystallographic directions, Figure S4: Transfer of hydroxyapatite unite cell in P63/m to P63 space group, Figure S5: Schematic of a design of cutted samples, Table S2: The values of Longitudinal and Transvers velocity of samples, Figure S6: Geometry and the situation of involved atoms in diffracted planes related to the unit-cell of hydroxyapatite (a) 210, (b) 211, (c) 112, (d) 300, (e) 202, (f) (301), (g) (130), (h) (310), (i) (032), (j) (040), (k) (023), (l) (222), (m) (320), (n) (230), (o) (213), (p) (321), (q) (042), (r) (033), (s) (004), (t) (050), (u) (501), (v) (331), (w) (043), (x) (124), (y) (510), (z) (511), (a1) (332) and (b1) (143), Figure S7: Geometry and the situation of involved atoms in diffracted planes related to the super-cell of hydroxyapatite (a) 210, (b) 211, (c) 112, (d) 300, (e) 202, (f) (301), (g) (130), (h) (310), (i) (032), (j) (040), (k) (023), (l) (222), (m) (320), (n) (230), (o) (213), (p) (321), (q) (042), (r) (033), (s) (004), (t) (050), (u) (501), (v) (331), (w) (043), (x) (124), (y) (510), (z) (511), (a1) (332) and (b1) (143), Table S3: Young’s modulus values of hydroxyapatite related to the literatures and present study, Figure S8: (a) The un-symmetry of two unit cells and (b) symmetry of eight unit cells of hydroxyapatite.

## References

[1] Sasmita F., Wibisono G., Judawisastra H., Priambodo T.A. (2018). Determination of elastic modulus of ceramics using ultrasonic testing. AIP Conf. Proc..

[2] Shimada M., Matsushita K., Kuratani S., Okamoto T., Koizumi M., Tsukuma K., Tsukidate T. (1984). Temperature dependence of young’s modulus and internal friction in alumina, silicon nitride, and partially stabilized zirconia ceramics. J. Am. Ceram. Soc..

[3] Vallet-Regí M. (2006). Revisiting ceramics for medical applications. Dalton Trans..

[4] Marzieh R., Sohrab N., Arvydas P., Giedrius J. (2020). Preparation and investigation of bioactive organic-inorganic nano-composite derived from PVB-co-VA-co-VAc/HA. Proceedings of the 15th International Conference Mechatronic Systems and Materials, MSM 2020.

[5] Rabiei M., Palevicius A., Monshi A., Nasiri S., Vilkauskas A., Janusas G. (2020). Comparing methods for calculating nano crystal size of natural hydroxyapatite using X-ray diffraction. Nanomaterials.

[6] Shih W.J., Wang M.C., Hon M.H. (2005). Morphology and crystallinity of the nanosized hydroxyapatite synthesized by hydrolysis using cetyltrimethylammonium bromide (CTAB) as a surfactant. J. Cryst. Growth.

[7] Loganathan T.M., Sultan M.T.H., Gobalakrishnan M.K. (2018). Ultrasonic inspection of natural fiber-reinforced composites. Sustainable Composites for Aerospace Applications.

[8] Nath D., Singh F., Das R. (2020). X-ray diffraction analysis by Williamson-Hall, Halder-Wagner and size-strain plot methods of CdSe nanoparticles—A comparative study. Mater. Chem. Phys..

[9] Khorsand Zak A., Abd Majid W.H., Abrishami M.E., Yousefi R. (2011). X-ray analysis of ZnO nanoparticles by Williamson-Hall and size-strain plot methods. Solid State Sci..

[10] Venkateswarlu K., Chandra Bose A., Rameshbabu N. (2010). X-ray peak broadening studies of nanocrystalline hydroxyapatite by WilliamsonHall analysis. Phys. B Condens. Matter.

[11] Bahrololoom M., Javidi M., Javadpour S. (2009). Characterisation of natural hydroxyapatite extracted from bovine cortical bone ash. J. Ceram. Process. Res..

[12] Shahabi S., Najafi F., Majdabadi A., Hooshmand T., Haghbin Nazarpak M., Karimi B., Fatemi S.M. (2014). Effect of gamma irradiation on structural and biological properties of a PLGA-PEG-hydroxyapatite composite. Sci. World J..

[13] Pasero M., Kampf A.R., Ferraris C., Pekov I.V., Rakovan J., White T.J. (2010). Nomenclature of the apatite supergroup minerals. Eur. J. Mineral..

[14] Hench L.L. (2013). An Introduction to Bioceramics.

[15] Lin K., Chang J. (2015). Structure and properties of hydroxyapatite for biomedical applications. Hydroxyapatite (Hap) for Biomedical Applications.

[16] Bystrov V.S., Coutinho J., Bystrova A.V., Dekhtyar Y.D., Pullar R.C., Poronin A., Palcevskis E., Dindune A., Alkan B., Durucan C. (2015). Computational study of hydroxyapatite structures, properties and defects. J. Phys. D Appl. Phys..

[17] Rajkumar M., Sundaram N., Rajendran V. (2011). Preparation of size controlled, stoichiometric and bioresorbable hydroxyapatite nanorod by varying initial pH, Ca/P ratio and sintering temperature. Digest J. Nanomater. Biostruct..

[18] Landi E., Riccobelli S., Sangiorgi N., Sanson A., Doghieri F., Miccio F. (2014). Porous apatites as novel high temperature sorbents for carbon dioxide. Chem. Eng. J..

[19] Rabiei M., Palevicius A., Dashti A., Nasiri S., Monshi A., Vilkauskas A., Janusas G. (2020). Measurement Modulus of elasticity related to the atomic density of planes in unit cell of crystal lattices. Materials.

[20] Rajabi K., Hosseini-Hashemi S. (2018). Application of the generalized Hooke’s law for viscoelastic materials (GHVMs) in nanoscale mass sensing applications of viscoelastic nanoplates: A theoretical study. Eur. J. Mech. A Solids.

[21] Kanoun M.B., Goumri-Said S., Abdullah K. (2012). Theoretical study of physical properties and oxygen incorporation effect in nanolaminated ternary carbides 211-MAX phases. Advances in Science and Technology of Mn+1AXn Phases.

[22] Li Y., Thompson R.B. (1990). Relations between elastic constants Cij and texture parameters for hexagonal materials. J. Appl. Phys..

[23] Huntington H.B. (1958). The elastic constants of crystals. Solid State Phys. Adv. Res. Appl..

[24] Mah M., Schmitt D.R. (2003). Determination of the complete elastic stiffnesses from ultrasonic phase velocity measurements. J. Geophys. Res. Solid Earth.

[25] Neighbours J.R., Schacher G.E. (1967). Determination of elastic constants from sound-velocity measurements in crystals of general symmetry. J. Appl. Phys..

[26] ASTM E797/E797M-15 Standard Practice for Measuring Thickness by Manual Ultrasonic Pulse-Echo Contact Method. https://www.astm.org/Standards/E797.htm.

[27] Van Buskirk W.C., Cowin S.C., Ward R.N. (1981). Ultrasonic measurement of orthotropic elastic constants of bovine femoral bone. J. Biomech. Eng..

[28] ASTM E214-05 Standard Practice for Immersed Ultrasonic Testing by the Reflection Method Using Pulsed Longitudinal Waves (Withdrawn 2007). https://www.astm.org/Standards/E214.htm.

[29] ASTM E1001-16 Standard Practice for Detection and Evaluation of Discontinuities by the Immersed Pulse-Echo Ultrasonic Method Using Longitudinal Waves. https://www.astm.org/Standards/E1001.htm.

[30] Elliot S. (1998). The Physics and Chemistry of Solids.

[31] McHugh P.E. (2004). Introduction to crystal plasticity theory. Mechanics of Microstructured Materials.

[32] Pandech N., Sarasamak K., Limpijumnong S. (2015). Elastic properties of perovskite A TiO_3_ (A = Be, Mg, Ca, Sr, and Ba) and PbBO_3_ (B = Ti, Zr, and Hf): First principles calculations. J. Appl. Phys..

[33] Wang H., Prendiville P.L., McDonnell P.J., Chang W.V. (1996). An ultrasonic technique for the measurement of the elastic moduli of human cornea. J. Biomech..

[34] Bray D.E., Stanley R.K. (1996). Nondestructive Evaluation: A Tool in Design, Manufacturing and Service.

[35] Rabiei M., Palevicius A., Nasiri S., Dashti A., Vilkauskas A., Janusas G. (2021). Relationship between young’s modulus and planar density of unit cell, super cells (2 × 2 × 2), symmetry cells of perovskite (CaTiO_3_) LATTICE. Materials.

[36] Bodke M., Gawai U., Patil A., Dole B. (2018). Estimation of accurate size, lattice strain using Williamson-Hall models, SSP and TEM of Al doped ZnO nanocrystals. Mater. Tech..

[37] Figliola R.S., Beasley D.E. (2014). Theory and Design for Mechanical Measurements.

[38] Moradi K., Sabbagh Alvani A.A. (2020). First-Principles study on Sr-doped hydroxyapatite as a biocompatible filler for photo-cured dental composites. J. Aust. Ceram. Soc..

[39] Bhat S.S., Waghmare U.V., Ramamurty U. (2014). First-Principles study of structure, vibrational, and elastic properties of stoichiometric and calcium-deficient hydroxyapatite. Cryst. Growth Des..

[40] Ching W.Y., Rulis P., Misra A. (2009). Ab initio elastic properties and tensile strength of crystalline hydroxyapatite. Acta Biomater..

[41] De Leeuw N.H., Bowe J.R., Rabone J.A.L. (2007). A computational investigation of stoichiometric and calcium-deficient oxy- and hydroxy-apatites. Faraday Discuss..

[42] Katz J.L., Ukraincik K. (1971). On the anisotropic elastic properties of hydroxyapatite. J. Biomech..

[43] Zhang J.M., Zhang Y., Xu K.W., Ji V. (2007). Anisotropic elasticity in hexagonal crystals. Thin Solid Films.

[44] Berdichevsky V. (2016). Energy of dislocation networks. Int. J. Eng. Sci..

[45] Reed-Hill R.E., Abbaschian R. (1992). Physical Metallurgy Principles.

[46] Shi D., Jiang G., Bauer J. (2002). The effect of structural characteristics on the in vitro bioactivity of hydroxyapatite. J. Biomed. Mater. Res..

[47] Ragel C.V., Vallet-Regí M., Rodríguez-Lorenzo L.M. (2002). Preparation and in vitro bioactivity of hydroxyapatite/solgel glass biphasic material. Biomaterials.

[48] Dasgupta S., Banerjee S.S., Bandyopadhyay A., Bose S. (2010). Zn- and Mg-doped hydroxyapatite nanoparticles for controlled release of protein. Langmuir.

[49] Uysal I., Severcan F., Tezcaner A., Evis Z. (2014). Co-Doping of hydroxyapatite with zinc and fluoride improves mechanical and biological properties of hydroxyapatite. Prog. Nat. Sci. Mater. Int..

[50] Ptáček P. (2016). Apatites and Their Synthetic Analogues—Synthesis, Structure, Properties and Applications.

[51] Suryanarayana C., Norton M.G., Suryanarayana C., Norton M.G. (1998). Practical aspects of X-ray diffraction. X-ray Diffraction.

[52] Itatani K., Tsuchiya K., Sakka Y., Davies I.J., Koda S. (2011). Superplastic deformation of hydroxyapatite ceramics with B_2_O_3_ or Na_2_O addition fabricated by pulse current pressure sintering. J. Eur. Ceram. Soc..

