# Supplementary material for: X-ray Diffraction Analysis and Williamson-Hall Method in USDM Model for Estimating More Accurate Values of Stress-Strain of Unit Cell and Super Cells (2 × 2 × 2) of Hydroxyapatite, Confirmed by Ultrasonic Pulse-Echo Test
Source: Materials (Basel). 2021 May 30;14(11):2949. doi: 10.3390/ma14112949 (PMC8198306; doi:10.3390/ma14112949)
Supplement: Supplementary file 1 [file materials-14-02949-s001.zip › materials-1181177-supplementary.pdf]

materials

MDPI

Supplementary materials

X-Ray Diffraction Analysis and Williamson-Hall Method in USDM Model for Estimating More Accurate Values of Stress-Strain of Unit Cell and Super Cells (2 × 2 × 2) of Hydroxyapatite, Confirmed by Ultrasonic Pulse-Echo Test

Marzieh Rabiei [1,*], Arvydas Palevicius [1,*], Amir Dashti [2], Sohrab Nasiri [1], Ahmad Monshi [3], Akram Doustmohammadi [4], Andrius Vilkauskas [1] and Giedrius Janusas [1,*]

[1] Faculty of Mechanical Engineering and Design, Kaunas University of Technology, LT-51424 Kaunas, Lithuania; sohrab.nasiri@ktu.edu (S.N.); andrius.vilkauskas@ktu.lt (A.V.)
[2] Department of Materials Science and Engineering, Sharif University of Technology, Tehran 11365-9466, Iran; a.dashty@merc.ac.ir
[3] Department of Materials Engineering, Isfahan University of Technology, Isfahan 84154, Iran; dr.ahmad.monshi@gmail.com
[4] Materials and Energy Research Center (MERC), Meshkin-Dasht, Karaj 31787-316, Iran; hivadoostmohammadi91@gmail.com
* Correspondence: marzieh.rabiei@ktu.edu (M.R.); arvydas.palevicius@ktu.lt (A.P); giedrius.janusas@ktu.lt (G.J.)

Citation: Rabiei, M.; Palevicius, A.; Dashti, A.; Nasiri, S.; Monshi, A.; Doustmohammadi, A.; Vilkauskas, A.; Janusas, G. X-Ray Diffraction Analysis and Williamson-Hall Method in USDM Model for Estimating More Accurate Values of Stress-Strain of Unit Cell and Super Cells (2 × 2 × 2) of Hydroxyapatite, Confirmed by Ultrasonic Pulse-Echo Test. *Materials* **2021**, *14*, 2949. https://doi.org/10.3390/ma14112949

Academic Editor: Thomas Walter Cornelius

Received: 28 March 2021
Accepted: 28 May 2021
Published: 3 June 2021

Publisher's Note: MDPI stays neutral with regard to jurisdictional claims in published maps and institutional affiliations.

Copyright: © 2021 by the authors. Licensee MDPI, Basel, Switzerland. This article is an open access article distributed under the terms and conditions of the Creative Commons Attribution (CC BY) license (http://creativecommons.org/licenses/by/4.0/).

1. Materials and Experiments

In this study, a Bruker D8 Advance X-ray diffractometer with $Cu_{K_{\alpha}}$ radiation was used (Kaunas, Lithuania). The powder X-Ray diffraction was taken at 40 kV and 40 mA, and recorded from 20 to 70 degrees for 2θ at a scanning speed of 2.5 degrees/minute and a step size of 0.02 degrees. The XRD patterns were studied by High Score X'Pert software (version 4.9.0) analysis and exported ASC suffix and cif files. Natural bones of cow were prepared from a Maxima LT shop (according to the EU Regulation-Lithuanian breeds). A white and clean hydroxyapatite was obtained. The model of the ball mill was planetary Fritsch Pulverisette-5. The model of the furnace was E5CK-AA1-302 (Snol 6, 7/1300) (Lithuania, Kaunas). A pulse echo method was used for the measurement of sound velocity for both transverse and longitudinal ultrasonic waves, as well as the probs were used related to the size of specimens accordingly (Iran, Isfahan). The model pulser receiver was Panametrics Co (Waltham) and the osciloscope was an Iwatsu (100 MHz, Japan). In addition, the model of mechanical machines were CD04-Z and CIP, Model CP 360 tandemly (Iran, Tehran). Furthermore, three dimension (3D) geometry of crystal structures were designed by Crystal Maker software (Version 10.2.2). The theoritical calculations were done by utilizing materials studio 6.0 software at generalized gradient approximation (GGA) method, employing the Perdew-Burke-Ernzerhof (PBE) functional with dispersion correction basis set.

2. Preparation of Hydroxyapatite Powder

Bovine bones (femur) of cow (Maxima LT shop, Kaunas, Lithuania) were prepared and first of all they were boiled in hot water for two h to eliminate meats and fats on the surface of the bones. Then, immersed into the acetone for one h to remove collagen and fat (step 1, Figure S1). In next step, bones were washed by distillated water and dried two times. Then, bones were placed in separate steps into the furnace under ambient conditions and the rate of increasing temperature was 10 °C/min. In step 2, bones were fired at 950 °C for 2 h to allow diffusion of proteins, such as collagens, from inside of bones to the surface, and burning at high temperatures and finally, they were cooled inside the furnace

very slowly (step 3). Following this process, the first black fired bones (due to carbon release) turned into a white granular bulk. Furthermore, bones were transformed to fully crystallized hydroxyapatite at 950 °C [1]. Hydroxyapatite extracted from cow bones was placed into the planetary ball mill machine involving a bowl (tungsten carbide) and balls, to fabricate fine particles after heat-treating (step 4). The feed ratio was 30 g powder to 300 g of balls (1 to 10 weight ratio), the speed was fixed at 250 rpm and the milling time was adjusted at 2 h with pause and reverse mode, according to the previous citations of Ref [2,3]. In ball mill process, collision between balls and powders has provided plastic deformation of particles extremely, and with repeat of this process the nano crystalline powders will be created [4]. The images of production routes (summary) of hydroxyapatite are presented in Figure S1.

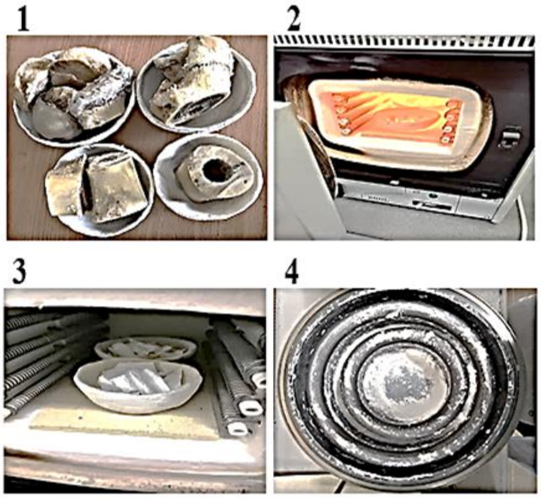

Figure S1. Images of production route of hydroxyapatite obtained from cow bones.

Table S1. Crystallographic parameters of XRD pattern related to the natural hydroxyapatite obtained from cow bones.

2θ (Degree)	β = FWHM (Degree)	θ (Degree)	cosθ (Degree)	1/cosθ (Degree)	Ln(1/cosθ) (Degree)	β = FWHM (Radian)	Ln β (Radian)	4 sinθ (Degree)	β(Radian).cosθ (Degree)	hkl	d_{hkl} (Å)
22.02	0.11	11.01	0.9816	1.01875	0.01858	0.00192	−6.25550	0.7639	0.00188	020	4.0703
23.13	0.20	11.565	0.9797	1.02072	0.02051	0.00349	−5.65766	0.8019	0.00342	111	3.8898
26.12	0.13	13.06	0.9741	1.02655	0.02621	0.00227	−6.08845	0.9039	0.00221	002	3.4650
28.35	0.10	14.175	0.9696	1.03140	0.03092	0.00175	−6.35081	0.9795	0.00169	102	3.1882
29.22	0.14	14.61	0.9677	1.03342	0.03287	0.00244	−6.01434	1.0090	0.00236	210	3.0769
31.93	0.13	15.965	0.9614	1.04012	0.03933	0.00227	−6.08845	1.1002	0.00218	211	2.8122
32.41	0.13	16.205	0.9603	1.04137	0.04054	0.00227	−6.08845	1.1163	0.00218	112	2.7890
33.18	0.14	16.59	0.9584	1.04344	0.04252	0.00244	−6.01434	1.1421	0.00234	300	2.7135
34.34	0.14	17.17	0.9554	1.04665	0.04559	0.00244	−6.01434	1.1808	0.00233	202	2.6384
35.70	0.08	17.85	0.9519	1.05057	0.04934	0.00140	−6.57395	1.2261	0.00133	301	2.5267
39.47	0.10	19.735	0.9413	1.06240	0.06053	0.00175	−6.35081	1.3507	0.00164	130	2.2578
40.05	0.15	20.025	0.9395	1.06435	0.06236	0.00262	−5.94534	1.3697	0.00246	310	2.2578
42.27	0.20	21.135	0.9327	1.07212	0.06964	0.00349	−5.65766	1.4423	0.00326	032	2.1364
44.11	0.10	22.055	0.9268	1.07895	0.07599	0.00175	−6.35081	1.5020	0.00162	040	2.0352
45.56	0.20	22.78	0.9220	1.08460	0.08121	0.00349	−5.65766	1.5488	0.00322	023	2.0090
46.91	0.15	23.455	0.9174	1.09007	0.08624	0.00262	−5.94534	1.5921	0.00240	222	1.9449
48.36	0.16	24.18	0.9123	1.09618	0.09183	0.00279	−5.88081	1.6384	0.00255	320	1.8676
48.85	0.13	24.425	0.9105	1.09829	0.09376	0.00227	−6.08845	1.6540	0.00207	230	1.8676
49.72	0.15	24.86	0.9073	1.10213	0.09724	0.00262	−5.94534	1.6816	0.00238	213	1.8473
50.78	0.15	25.39	0.9034	1.10692	0.10158	0.00262	−5.94534	1.7151	0.00237	321	1.8033
51.56	0.15	25.78	0.9005	1.11053	0.10484	0.00262	−5.94534	1.7397	0.00236	042	1.7549

52.33	0.15	26.165	0.8975	1.11417	0.10811	0.00262	−5.94534	1.7638	0.00235	033	1.7590
53.39	0.18	26.695	0.8934	1.11931	0.11271	0.00314	−5.76302	1.7970	0.00281	004	1.7325
56.10	0.15	28.05	0.8825	1.13310	0.12495	0.00262	−5.94534	1.8810	0.00231	050	1.6281
57.36	0.14	28.68	0.8773	1.13984	0.13089	0.00244	−6.01434	1.9197	0.00214	501	1.5850
60.16	0.20	30.08	0.8653	1.15563	0.14465	0.00349	−5.65766	2.0048	0.00302	331	1.5281
60.65	0.13	30.325	0.8632	1.15851	0.14714	0.00227	−6.08845	2.0196	0.00196	043	1.5270
61.90	0.18	30.95	0.8576	1.16602	0.15360	0.00314	−5.76302	2.0572	0.00269	124	1.5096
63.26	0.18	31.63	0.8515	1.17446	0.16081	0.00314	−5.76302	2.0977	0.00267	510	1.4621
64.32	0.40	32.16	0.8466	1.18125	0.16657	0.00698	−4.96452	2.1291	0.00591	511	1.4306
65.29	0.20	32.645	0.8420	1.18761	0.17194	0.00349	−5.65766	2.1577	0.00294	332	1.4275
66.64	0.20	33.32	0.8356	1.19672	0.17959	0.00349	−5.65766	2.1973	0.00292	143	1.4082

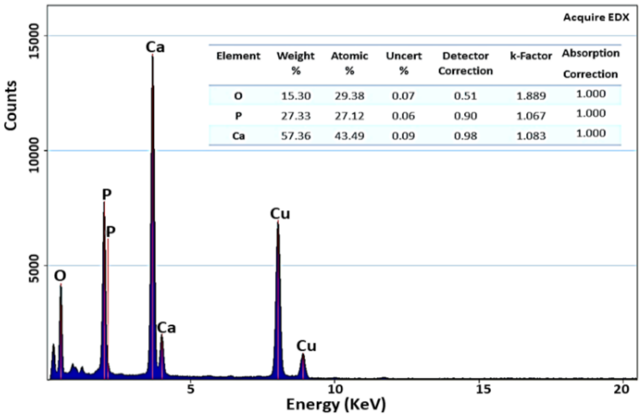

Figure S2. EDX spectrum and stoichiometric composition of hydroxyapatite obtained from cow bones.

3. Elastic Stiffness Constant and Elastic Compliance

The crystallographic nature of the hexagonal structures is shown in Figure S3. Forthermore, in Figure S3a, the unit cell has two axes a_1 and a_2 of equal length inclined at 60°, and an orthogonal axis of different length of c. Figure S3c, submits the principal crystallographic directions in the (0001) that fundamental plane expressed in the Miller-Bravais system, utilizing four axes composed of three planar a_1 axes at 120° to each other and the orthogonal c axis [5]. Figure S3d presents the relationship between the orthogonal axes and crystallographic directions. In Figure S3a, the unit cell has two axes a_1 and a_2 of equal length inclined at 60°, and an orthogonal axis of different length of c. Figure S3c, submits the principal crystallographic directions in the (0001) that fundamental plane expressed in the Miller-Bravais system, utilizing four axes composed of three planar a_1 axes at 120° to each other and the orthogonal c axis [6]. Figure S3d presents the relationship between the orthogonal axes and crystallographic directions. When ascribing and measuring compliance and stiffness constants it is most important that the orthogonal axes a_1, a_2 and c conform to standardised orthogonal directions in the hexagonal crystals. These directions are shown in Figure S3d. Mostly, S and C constants are reported with respect to these axial directions. For example σ_{33} and ε_{33} are attribuated to the tensile stresses and strains in the [0001] direction and S_{33} and C_{33} are related to the compliance and stiffness constants, measured in the [0001] direction. Generally, elastic compliance is derivative of strain with respect to stress or derivative of displacement with respect to force and it is demonstrated with sign of S_{ij}.

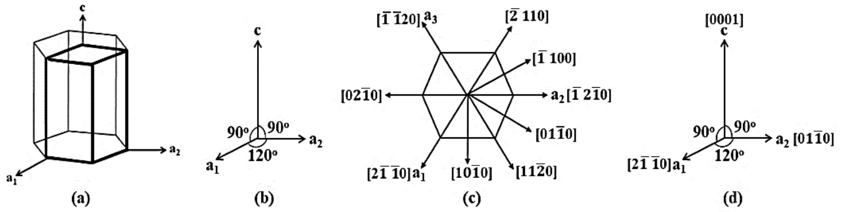

Figure S3. (**a**) Raw hexagonal structure, (**b**) fundamental axis, (**c**) principal crystallographic directions and (**d**) between the orthogonal axes and crystallographic directions.

4. Computational Details

All geometry optimization and elastic constant calculation was done by CASTEP module of Material studio 6.0. For exchange-correlation interactions we used generalized gradient approximation (GGA) level, employing the Perdew-Burke-Ernzerhof (PBE) functional. For geometric optimization step, the semi-empirical Tkatchenko and Scheffler (TS) dispersion correction was used to consider van der Waals' interactions. Unit cell is extracted from CIF data file of Wilson et al. works [7]. Extracted unit cell is shown in Figure S4. Due to the non-physical duplication of each OH group in the unit cell, the symmetry should be reduced to the P63 space group before simulation. This modification has been used before in many theoretical studies of HA [8–10].

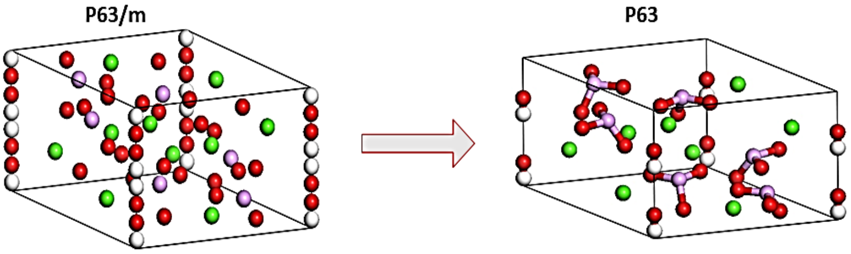

Figure S4. Transfer of hydroxyapatite unite cell in P63/m to P63 space group.

To calculate the accurate density of the electronic states, Brillouin zone integrations were performed using Monkhorst and Pack k-point meshes (4 × 3 × 3). We considered 380 eV energy cutoff for wave function expansion. Self-consistent field tolerance was set to 10^{-6} eV/atom. The convergence criterion of total force acting on each atom was set below 0.008 eV/Å. The maximum displacement and stress were 10^{-4} nm and 0.015 GPa, respectively.

5. Ultrasonic Method

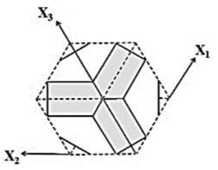

Figure S5. Schematic of a design of cutted samples.

Table S2. The values of Longitudinal and Transvers velocity of samples.

Longitudinal Velocity (m/s)	Transvers Velocity (m/s)	Quasi-Longitudinal or Quasi-Transverse (m/s)
$V_{1/1}$ = 6798.42	$V_{2/3}$ = 3769.01	$V_{12/12}$ = 3845.09
$V_{2/2}$ = 6813.51	$V_{1/3}$ = 3825.23	$V_{13/13}$ = 4095.23
$V_{3/3}$ = 7810.39	$V_{1/2}$ = 3838.61	$V_{23/23}$ = 3983.65

6. Calculations of Planar Density of (a) (020) Unit Cell, (b) (020) Super Cell, (c) (111) Unit Cell, (d) (111) Super Cell, (e) (002) Unit Cell, (f) (002) Super Cell, (g) (102) Unit Cell and (h) (102) Super Cell

(a) (020), Unit Cell

area of the (020): $s = a \times c = 9.4 \times 6.93 = 65.142\ \mathrm{\mathring{A}}^2$

number of atoms in the plane (020) × area of each atom in the plane (020) $= \left[\underbrace{\left(\frac{108}{138} \times 2\right)}_{\text{O atom no.1}} + \underbrace{\left(\frac{62}{138} \times 2\right)}_{\text{O atom no.2}}\right] \times \pi \times (1.4)^2 = 15.171\ \mathrm{\mathring{A}}^2$

$$\text{Planar density} = \frac{[\text{number of atoms in plane(020)} \times \text{area of each atom in the plane(020)}]}{\text{area of the plane (020)}} = \frac{15.171}{65.142} = 0.233$$

(b) (020), Super Cell

area of the (020): $s = (2a) \times (2c) = (18.8) \times (2 * 6.93) = 260.568\ \mathrm{\mathring{A}}^2$

number of atoms in the plane (020) × area of each atom in the plane (020) $= \left[\underbrace{(8 \times 1)}_{\text{O atoms center}} + \underbrace{\left(16 \times \frac{1}{2}\right)}_{\text{O atoms edge}}\right] \times \pi \times (1.4)^2 + \underbrace{\left[8 \times \frac{40}{50}\right]}_{\text{Ca atoms}} \times \pi \times (1)^2 + \left[\underbrace{(8 \times 1)}_{\text{H atoms center}} + \underbrace{\left(16 \times \frac{1}{2}\right)}_{\text{H atoms edge}}\right] \times \pi \times (0.1)^2 = 98.52 + 20.106 + 0.503 = 11.129\ \mathrm{\mathring{A}}^2$

$$\text{Planar density} = \frac{[\text{number of atoms in plane(020)} \times \text{area of each atom in the plane (020)}]}{\text{area of the plane (020)}} = \frac{119.1299}{260.568} = 0.457$$

(c) (111), Unit Cell

cosine's rule: $x^2 = a^2 + a^2 - 2(a)(a)\cos 120^\circ = 265.08 \rightarrow x = 16.231$

$y^2 = a^2 + c^2 = (9.4)^2 + (6.93)^2 = 136.385 \rightarrow y = 11.678$

area of the plane(111): $s \xrightarrow{\text{Heron's law}} s = \sqrt{p(p-y)(p-y)(p-x)}$

$$p = \frac{x + y + y}{2} = \frac{16.231 + (2 \times 11.678)}{2} = 19.819$$

$$s = \sqrt{(19.819)(19.819 - 11.678)^2(19.819 - 16.281)} = 68.171\ \mathrm{\mathring{A}}^2$$

number of atoms in the plane(111) × area of each atom in the plane(111)=

$\left[\underbrace{\left(\frac{28}{97} \times \frac{139}{148}\right)}_{\text{O atom no.1}} + \underbrace{\left(\frac{34}{97}\right)}_{\text{O atom no.2}} + \underbrace{\left(\frac{78}{97}\right)}_{\text{O atom no.3}} + \underbrace{\left(\frac{56}{97}\right)}_{\text{O atom no.4}} + \underbrace{\left(\frac{94}{97}\right)}_{\text{O atom no.5}} + \underbrace{\left(\frac{44}{97}\right)}_{\text{O atom no.6}}\right] \times \pi \times (1.4)^2 + \underbrace{\left[\left(0.5 \times \frac{60}{67}\right) \times \pi \times (1)^2\right]}_{\text{Ca atom}} + \underbrace{\left(\frac{28}{35}\right) \times \pi \times (0.31)^2}_{\text{P atom}}$

$= 21.094 + 1.407 + 0.242 = 22.743\ \mathrm{\mathring{A}}^2$

$$\text{Planar density} = \frac{[\text{number of atoms in plane(111)} \times \text{area of each atom in the plane(111)}]}{\text{area of the plane (111)}} = \frac{22.743}{68.171} = 0.334$$

(d) (111), Super Cell

cosine's rule: $x^2 = (2a)^2 + (2a)^2 - 2(2a)(2a)\cos 120^{\circ} = 12a^2 \rightarrow x = 2\sqrt{3}a \quad x = 32.563$

$y^2 = (2a)^2 + (2c)^2 = (18.8)^2 + (2 \times 6.93)^2 = 545.540 \rightarrow y = 23.357$

area of the plane (111): $s \xrightarrow{\text{Heron's law}} s = \sqrt{p(p-y)(p-y)(p-x)}$

$$p = \frac{x+y+y}{2} = \frac{32.563 + 23.357 + 23.357}{2} = 39.638$$

$$s = \sqrt{39.638(39.638 - 23.357)^2(39.638 - 32.563)} = 272.704\ \mathring{A}^2$$

number of atoms in the plane (111) × area of each atom in the plane (111)=

$$\Big[\underbrace{\Big(2 \times \frac{28}{97} \times \frac{139}{148}\Big)}_{\text{O atom no.1}} + \underbrace{\Big(\frac{34}{97} \times 4\Big)}_{\text{O atom no.2}} + \underbrace{\Big(\frac{78}{97} \times 4\Big)}_{\text{O atom no.3}} + \underbrace{\Big(\frac{56}{97} \times 4\Big)}_{\text{O atom no.4}} + \underbrace{\Big(\frac{94}{97} \times 4\Big)}_{\text{O atom no.5}} + \underbrace{\Big(\frac{44}{97} \times 5\Big)}_{\text{O atom no.6}} + \underbrace{\Big(\frac{28}{97}\Big)}_{\text{O atom no.7}}\Big] \times \pi \times (1.4)^2$$

$$+ \Big[\underbrace{\Big(2 \times 0.5 \times \frac{60}{67}\Big)}_{\text{Ca atom no.1}} + \underbrace{(2 \times 1)}_{\text{Ca atom no.2}}\Big] \times \pi \times (1)^2 + \Big[\underbrace{\Big(\frac{14}{18} \times 3\Big)}_{\text{P atom no.1}} + \underbrace{\Big(\frac{12}{18}\Big)}_{\text{P atom no.2}}\Big] \times \pi \times (0.31)^2$$

$$= 85.608 + 9.097 + 0.906 = 95.611\ \mathring{A}^2$$

$$\text{Planar density} = \frac{[\text{number of atoms in plane(111)} \times \text{area of each atom in the plane(111)}]}{\text{area of the plane (111)}} = \frac{95.611}{272.704} = 0.351$$

(e) (002), Unit Cell

area of the (002): $s = a^2 \times \sin 60° = (9.4)^2 \times \sin 60° = 76.522\ \mathring{A}^2$

number of atoms in the plane (002) × area of each atom in the plane (002)

$$= \underbrace{[2]}_{\text{Ca atoms}} \times \pi \times (1)^2 + \underbrace{\Big[6 \times \frac{10}{81}\Big]}_{\text{O atoms}} \times \pi \times (1.4)^2 = 2 \times \pi + \frac{60}{81} \times \pi \times (1.4)^2 = 10.844\ \mathring{A}^2$$

$$\text{Planar density} = \frac{[\text{number of atoms in plane(002)} \times \text{area of each atom in the plane(002)}]}{\text{area of the plane (002)}} = \frac{10.844}{76.522} = 0.142$$

(f) (002), Super Cell

area of the (002): $s = (2a)^2 \times (2a) \times \sin 120° = (18.8) \times (18.8) \times 0.866 = 306.088\ \mathring{A}^2$

number of atoms in the plane (002) ×area of each atom in the plane(002)

$$= \underbrace{8 \times 1^2 \times \pi}_{\text{Ca atoms}} + \underbrace{\Big[24 \times \frac{10}{81}\Big] \times \pi \times (1.4)^2}_{\text{O atoms}} = 43.377\ \mathring{A}^2$$

$$\text{Planar density} = \frac{[\text{number of atoms in plane(002)} \times \text{area of each atom in the plane(002)}]}{\text{area of the plane (002)}} = \frac{43.377}{306.88} = 0.142$$

(g) (102), Unit Cell

$$\text{cosine's rule: } x^2 = a^2 + \left(\frac{c}{2}\right)^2 = (9.4)^2 + \left(\frac{6.93}{2}\right)^2 = 100.366 \rightarrow x = 10.018$$

$$y^2 = a^2 + \left(\frac{c}{2}\right)^2 \rightarrow y = 10.018$$

$$\text{area of the plane(102):s} \xrightarrow{\text{Heron's law}} s = \sqrt{p(p-x)(p-y)(p-a)}$$

$$p = \frac{x+y+a}{2} = \frac{10.018 + 10.018 + 9.4}{2} = 14.718$$

$$s = \sqrt{14.718(14.718-10.018)(14.718-10.018)(14.718-9.4)} = 41.581\ \mathrm{A}^{\circ 2}$$

number of atoms in the plane (102) × area of each atom in the plane (102)=

$$\left[\underset{\substack{\text{atom}\\ \text{no.1}}}{\left(\frac{21}{59}\right)} + \underset{\substack{\text{atom}\\ \text{no.2}}}{\left(\frac{46}{59}\right)} + \underset{\substack{\text{atom}\\ \text{no.3}}}{\left(\frac{39}{59}\right)} + \underset{\substack{\text{atom}\\ \text{no.3}}}{\left(\frac{33}{59}\right)}\right] * \pi * (1.4)^2 = 14.507\ \mathrm{A}^{\circ 2}$$

$$\text{Planar density} = \frac{[\text{number of atoms in plane(102)} \times \text{area of each atom in the plane(102)}]}{\text{area of the plane (102)}} = \frac{14.517}{41.581} = 0.349$$

(h) (102), Super Cell

$$\text{cosine's rule: } x^2 = (2a)^2 + (c)^2 = (18.8)^2 + (6.93)^2 = 401.465 \rightarrow x = 20.04$$

$$y^2 = (2a)^2 + (c)^2 = (18.8)^2 + (6.93)^2 = 401.465 \rightarrow y = 20.04$$

$$\text{Area of the plane (102):s} \xrightarrow{\text{Heron's law}} s = \sqrt{p(p-x)(p-y)(p-a)}$$

$$p = \frac{x+y+2a}{2} = \frac{20.04 + 20.04 + 18.8}{2} = 29.44$$

$$s = \sqrt{29.44(29.44-20.04)^2(29.44-18.18)} = 166.367\ \mathrm{A}^{\circ 2}$$

number of atoms in the plane (102) × area of each atom in the plane(102) =

$$\left[\underset{\substack{\text{O atom}\\ \text{no.1}}}{\left(2\times\frac{21}{59}\right)} + \underset{\substack{\text{O atom}\\ \text{no.2}}}{\left(2\times\frac{46}{59}\right)} + \underset{\substack{\text{O atom}\\ \text{no.3}}}{\left(2\times\frac{39}{59}\right)} + \underset{\substack{\text{O atom}\\ \text{no.4}}}{\left(2\times\frac{33}{59}\right)} + \underset{\substack{\text{O atom}\\ \text{no.5}}}{\left(\frac{52}{59}\right)} + \underset{\substack{\text{O atom}\\ \text{no.6}}}{\left(\frac{54}{59}\right)} + \underset{\substack{\text{O atom}\\ \text{no.7}}}{\left(2\times\frac{1}{59}\right)}\right] \times \pi \times (1.4)^2 = 40.285\ \mathrm{A}^{\circ 2}$$

$$\text{Planar density} = \frac{[\text{number of atoms in plane(102)} \times \text{area of each atom in the plane(102)}]}{\text{area of the plane (102)}} = \frac{40.285}{166.367} = 0.242$$

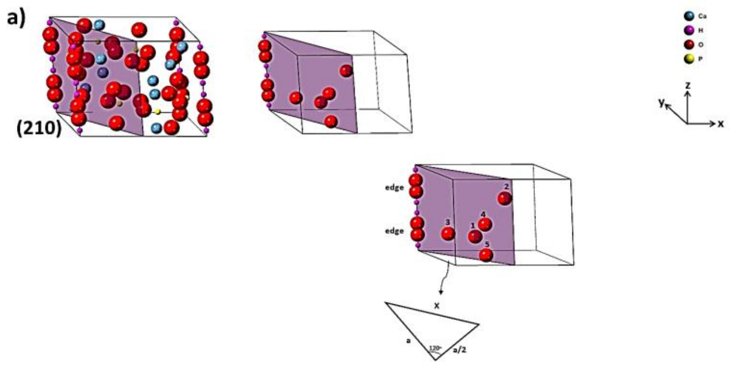

Law of Cosines: $x^2 = a^2 + \left(\frac{a}{2}\right)^2 - 2(a)\left(\frac{a}{2}\right)cos120^{\circ} = (9.4)^2 + \left(\frac{9.4}{2}\right)^2 - 2(9.4)\left(\frac{9.4}{2}\right)cos120^{\circ} = 154.63 \rightarrow x = 12.435\ A^{\circ}$

Area of the plane (210): $s = x \times c = 12.435 * 6.93 = 86.175\ A^{\circ 2}$

Number of atoms in the plane (210)×area of each atom in the plane (210)

$$=\Big[\underbrace{\underbrace{\left(4\times\frac{1}{2}\right)}_{\text{Edge atom}} + \underbrace{\left(\frac{15}{61}\right)}_{\text{atom no.1}} + \underbrace{\left(\frac{36}{61}\right)}_{\text{atom no.2}} + \underbrace{\left(\frac{58}{61}\right)}_{\text{atom no.3}} + \underbrace{\left(\frac{42}{61}\right)}_{\text{atom no.4}} + \underbrace{\left(\frac{42}{61}-\frac{9}{80}\right)}_{\text{atom no.5}}}_{\text{O atom}}\Big] \times \pi \times (1.4)^2 +$$

$$\underbrace{\left[\left(4\times\frac{1}{2}\right)\times\pi\times(0.1)^2\right]}_{\text{H atoms}} = [5.05\times\pi\times(1.4)^2 + 2\pi(0.1)^2] = 31.104 + 0.063 =$$

$31.167\ A^{\circ 2}$

Planar density= $\frac{[Number\ of\ atoms\ in\ plane\ (210)\times area\ of\ each\ atom\ in\ the\ plane\ (210)]}{area\ of\ the\ plane\ (210)} = \frac{31.167}{86.175} = 0.362$

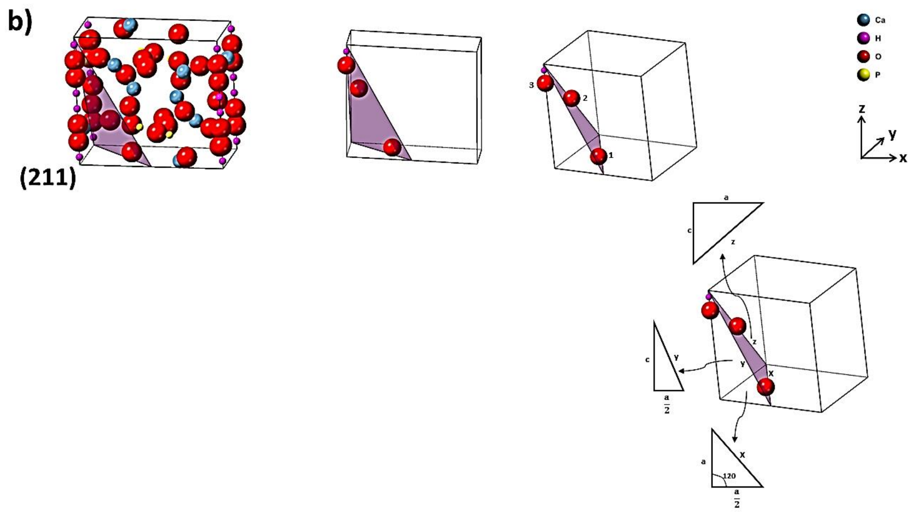

Cosines law: $x^2 = a^2 + \left(\frac{a}{2}\right)^2 - 2(a)\left(\frac{a}{2}\right) cos120^\circ = (9.4)^2 + \left(\frac{9.4}{2}\right)^2 - 2(9.4)\left(\frac{9.4}{2}\right) cos120^\circ = 154.63 \rightarrow x = 12.435$

$$y^2 = c^2 + \left(\frac{a}{2}\right)^2 = (6.93)^2 + \left(\frac{9.4}{2}\right)^2 = 70.115 \rightarrow y = 8.373$$

$$Z^2 = c^2 + a^2 = (6.93)^2 + (9.4)^2 = 136.385 \rightarrow z = 11.678$$

Area of the plane (211): $s \xrightarrow{Hero\,n'\,s\;law} s = \sqrt{p(p-x)(p-y)(p-z)}$

$$p = \frac{x+y+y}{2} = \frac{12.435 + 8.373 + 11.678}{2} = 16.243$$

$$s = \sqrt{16.243(16.243 - 12.435)(16.243 - 8.373)(16.243 - 11.678)}$$

$$\rightarrow s = 47.14\ A^{\circ 2}$$

Number of atoms in the plane (211)×area of each atom in the plane (211)

$$= \Bigg[\underbrace{\underset{\text{atom no.1}}{\frac{94}{100}} + \underset{\text{atom no.2}}{\frac{60}{100}} + \underset{\text{atom no.3}}{\frac{8}{100}}}_{\text{O atoms}}\Bigg] \times \pi \times (1.4)^2 + \underbrace{\frac{1}{9} \times \pi \times (0.1)^2}_{\text{H atom}} = 1.62 \times \pi \times (1.4)^2 + \frac{\pi}{9} \times (0.1)^2 = 9.98\ A^{\circ 2}$$

Planar density= $\frac{[Number\ of\ atoms\ in\ plane\ (211) \times area\ of\ each\ atom\ in\ the\ plane\ (211)]}{area\ of\ the\ plane\ (211)} = \frac{9.98}{47.14} = 0.212$

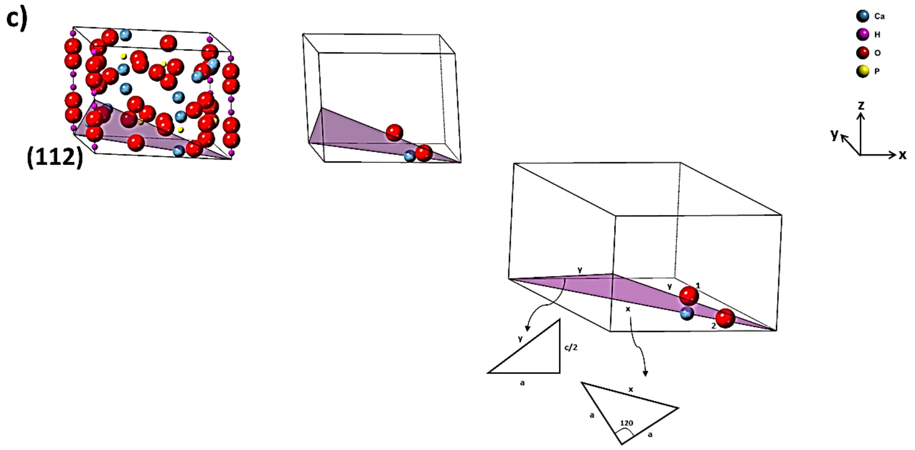

Cosine's rule: $x^2 = a^2 + a^2 - 2 \times a \times a \times cos120^{\circ} = 3a^2$

$$\rightarrow x = \sqrt{3}a = \sqrt{3} \times (9.4) = 16.281$$

$$y^2 = a^2 + \left(\frac{c}{2}\right)^2 = (9.4)^2 + \left(\frac{6.93}{2}\right)^2 = 100.366 \rightarrow y = 10.018$$

Area of the plane (112): $s \xrightarrow{Hero\,n's\ law} s = \sqrt{p(p-y)(p-y)(p-x)}$

$$p = \frac{x+y+y}{2} = \frac{16.281 + 2 \times (10.018)}{2} = 18.159$$

$$s = \sqrt{18.159 \times (18.159 - 10.018)^2 \times (18.159 - 16.281)} = 47.541\ A^{\circ 2}$$

Number of atoms in the plane (112)×area of each atom in the plane (112)

$$= \underbrace{\left(0.5 \times \frac{60}{67}\right)}_{\text{Ca atom}} \times \pi \times (1)^2 + \left[\underbrace{\frac{12}{51}}_{\text{O atom no.1}} + \underbrace{\left(\frac{43}{51} \times \frac{139}{148}\right)}_{\text{O atom no.2}}\right] \times \pi \times (1.4)^2 = 1.407 + 6.325 =$$

$$7.732\ A^{\circ 2}$$

$$\text{Planar density} = \frac{[Number\ of\ atoms\ in\ plane(112) \times area\ of\ each\ atom\ in\ the\ plane(112)]}{area\ of\ the\ plane\ (112)} = \frac{7.732}{47.541}$$

$$= 0.163$$

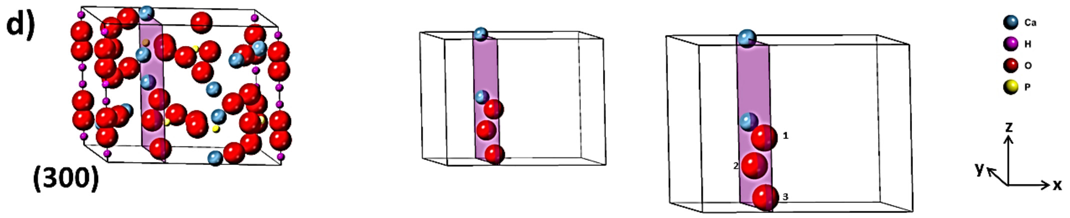

Area of the (300): $s = a \times c = 9.4 \times 6.93 = 65.142\ \text{A}^{\circ 2}$

Number of atoms in the plane (300)×area of each atom in the plane (300)

$$= \underbrace{\left(1 + \frac{1}{2}\right) \times \pi \times (1)^2}_{\text{Ca atom}} + \left[\underbrace{\frac{122}{139}}_{\text{O atom no.1}} + \underbrace{\frac{126}{139}}_{\text{O atom no.2}} + \underbrace{\left(\frac{122}{139} \times \frac{139}{148}\right)}_{\text{O atom no.3}}\right] \times \pi \times (1.4)^2 = 4.712 +$$

$$16.062 = 20.774\ A^{\circ 2}$$

$$\text{Planar density} = \frac{[Number\ of\ atoms\ in\ plane(300) * area\ of\ each\ atom\ in\ the\ plane(300)]}{area\ of\ the\ plane\ (300)} = \frac{20.774}{65.142}$$

$$= 0.319$$

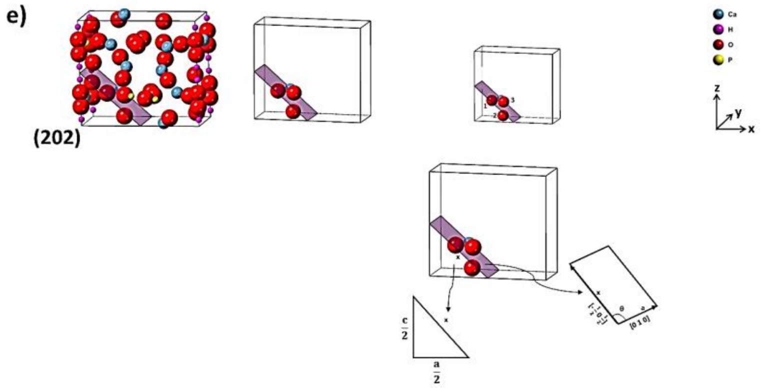

$$x^2 = (\frac{a}{2})^2 + (\frac{c}{2})^2 = \left(\frac{9.4}{2}\right)^2 + \left(\frac{6.93}{2}\right)^2 = 34.0962 \rightarrow x = 5.839$$

Angle between two miller hexagonal directions:

$$[u_1\ v_1\ w_1] < [u_2\ v_2\ w_2] \quad [0\ 1\ 0] \overset{?}{<} \left[-\frac{1}{2}\ 0\ \ \frac{1}{2}\right]$$

$$cos\theta = \frac{a^2\left[u_1u_2 + v_1v_2 - \frac{1}{2}(u_1v_2 + v_1u_2)\right] + c^2w_1w_2}{\sqrt{a^2(u_1^2 - u_1v_1 + v_1^2) + c^2w_1^2}\ \sqrt{a^2(u_2^2 - u_2v_2 + v_2^2) + c^2w_2^2}}$$

$$cos\theta = \frac{(9.4)^2\left[0 + 0 - \frac{1}{2}\left(0 - \frac{1}{2}\right)\right] + 0}{\sqrt{(9.4)^2(0 - 0 + 1^2) + (6.93)^2 \times 0}\ \sqrt{(9.4)^2\left(\left(\frac{-1}{2}\right)^2 + 0 + 0\right) + (6.93)^2\left(\frac{1}{2}\right)^2}}$$

$$cos\theta = \frac{(9.4)^2\left(\frac{1}{4}\right)}{\sqrt{(9.4)^2}\sqrt{(9.4)^2\left(\frac{1}{2}\right)^2 + (6.93)^2\left(\frac{1}{2}\right)^2}} = 0.4 \rightarrow \theta = 66.3°$$

Area of the plane (202): $S = a \times x \times \sin(66.3°) = (9.4) \times (5.839) \times \sin(66.3°) = 50.258 \mathring{A}^2$

Number of atoms in the plane (202)×area of each atom in the plane (202)

$$=\underbrace{\left(\frac{17}{19}\right) * 0.57 \times \pi \times (1)^2}_{\text{Ca atom}} + \left[\underbrace{\frac{4}{27}}_{\text{O atom no.1}} + \underbrace{\left(\frac{5}{27} \times \frac{139}{148}\right)}_{\text{O atom no.2}} + \underbrace{\left(\frac{5}{18}\right)}_{\text{O atom no.3}}\right] \times \pi \times (1.4)^2 = 1.6 +$$

$$3.694 = 5.294\ \mathring{A}^2$$

Planar density = $\frac{[Number\ \ of\ \ atoms\ \ in\ plane\ (202) \times area\ \ of\ \ each\ atom\ \ in\ the\ plane\ (202)]}{area\ \ of\ the\ plane\ \ (202)} = \frac{5.294}{50.258} = 0.105$

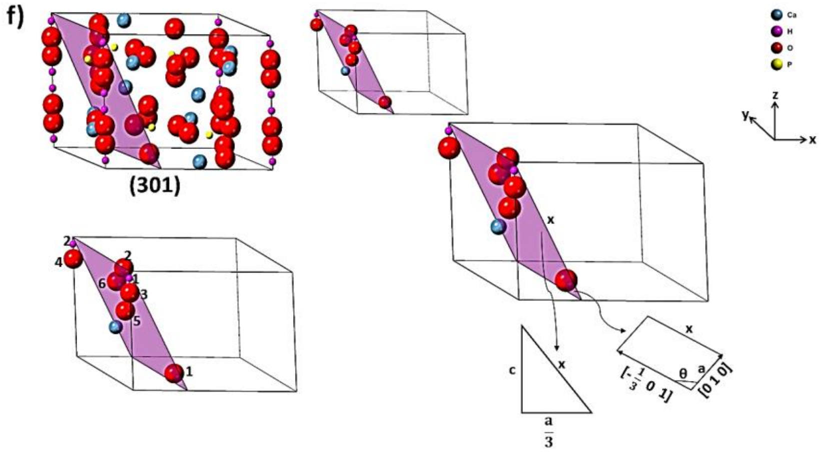

$$x^2 = \left(\frac{a}{3}\right)^2 + c^2 = \left(\frac{9.4}{3}\right)^2 + (6.93)^2 = 57.842$$

$$\rightarrow x = 7.605$$

Angle between two miller hexagonal directions:

$$[u_1\ v_1\ w_1] < [u_2\ v_2\ w_2] \quad \left[\frac{-1}{3}\ 0\ 1\right] \overset{?}{<} [0\ 1\ 0]$$

$$cos\theta = \frac{a^2\left[u_1u_2 + v_1v_2 - \frac{1}{2}(u_1v_2 + v_1u_2)\right] + c^2w_1w_2}{\sqrt{a^2(u_1^2 - u_1v_1 + v_1^2) + c^2w_1^2}\ \sqrt{a^2(u_2^2 - u_2v_2 + v_2^2) + c^2w_2^2}}$$

$$= \frac{(9.4)^2\left[0 + 0 - \frac{1}{2}\left(\frac{-1}{3} + 0\right)\right] + (6.93)^2(0)}{\sqrt{(9.4)^2\left(\left(\frac{-1}{3}\right)^2 - \left(\frac{-1}{3} \times 0\right) + 0\right) + (6.93)^2(1)^2}\ \sqrt{(9.4)^2(0 - 0 + 1) + (6.93)^2 \times 0}}$$

$$= \frac{(9.4)^2\left(\frac{+1}{6}\right)}{\sqrt{(9.4)^2\left(\frac{1}{9}\right) + (6.93)^2}\ \sqrt{(9.4)^2}} = 0.206 \rightarrow \theta = 78.112°$$

Area of the plane (301): $S = a \times x \times \sin 78.112° = 9.4 \times 7.605 \times 0.979 = 69.954 Å^2$

Number of atom in the plane (301) × area of each atom in the plane (301)

$$= \left[\left(\frac{64}{112} \times \frac{139}{148}\right) + \left(\frac{22}{112} \times \frac{139}{148}\right) + \left(\frac{20}{112} \times \frac{139}{148}\right) + \left(\frac{6}{112} \times \frac{9}{148}\right) + \left(\frac{24}{112}\right)\right.$$

O atom no.1, O atom no.2, O atom no.3, O atom no.4, O atom no.5

$$\left. + \left(\frac{60}{112}\right)\right] \times \pi \times (1.4)^2 + \left[\frac{64}{78} \times 0.56\right] \times \pi \times (1)^2$$

O atom no.6, Ca atom

$$+ \left[\left(\frac{10}{27} \times 0.5\right) + \left(\frac{4}{27} \times 0.5\right)\right] \times \pi \times (0.1)^2$$

H atom no.1, H atom no.2

$$= 10.111 + 1.44 + 0.008 = 11.559$$

Planar density $= \frac{[Number\ of\ atoms\ in\ plane\ (301) * area\ of\ each\ atom\ in\ the\ plane\ (301)]}{area\ of\ the\ plane\ (301)} = \frac{11.559}{69.954} = 0.165$

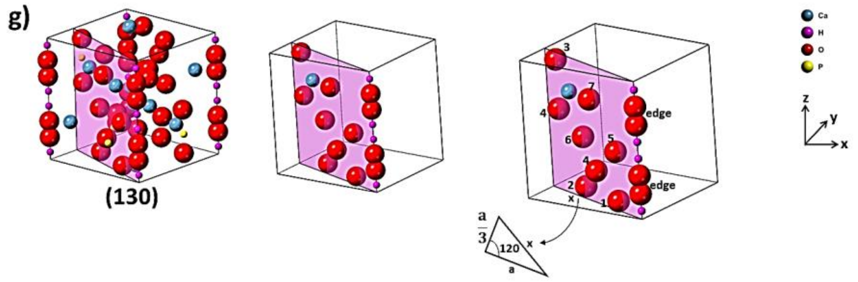

Cosine's rule: $x^2 = (a)^2 + \left(\frac{a}{3}\right)^2 - 2(a)\left(\frac{a}{3}\right)cos120^{\circ} = (9.4)^2 + \left(\frac{9.4}{3}\right)^2 - 2(9.4)\left(\frac{9.4}{3}\right)cos120^{\circ} = 127.63 \rightarrow x = 11.297$

Area of the plane (130):$s = x \times c = (11.297) \times (6.93) = 78.288A^{\circ 2}$

Number of atoms in the plane (130) ×area of each atom in the plane (130)

$$=\Big[\underbrace{\left(4\times\frac{1}{2}\right)}_{\text{O atom edge}} + \underbrace{\left(\frac{122}{125}\times\frac{139}{148}\right)}_{\text{O atom no.1}} + \underbrace{\left(\frac{68}{125}\times\frac{139}{148}\right)}_{\text{O atom no.2}} + \underbrace{\left(\frac{70}{125}\times\frac{139}{148}\right)}_{\text{O atom no.3}} + \underbrace{\left(\frac{70}{125}\times 2\right)}_{\text{O atom no.4}} + \underbrace{\left(\frac{122}{125}\right)}_{\text{O atom no.5}} +$$

$$\underbrace{\left(\frac{68}{125}\right)}_{\text{O atom no.6}} + \underbrace{\left(\frac{104}{125}\right)}_{\text{O atom no.7}}\Big] * \pi * (1.4)^2 + \underbrace{\left(\frac{8}{9}\right) * \pi * (1)^2}_{\text{Ca atom}} + \underbrace{\left[\left(4\times\frac{1}{2}\right)\right]\times\pi\times(0.1)^2}_{\text{H atoms}} =$$

$45.723 + 2.793 + 0.0628 = 48.579\ A^{\circ 2}$

Planar density=$\frac{[Number\ \ of\ atoms\ \ in\ plane\ (130)\times area\ \ of\ each\ atom\ in\ the\ plane\ (130)]}{area\ \ of\ the\ plane\ \ (130)} = \frac{48.579}{78.288} = 0.621$

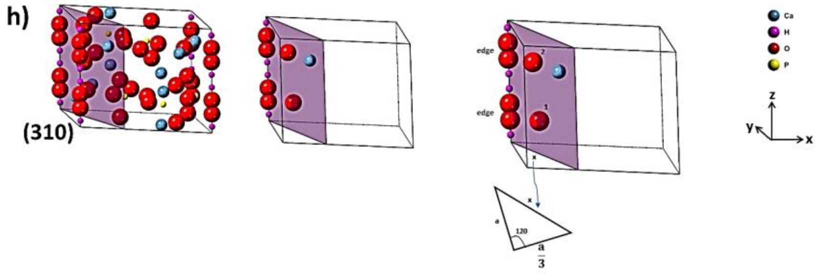

Cosine's rule: $x^2 = (a)^2 + \left(\frac{a}{3}\right)^2 - 2(a)\left(\frac{a}{3}\right)cos120^{\circ} = (9.4)^2 + \left(\frac{9.4}{3}\right)^2 - 2(9.4)\left(\frac{9.4}{3}\right)cos120° = 127.63 \rightarrow x = 11.297$

Area of the plane (310):$s = x \times c = 11.297 \times 6.93 = 78.288\ A^{\circ 2}$

Number of atoms in the plane (310) ×area of each atom in the plane (310)

$$=\underbrace{\left(4\times\frac{1}{2}\right)\times\pi\times(0.1)^2}_{\text{H atoms}} + \Big[\underbrace{\left(4\times\frac{1}{2}\right)}_{\text{O atom edge}} + \underbrace{\frac{52}{125}}_{\text{O atom no.1}} + \underbrace{\frac{18}{25}}_{\text{O atom no.2}}\Big]\times\pi\times(1.4)^2 + \underbrace{1\times\pi\times(1)^2}_{\text{Ca atom}} =$$

$0.0628 + 19.31 + 3.142 = 22.514\ \ A^{\circ 2}$

Planar density=$\frac{[Number\ \ of\ atoms\ \ in\ plane\ (310)*area\ \ of\ each\ atom\ in\ the\ plane\ (310)]}{area\ \ of\ the\ plane\ \ (310)} = \frac{22.514}{78.288} = 0.288$

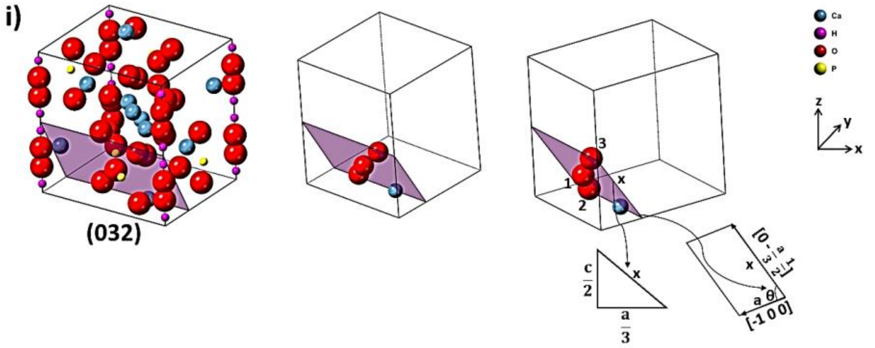

$$x^2 = \left(\frac{a}{3}\right)^2 + \left(\frac{c}{2}\right)^2 = \left(\frac{9.4}{3}\right)^2 + \left(\frac{6.93}{2}\right)^2 = 21.824 \rightarrow x = 4.672$$

Angle between two miller hexagonal directions:

$$[u_1\ v_1\ w_1] < [u_2\ v_2\ w_2] \quad [-1\ 0\ 0] < \left[0\ \frac{-1}{3}\ \frac{1}{2}\right]$$

$$cos\theta = \frac{a^2\left[u_1u_2 + v_1v_2 - \frac{1}{2}(u_1v_2 + v_1u_2)\right] + c^2w_1w_2}{\sqrt{a^2(u_1^2 - u_1v_1 + v_1^2) + c^2w_1^2}\ \sqrt{a^2(u_2^2 - u_2v_2 + v_2^2) + c^2w_2^2}}$$

$$= \frac{(9.4)^2\left[0 + 0 - \frac{1}{2}\left((-1)(\frac{-1}{3}) + 0\right)\right] + (6.93)^2 \times 0}{\sqrt{(9.4)^2((-1)^2 - 0 + 0) + 0}\ \sqrt{(9.4)^2\left(0 - 0 + \left(\frac{-1}{3}\right)^2\right) + (6.93)^2 \times \left(\frac{1}{2}\right)^2}}$$

$$= \frac{(9.4)^2\left[\frac{-1}{6}\right]}{(6.4)\sqrt{(9.4)^2\left(\frac{1}{9}\right) + (6.93)^2(\frac{1}{4})}} = -0.335 \rightarrow \theta = 109.573°$$

Area of the plane (032): $S = a \times x \times \sin 109.573° = (9.4) \times (4.672) \times \sin(109.573°) = 41.379\ \text{Å}^2$

Number of atoms in the plane (032)×area of each atom in the plane (032)

$$=\left[\underbrace{\left(\frac{46}{106}\right)}_{\text{O atom no.1}} + \underbrace{\left(\frac{72}{106} \times \frac{139}{148}\right)}_{\text{O atom no.2}} + \underbrace{\left(\frac{62}{106}\right)}_{\text{O atom no.3}}\right] \times \pi \times (1.4)^2 + \underbrace{\left[\frac{58}{69} \times 0.5 \times \pi \times (1)^2\right]}_{\text{Ca atom}} =$$

$10.202 + 1.32 = 11.522\ \text{Å}^2$

Planar density$=\frac{[Number\ of\ atoms\ in\ plane\ (032) \times area\ of\ each\ atom\ in\ the\ plane\ (032)]}{area\ of\ the\ plane\ (032)} = \frac{11.522}{41.579} = 0.278$

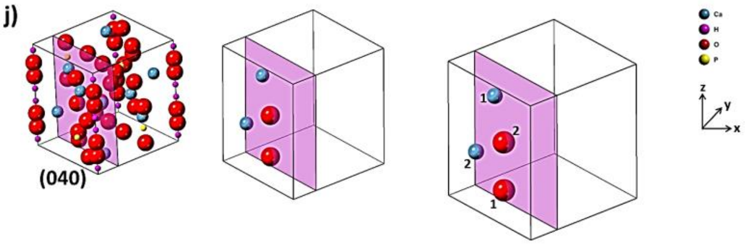

Area of the (040): s=a×c= (9.4)×(6.93) =65.142 $A^{\circ 2}$

Number of atoms in the plane (040)×area of each atom in the plane (040)

$$=\left[\underbrace{\left(\frac{122}{135}\times\frac{139}{148}\right)}_{\text{O atom no.1}}+\underbrace{\left(\frac{122}{138}\right)}_{\text{O atom no.2}}\right]\times\pi\times(1.4)^2+\left[\underbrace{\left(\frac{84}{100}\right)}_{\text{Ca atom no.1}}+\underbrace{\left(0.56\times\frac{92}{100}\right)}_{\text{Ca atom no.2}}\right]\times\pi\times(1)^2=$$

$$10.556+4.257=14.813\ A^{\circ 2}$$

Planar density= $\frac{[Number\ \ of\ atoms\ \ in\ plane\ (040)\times area\ \ of\ each\ atom\ \ in\ the\ plane\ (040)]}{area\ \ of\ the\ plane\ \ (040)}=\frac{14.813}{65.142}=0.227$

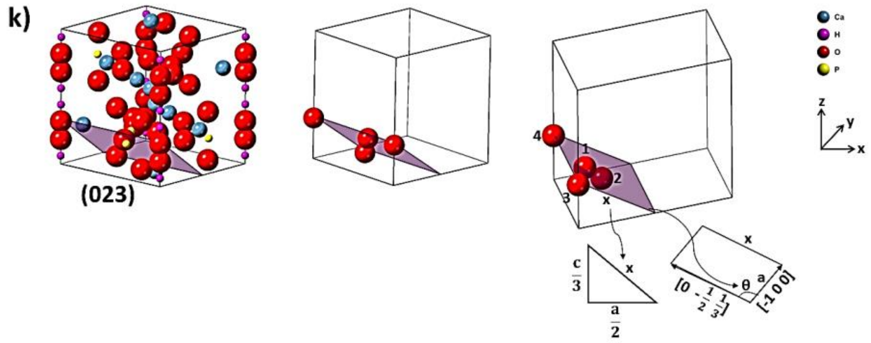

$$x^2=\left(\frac{a}{2}\right)^2+\left(\frac{c}{3}\right)^2=\left(\frac{9.4}{2}\right)^2+\left(\frac{6.93}{3}\right)^2=27.426\rightarrow x=5.237$$

Angle between two miller hexagonal directions:

$$[u_1\ v_1\ w_1]<[u_2\ v_2\ w_2]\quad[-1\ 0\ 0]\stackrel{?}{<}\left[0\ \ \frac{-1}{2}\ \ \frac{1}{3}\right]$$

$$cos\theta=\frac{a^2\left[u_1u_2+v_1v_2-\frac{1}{2}(u_1v_2+v_1u_2)\right]+c^2w_1w_2}{\sqrt{a^2(u_1^2-u_1v_1+v_1^2)+c^2w_1^2}\sqrt{a^2(u_2^2-u_2v_2+v_2^2)+c^2w_2^2}}$$

$$=\frac{(9.4)^2\left[0+0-\frac{1}{2}\left((-1)(\frac{-1}{2})+0\right)\right]+(6.93)^2\times 0}{\sqrt{(9.4)^2((-1)^2-0+0)+0}\sqrt{(9.4)^2\left(0-0+(\frac{-1}{2})\right)+(6.93)^2\left(\frac{1}{3}\right)^2}}$$

$$=\frac{(9.4)^2\left[\frac{-1}{4}\right]}{(9.4)\sqrt{(9.4)^2\left(\frac{1}{4}\right)+(6.93)^2\times\frac{1}{9}}}=-0.449\rightarrow\theta=116.662°$$

Area of the plane (023): $S=a\times x\times\sin\theta=(9.4)\times(5.237)\times\sin(116.662°)=$ 43.993 $A^{\circ 2}$

Number of atoms in the plane (023)×area of each atom in the plane (023)

$$=\left[\underbrace{\left(\frac{106}{112}\right)}_{\text{O atom no.1}}+\underbrace{\left(\frac{4}{112}\times\frac{139}{148}\right)}_{\text{O atom no.2}}+\underbrace{\left(\frac{63.338}{360}\times\frac{92}{112}\right)}_{\text{O atom no.3}}+\underbrace{\left(\frac{116.662}{360}\times\frac{92}{112}\right)}_{\text{O atom no.4}}\right]\times\pi\times(1.4)^2=$$

$$8.524\ A^{\circ 2}$$

Planar density= $\frac{[Number\ \ of\ atoms\ \ in\ plane\ (023)\times area\ \ of\ each\ atom\ \ in\ the\ plane\ (023)]}{area\ \ of\ the\ plane\ \ (023)}=\frac{8.524}{43.993}=0.194$

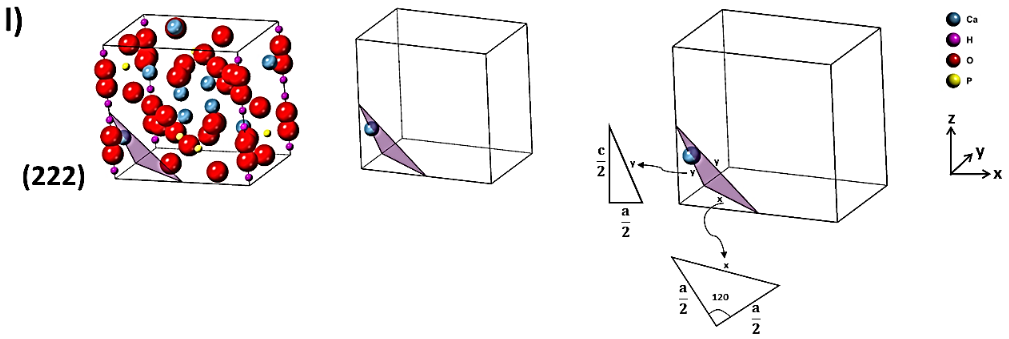

Cosine's rule: $x^2 = \left(\frac{a}{2}\right)^2 + \left(\frac{a}{2}\right)^2 - 2\left(\frac{a}{2}\right)\left(\frac{a}{2}\right) cos120^{\circ} = \frac{a^2}{2} + \frac{a^2}{4} = \frac{3}{4}a^2$

$$\rightarrow x = \frac{\sqrt{3}}{2}a = \frac{\sqrt{3}}{2} \times (9.4) = 8.141$$

$y^2 = \left(\frac{a}{2}\right)^2 + \left(\frac{c}{2}\right)^2 = \left(\frac{9.4}{2}\right)^2 + \left(\frac{6.93}{2}\right)^2 = 34.0962 \rightarrow y = 5.839$

Area of the plane (222):$s \xrightarrow{Hero\,n's\,law} s = \sqrt{p(p-y)(p-y)(p-x)}$

$p = \frac{x+y+y}{2} = \frac{2 \times (5.839) + 8.141}{2} = 9.91$

$s = \sqrt{(9.91)(9.91-5.839)^2(9.91-8.141)} = 17.045\ A^{\circ 2}$

Number of atoms in the plane (222) ×area of each atom in the plane (222) =

$$0.57 \times \left(\frac{29}{34}\right) \times \pi \times (1)^2 = 1.527\ A^{\circ 2}$$

Planar density =

$= \frac{[Number\ of\ atoms\ in\ plane(222) \times area\ of\ each\ atom\ in\ the\ plane(222)]}{area\ of\ the\ plane\ (222)}$

$= \frac{1.527}{17.045} = 0.0896$

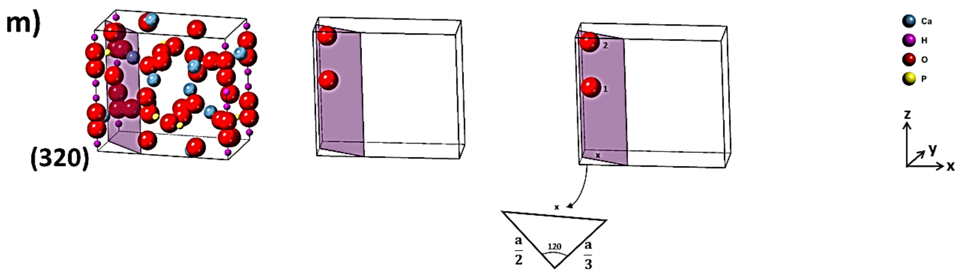

Cosine's rule: $x^2 = \left(\frac{a}{2}\right)^2 + \left(\frac{a}{3}\right)^2 - 2\left(\frac{a}{2}\right)\left(\frac{a}{3}\right)cos120^{\circ} = \left(\frac{9.4}{2}\right)^2 + \left(\frac{9.4}{3}\right)^2 - 2\left(\frac{9.4}{2}\right)\left(\frac{9.4}{3}\right)cos120^{\circ} = 46.634 \rightarrow x = 6.829$

Area of the plane (320):$s = x \times c = 6.829 \times 6.93 = 47.325\ A^{\circ 2}$

Number of atoms in the plane (320)×area of each atom in the plane (320)

$=\left[\left(\frac{47}{60}\right) + \left(\frac{47}{60} \times \frac{139}{148}\right)\right] \times \pi \times (1.4)^2 = 9.353 A^{\circ 2}$

O atom no.1 O atom no.2

Planar density=$\frac{[Number\ \ of\ \ atoms\ \ in\ plane\ (320) \times area\ \ of\ \ each\ atom\ \ in\ the\ plane\ (320)]}{area\ \ of\ the\ plane\ \ (320)} = \frac{9.353}{47.325} = 0.198$

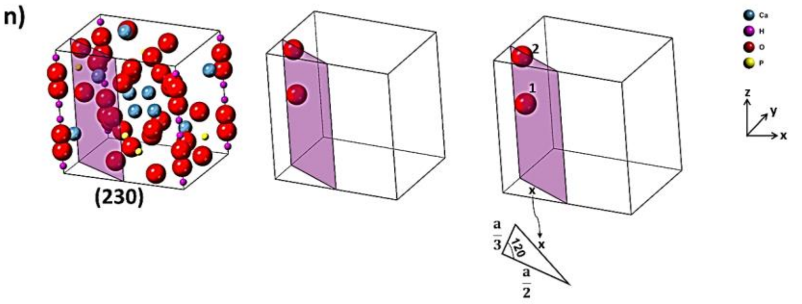

Cosine's rule: $x^2 = \left(\frac{a}{2}\right)^2 + \left(\frac{a}{3}\right)^2 - 2\left(\frac{a}{2}\right)\left(\frac{a}{3}\right)cos120^{\circ} = \left(\frac{9.4}{2}\right)^2 + \left(\frac{9.4}{3}\right)^2 - 2\left(\frac{9.4}{2}\right)\left(\frac{9.4}{3}\right)cos120^{\circ} = 46.634 \rightarrow x = 6.289$

Area of the plane (230):$s = x \times c = (6.289) \times 6.93 = 47.325\ A^{\circ 2}$

Number of atoms in the plane (230)×area of each atom in the plane (230)

$=\left[\left(\frac{44}{120}\right) + \left(\frac{44}{120} \times \frac{139}{148}\right)\right] \times \pi \times (1.4)^2 = 4.378\ A^{\circ 2}$

O atom no.1 O atom no.2

Planar density=$\frac{[Number\ \ of\ \ atoms\ \ in\ plane\ (230) \times area\ \ of\ \ each\ atom\ \ in\ the\ plane\ (230)]}{area\ \ of\ the\ plane\ \ (230)} = \frac{4.378}{47.325} = 0.093$

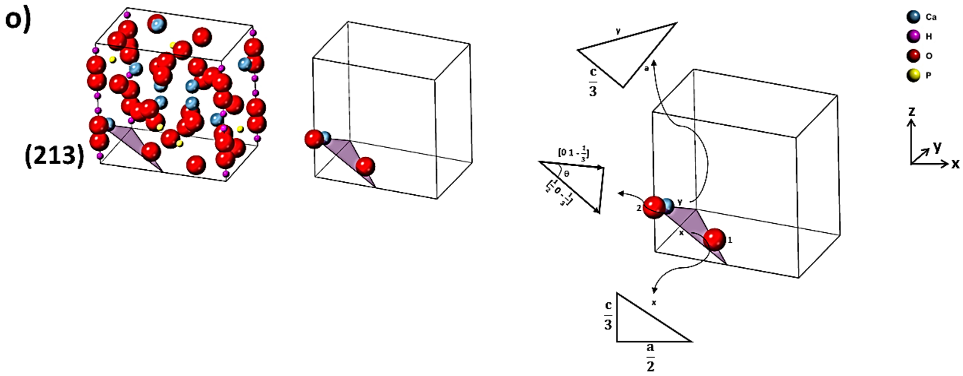

Angle between two miller hexagonal directions:

$[u_1\ v_1\ w_1] < [u_2\ v_2\ w_2] \quad \left[\frac{+1}{2}\ 0\ \frac{-1}{3}\right] < \left[0\ 1\ \frac{-1}{3}\right]$

$$cos\theta = \frac{a^2\left[u_1u_2 + v_1v_2 - \frac{1}{2}(u_1v_2 + v_1u_2)\right] + c^2w_1w_2}{\sqrt{a^2(u_1^2 - u_1v_1 + v_1^2) + c^2w_1^2}\sqrt{a^2(u_2^2 - u_2v_2 + v_2^2) + c^2w_2^2}}$$

$$= \frac{(9.4)^2\left[0 + 0 - \frac{1}{2}\left(\frac{1}{2}(1) + 0\right)\right] + (6.93)^2 \times (\frac{-1}{3})}{\sqrt{(9.4)^2\left(\left(\frac{1}{2}\right)^2 - 0 + 0\right) + (6.93)^2\left(\frac{-1}{3}\right)^2}\ \sqrt{(9.4)^2(0 - 0 + 1^2) + (6.93)^2 \times \left(\frac{-1}{3}\right)^2}}$$

$$= \frac{-16.754}{9.679 \times 5.237} = -0.33 \rightarrow \theta = 109.3°$$

$$x^2 = \left(\frac{a}{2}\right)^2 + \left(\frac{c}{3}\right)^2 = \left(\frac{9.4}{2}\right)^2 + \left(\frac{6.93}{3}\right)^2 = 27.426 \rightarrow x = 5.237$$

$$y^2 = (a)^2 + \left(\frac{c}{3}\right)^2 = (9.4)^2 + \left(\frac{6.93}{3}\right)^2 = 93.696 \rightarrow y = 9.68$$

Area of the plane (213): S $= y \times x \times 0.5 \times \sin(109.3°) = (9.68 \times 5.237) \times 0.5 \times 0.944 = 23.923\ \text{Å}^2$

Number of atoms in the plane (213)×area of each atom in the plane (213)

$$= \underbrace{0.56 \times \left(\frac{58}{69}\right) \times \pi \times (1)^2}_{\text{Ca atom}} + \left[\underbrace{\left(\frac{86}{93} \times \frac{109.3}{360}\right)}_{\text{O atom no.2}} + \underbrace{\left(\frac{25}{61} \times \frac{139}{148}\right)}_{\text{O atom no.1}}\right] \times \pi \times (1.4)^2 = 1.479 +$$

$$[0.281 + (0.385)] \times \pi \times (1.4)^2 = 5.58\ Å^2$$

Planar density $= \frac{[Number\ \ of\ atoms\ \ in\ plane\ (213) \times area\ \ of\ \ each\ atom\ in\ the\ plane\ (213)]}{area\ \ of\ \ the\ plane\ \ (213)} = \frac{5.58}{23.923} = 0.233$

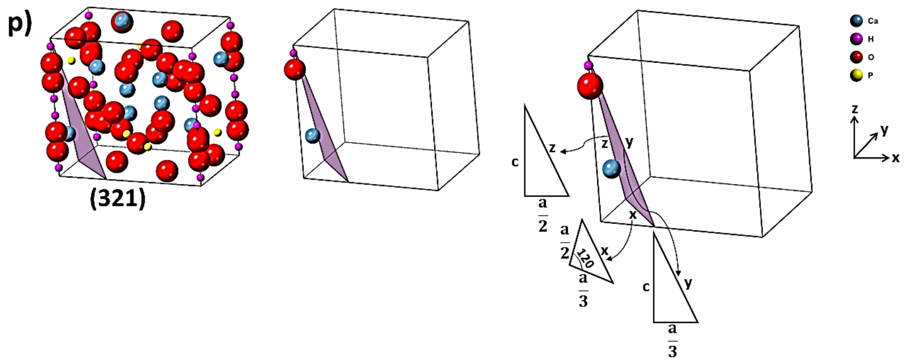

Cosine's rule: $x^2 = \left(\frac{a}{3}\right)^2 + \left(\frac{a}{2}\right)^2 - 2\left(\frac{a}{3}\right)\left(\frac{a}{2}\right)cos120^{\circ} = \left(\frac{9.4}{3}\right)^2 + \left(\frac{9.4}{2}\right)^2 - 2\left(\frac{9.4}{3}\right)\left(\frac{9.4}{2}\right)cos120^{\circ} = 46.634 \rightarrow x = 6.829$

$$y^2 = \left(\frac{a}{3}\right)^2 + c^2 = \left(\frac{9.4}{3}\right)^2 + (6.93)^2 = 57.843 \rightarrow y = 7.605$$

$$Z^2 = (\frac{a}{2})^2 + c^2 = \left(\frac{9.4}{2}\right)^2 + (6.93)^2 = 70.115 \rightarrow z = 8.373$$

Area of the plane (321): $s \xrightarrow{Heron's\ law} s = \sqrt{p(p-x)(p-y)(p-z)}$

$$p = \frac{x+y+y}{2} = \frac{(6.829) + (7.605) + 8.373}{2} \quad p = 11.404$$

$$s = \sqrt{11.404(11.404 - 6.829)(11.404 - 7.605)(11.404 - 8.373)}$$

$$s = 24.510\ A^{\circ 2}$$

Number of atoms in the plane (321) ×area of each atom in the plane (321)

$$= \underbrace{\left(\frac{44}{82} \times \frac{139}{148}\right) \times \pi \times (1.4)^2}_{\text{O atom}} + \underbrace{\left(\frac{2}{61}\right) \times \pi \times (1)^2}_{\text{Ca atom}} + \underbrace{\left(\frac{18}{24} \times \pi \times (0.1)^2\right)}_{\text{H atom}} = 3.304 + 0.103 + 0.0235 = 3.431\ A^{\circ 2}$$

Planar density $= \frac{[Number\ of\ atoms\ in\ plane\ (321) \times area\ of\ each\ atom\ in\ the\ plane\ (321)]}{area\ of\ the\ plane\ (321)} = \frac{3.341}{24.510} = 0.140$

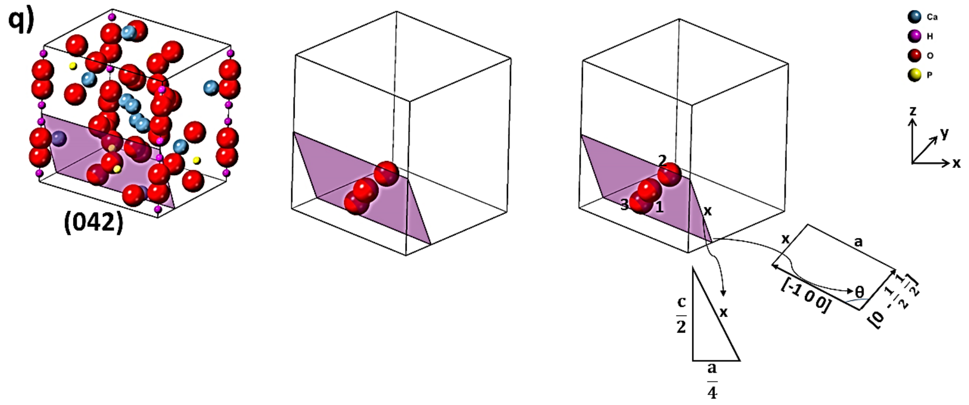

$$x^2 = \left(\frac{a}{4}\right)^2 + \left(\frac{c}{2}\right)^2 = \left(\frac{9.4}{2}\right)^2 + \left(\frac{6.93}{2}\right)^2 = 17.529 \rightarrow x = 4.187$$

Angle between two miller hexagonal directions:

$$[u_1\, v_1\, w_1] < [u_2\, v_2\, w_2] \quad [-1\;0\;0] \lessgtr \left[0\; \frac{-1}{4}\; \frac{1}{2}\right]$$

$$cos\theta = \frac{a^2\left[u_1u_2 + v_1v_2 - \frac{1}{2}(u_1v_2 + v_1u_2)\right] + c^2w_1w_2}{\sqrt{a^2(u_1^2 - u_1v_1 + v_1^2) + c^2w_1^2}\,\sqrt{a^2(u_2^2 - u_2v_2 + v_2^2) + c^2w_2^2}}$$

$$cos\theta = \frac{(9.4)^2\left[0 + 0 - \frac{1}{2}\left((-1)\left(\frac{-1}{4}\right) + 0\right)\right] + 0}{\sqrt{(9.4)^2((-1)^2 - 0 + 0) + 0}\,\sqrt{(9.4)^2\left(0 - 0 + \left(\frac{-1}{4}\right)^2\right) + (6.93)^2\left(\frac{1}{2}\right)^2}}$$

$$cos\theta = \frac{(9.4)^2\left(\frac{-1}{8}\right)}{\sqrt{(9.4)^2}\sqrt{(9.4)^2\left(\frac{1}{16}\right) + (6.93)^2 \times \frac{1}{4}}} = -0.281 \rightarrow \theta = 106.299°$$

Area of the plane (032): $S = a \times x \times \sin\theta = (9.4) \times (4.187) \times \sin(106.299°)$

$$s = 37.776\ \mathring{A}^2$$

Number of atoms in the plane (042)×area of each atom in the plane (042)

$$= \Big[\underbrace{\left(\frac{98}{108}\right)}_{\text{O atom no.1}} + \underbrace{\left(\frac{42}{108}\right)}_{\text{O atom no.2}} + \underbrace{\left(\frac{50}{108} \times \frac{139}{148}\right)}_{\text{O atom no.3}}\Big] \times \pi \times (1.4)^2 = 10.659\ \mathring{A}^2$$

Planar density $= \frac{[Number\ of\ atoms\ in\ plane\ (042) \times area\ of\ each\ atom\ in\ the\ plane\ (042)]}{area\ of\ the\ plane\ (042)} = \frac{10.659}{37.776} = 0.282$

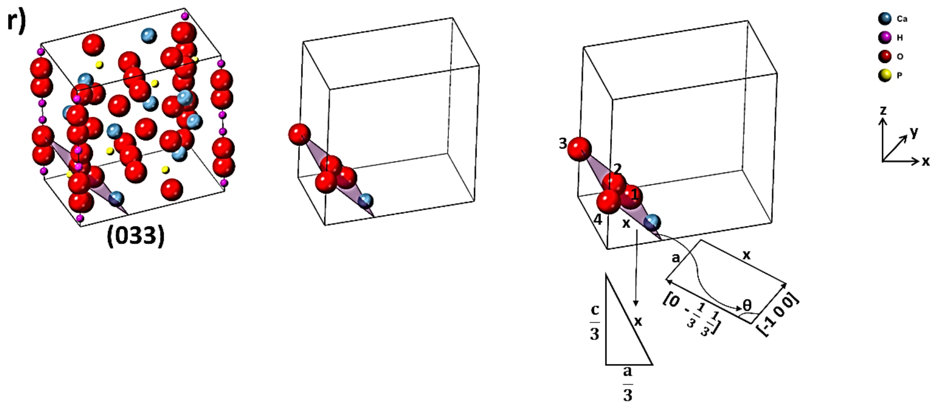

$$x^2 = \left(\frac{a}{3}\right)^2 + \left(\frac{c}{3}\right)^2 = \left(\frac{9.4}{3}\right)^2 + \left(\frac{6.93}{3}\right)^2 = 15.154 \rightarrow x = 3.893$$

Angle between two miller hexagonal directions:

$$[u_1\ v_1\ w_1] < [u_2\ v_2\ w_2] \quad [-1\ 0\ 0] \angle \left[0\ \frac{-1}{3}\ \frac{1}{3}\right]$$

$$cos\theta = \frac{a^2\left[u_1u_2 + v_1v_2 - \frac{1}{2}(u_1v_2 + v_1u_2)\right] + c^2w_1w_2}{\sqrt{a^2(u_1^2 - u_1v_1 + v_1^2) + c^2w_1^2}\ \sqrt{a^2(u_2^2 - u_2v_2 + v_2^2) + c^2w_2^2}}$$

$$cos\theta = \frac{(9.4)^2\left[0 + 0 - \frac{1}{2}\left((-1)\left(\frac{-1}{3}\right) + 0\right)\right] + (6.93)^2 \times (0)}{\sqrt{(9.4)^2((-1)^2 - 0 + 0) + (6.93)^2(0)^2}\ \sqrt{(9.4)^2\left(0 - 0 + \left(\frac{-1}{3}\right)^2\right) + (6.93)^2 \times \left(\frac{1}{3}\right)^2}}$$

$$cos\theta = \frac{(9.4)^2\left(\frac{-1}{6}\right)}{\sqrt{(6.4)^2}\sqrt{(9.4)^2\left(\frac{1}{9}\right) + (6.93)^2\left(\frac{1}{4}\right)}} = -0.402 \rightarrow \theta = 113.732°$$

Area of the plane (033): $S = a \times x \times sin\theta = (9.4) \times (3.893) \times \sin(113.732°) = 33.5\ A^{\circ 2}$

Number of atoms in the plane (033)×area of each atom in the plane (033)

$$= \Big[\underbrace{\left(\frac{102}{107} \times \frac{139}{148}\right)}_{\text{O atom no.1}} + \underbrace{\left(\frac{74}{107}\right)}_{\text{O atom no.2}} + \underbrace{\left(\frac{92}{107} \times \frac{113.732}{360}\right)}_{\text{O atom no.3}} + \underbrace{\left(\frac{92}{107} \times \frac{66.268}{360}\right)}_{\text{O atom no.4}}\Big] \times \pi \times (1.4)^2 +$$

$$\underbrace{\left[0.5 \times \frac{74}{76} \times \pi \times (1)^2\right]}_{\text{Ca atom}} = 12.418 + 1.529 = 13.947\ A^{\circ 2}$$

$$\text{Planar density} = \frac{[Number\ \ of\ \ atoms\ \ in\ \ plane\ (033) \times area\ \ of\ \ each\ atom\ \ in\ the\ plane\ (033)]}{area\ \ of\ the\ plane\ \ (033)} = \frac{13.947}{33.5} = 0.416$$

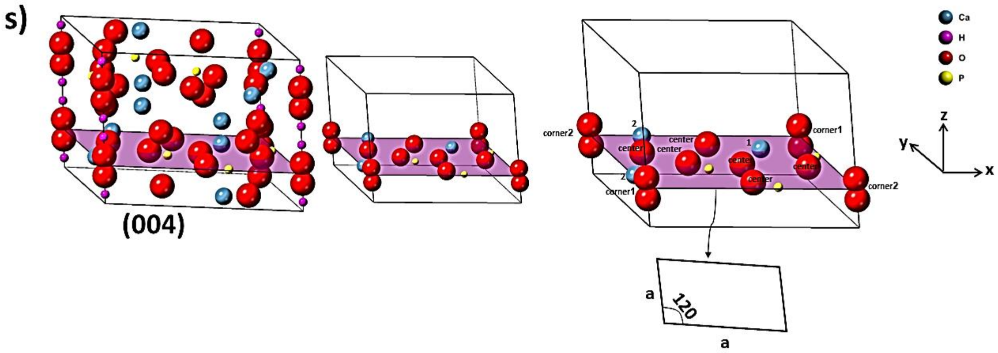

Area of the (004): s=a×a×sin120° = (9.4)×(9.4)×sin120° =76.522 $A^{\circ 2}$

Number of atoms in the plane (004)×area of each atom in the plane (004)

$$=\left[\underbrace{\left(4\times\frac{120}{360}\times\frac{16}{82}\right)}_{\text{O atom Corner 1}}+\underbrace{\left(4\times\frac{60}{360}\times\frac{10}{82}\right)}_{\text{O atom Corner 2}}+\underbrace{(6)}_{\text{O atom Center}}\right]\times\pi\times(1.4)^2+[\underbrace{(1)}_{\text{Ca atom no.1}}+\underbrace{(2\times 0.56)}_{\text{Ca atom no.2}}]\times$$

$$\pi\times(1)^2+\underbrace{[3\times\pi\times(0.31)]}_{\text{P atom}}=39.348+6.66+0.906=46.914\ A^{\circ 2}$$

$$\text{Planar density}=\frac{[Number\ \ of\ atoms\ \ in\ plane\ (004)\times area\ \ of\ \ each\ atom\ \ in\ the\ plane\ (004)]}{area\ \ of\ \ the\ plane\ \ (004)}=$$

$$\frac{46.914}{76.522}=0.613$$

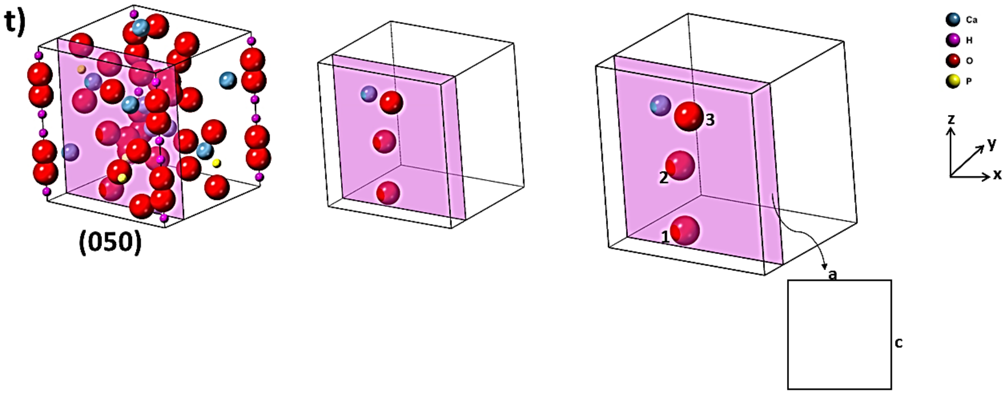

Area of the (050): s=a×c= (9.4)×(6.93) =65.142 $A^{\circ 2}$

Number of atoms in the plane (050)×area of each atom in the plane (050)

$$=\left[\underbrace{\left(\frac{22}{138}\times\frac{139}{148}\right)}_{\text{O atom no.1}}+\underbrace{\left(\frac{22}{138}\right)}_{\text{O atom no.2}}+\underbrace{\left(\frac{54}{138}\right)}_{\text{O atom no.3}}\right]\times\pi\times(1.4)^2+\underbrace{\left(\frac{14}{99}\right)\times\pi\times(1)^2}_{\text{Ca atom}}=4.313+$$

$$0.444=4.757\ A^{\circ 2}$$

$$\text{Planar density}=\frac{[Number\ \ of\ atoms\ \ in\ plane\ (050)\times area\ \ of\ \ each\ atom\ \ in\ the\ plane\ (050)]}{area\ \ of\ \ the\ plane\ \ (050)}=$$

$$\frac{4.757}{65.142}=0.073$$

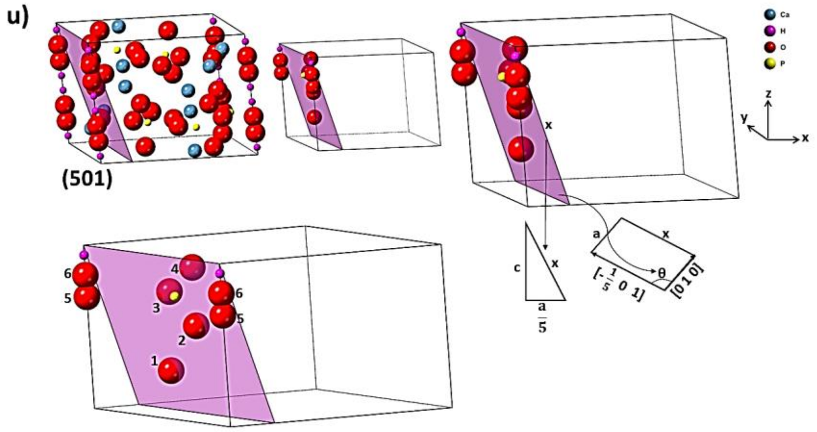

$$x^2 = \left(\frac{a}{5}\right)^2 + (c)^2 = \left(\frac{9.4}{5}\right)^2 + (6.93)^2 = 51.559 \rightarrow x = 7.180$$

Angle between two miller hexagonal directions:

$[u_1\, v_1\, w_1] < [u_2\, v_2\, w_2]$ $[0\,1\,0] \measuredangle \left[\frac{-1}{5}\;0\;\;1\right]$

$$cos\theta = \frac{a^2\left[u_1u_2 + v_1v_2 - \frac{1}{2}(u_1v_2 + v_1u_2)\right] + c^2w_1w_2}{\sqrt{a^2(u_1^2 - u_1v_1 + v_1^2) + c^2w_1^2}\,\sqrt{a^2(u_2^2 - u_2v_2 + v_2^2) + c^2w_2^2}}$$

$$= \frac{(9.4)^2\left[0 + 0 - \frac{1}{2}\left(0 + ((1) \times (\frac{-1}{5}))\right)\right] + (6.93)^2 \times 0}{\sqrt{(9.4)^2(0 - 0 + 1^2) + (6.93)^2 \times 0}\,\sqrt{(9.4)^2\left(\left(\frac{-1}{5}\right)^2 + 0 + 0\right) + (6.93)^2 \times 1^2}}$$

$$= \frac{(9.4)^2\left(\frac{1}{10}\right)}{(9.4)\sqrt{(9.4)^2\left(\frac{1}{25}\right) + (6.93)^2}} = 0.131 \rightarrow \theta = 82.473°$$

Area of the plane (501): $S = a \times (x) \times \sin\theta = (9.4) \times (7.18) \times \sin(82.473°) = 66.910\ \mathring{A}^2$

Number of atoms in the plane (501) ×area of each atom in the plane (501)

$$= \left[\underbrace{\left(\frac{106}{119}\right)}_{\text{O atom no.1}} + \underbrace{\left(\frac{116}{119}\right)}_{\text{O atom no.2}} + \underbrace{\left(\frac{6}{119}\right)}_{\text{O atom no.3}} + \underbrace{\left(\frac{2}{119} \times \frac{139}{148}\right)}_{\text{O atom no.4}} + \underbrace{\left(2 \times \frac{16}{119} \times 0.5\right)}_{\text{O atom no.5}} + \underbrace{\left(2 \times \frac{30}{119} \times 0.5\right)}_{\text{O atom no.6}}\right] \times$$

$$\pi \times (1.4)^2 + \underbrace{\left(\frac{8}{43}\right) * \pi \times (0.31)^2}_{\text{P atom}} + \underbrace{\left[0.5 \times \frac{22}{28} \times 2 \times \pi \times (0.1)^2\right]}_{\text{H atom}} = 14.275 +$$

$$0.0562 + 0.0247 = 14.356\ \mathring{A}^2$$

Planar density$= \frac{[Number\ \ of\ \ atoms\ \ in\ plane\ (501) \times area\ \ of\ \ each\ atom\ \ in\ the\ plane\ (501)]}{area\ \ of\ \ the\ plane\ \ (501)} =$

$$\frac{14.356}{66.910} = 0.213$$

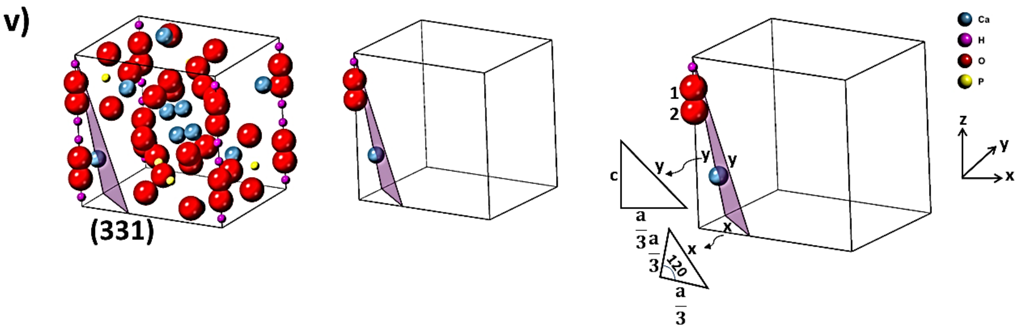

Cosine's law: $x^2 = \left(\frac{a}{3}\right)^2 + \left(\frac{a}{3}\right)^2 - 2\left(\frac{a}{3}\right)\left(\frac{a}{3}\right)cos120^{\circ} = \left(\frac{9.4}{3}\right)^2 + \left(\frac{9.4}{3}\right)^2 - 2 \times \left(\frac{9.4}{3}\right) \times \left(\frac{9.4}{3}\right) \times (-0.5) = 29.453 \rightarrow x = 5.427$

$$y^2 = \left(\frac{a}{3}\right)^2 + c^2 = \left(\frac{9.4}{3}\right)^2 + (6.93)^2 = 57.843 \rightarrow y = 7.605$$

Area of the plane (331):$s \xrightarrow{Hero\,n's\ law} s = \sqrt{p(p-x)(p-y)(p-y)}$

$$p = \frac{x+y+y}{2} = \frac{5.427 + (2 \times 7.605)}{2} = 10.319 \rightarrow$$

$$s = \sqrt{10.319(10.319 - 5.427) \times (10.319 - 7.605) \times (10.319 - 7.605)} = 19.283\ A^{\circ 2}$$

Number of atoms in the plane (331) ×area of each atom in the plane (331)

$$=\Big[\underbrace{\left(0.5 \times \frac{56}{108}\right)}_{\text{O atom no.1}} + \underbrace{\left(0.5 \times \frac{16}{108}\right)}_{\text{O atom no.2}}\Big] \times \pi \times (1.4)^2 + \underbrace{\left(0.56 \times \frac{34}{40}\right) \times \pi \times (1)^2}_{\text{Ca atom}} + \underbrace{\left(0.5 \times \frac{10}{19}\right) \times \pi \times (0.1)^2}_{\text{H atom}} = 2.053 + 1.495 + 0.0083 = 3.556\ A^{\circ 2}$$

$$\text{Planar density} = \frac{[Number\ of\ atoms\ in\ plane\ (331) \times area\ of\ each\ atom\ in\ the\ plane\ (331)]}{area\ of\ the\ plane\ (331)} = \frac{3.556}{19.283} = 0.184$$

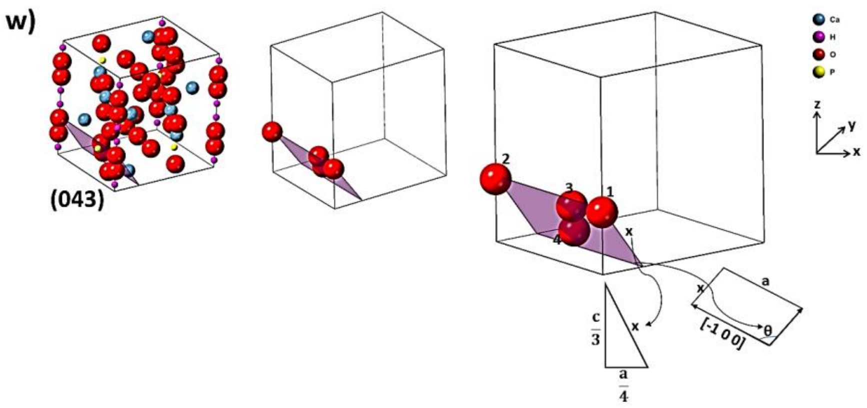

$$x^2 = \left(\frac{a}{4}\right)^2 + \left(\frac{c}{3}\right)^2 = \left(\frac{9.4}{4}\right)^2 + \left(\frac{6.93}{3}\right)^2 = 10.859 \rightarrow x = 3.295$$

Angle between two miller hexagonal directions:

$$[u_1\ v_1\ w_1] < [u_2\ v_2\ w_2] \quad [-1\ 0\ 0] \stackrel{?}{<} \left[0\ \frac{-1}{4}\ \frac{1}{3}\right]$$

$$cos\theta = \frac{a^2\left[u_1u_2 + v_1v_2 - \frac{1}{2}(u_1v_2 + v_1u_2)\right] + c^2w_1w_2}{\sqrt{a^2(u_1^2 - u_1v_1 + v_1^2) + c^2w_1^2}\ \sqrt{a^2(u_2^2 - u_2v_2 + v_2^2) + c^2w_2^2}}$$

$$cos\theta = \frac{(9.4)^2\left[0 + 0 - \frac{1}{2}\left((-1)\left(\frac{-1}{4}\right) + 0\right)\right] + (0)}{\sqrt{(9.4)^2((-1)^2 - 0 + 0) + 0}\ \sqrt{(9.4)^2\left(0 - 0 + \left(\frac{-1}{4}\right)^2\right) + (6.93)^2 \times \left(\frac{1}{3}\right)^2}}$$

$$cos\theta = \frac{(9.4)^2\left(\frac{-1}{8}\right)}{(9.4)\sqrt{(9.4)^2\left(\frac{1}{16}\right) + (6.93)^2\left(\frac{1}{9}\right)}} = -0.357 \rightarrow \theta = 110.9°$$

Area of the plane (043): $\mathrm{S} = a \times \mathrm{x} \times \sin\theta = (9.4) \times (3.295) \times \sin(110.9°)$

$$\mathrm{s} = 28.935\ \mathrm{A}^{\circ 2}$$

Number of atoms in the plane (043) ×area of each atom in the plane (043)

$$= \left[\left(\frac{92}{106} \times 0.5 \times \frac{110.9}{360}\right) + \left(\frac{92}{106} \times 0.5 \times \frac{69.1}{360}\right) + \left(\frac{44}{106}\right) + \left(\frac{36}{106} \times \frac{139}{148}\right)\right] \times \pi \times (1.4)^2 =$$

O atom no.1   O atom no.2   O atom no.3   O atom no.4

$5.856\ A^{\circ 2}$

Planar density= $\frac{[Number\ \ of\ atoms\ \ in\ plane\ (043) \times area\ \ of\ each\ atom\ \ in\ the\ plane\ (043)]}{area\ \ of\ the\ plane\ \ (043)} = \frac{5.856}{28.935} = 0.202$

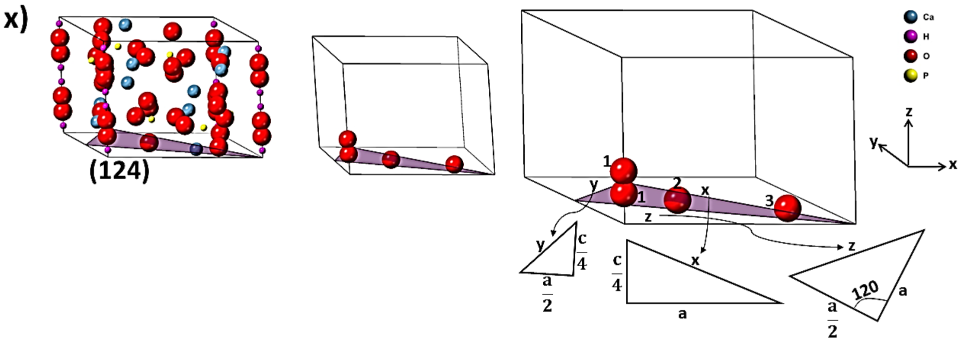

$$x^2 = \left(\frac{c}{4}\right)^2 + (a)^2 = \left(\frac{6.93}{4}\right)^2 + (9.4)^2 = 91.362 \rightarrow x = 9.558$$

$$y^2 = \left(\frac{c}{4}\right)^2 + \left(\frac{a}{2}\right)^2 = \left(\frac{6.93}{4}\right)^2 + (9.4/2)^2 = 25.092 \rightarrow y = 5.009$$

Cosine's rule: $z^2 = a^2 + \left(\frac{a}{2}\right)^2 - 2(a)\left(\frac{a}{2}\right)cos120° = (9.4)^2 + \left(\frac{9.4}{2}\right)^2 - 2(9.4)\left(\frac{9.4}{2}\right)cos120 = 154.63 \rightarrow z = 12.435$

Area of the plane (124): $s \xrightarrow{Heron's\ law} s = \sqrt{p(p-x)(p-y)(p-z)}$

$$p = \frac{x+y+y}{2} = \frac{9.558+5.009+12.435}{2} = 13.501$$

$$s = \sqrt{13.501(13.501-9.558)(13.501-5.009)(13.501-12.435)} = 21.952\ A^{\circ 2}$$

Number of atoms in the plane (124) ×area of each atom in the plane (124)

$$=\left[\left(2\times\frac{32}{108}\times 0.5\right)+\left(\frac{68}{108}\times\frac{139}{148}\right)+\left(\frac{52}{108}\times\frac{139}{148}\right)\right]\times\pi\times(1.4)^2 = 8.25\ \ A^{\circ 2}$$

O atom no.1 — O atom no.2 — O atom no.3

Planar density= $\frac{[Number\ \ of\ atoms\ \ in\ plane\ (124)\times area\ \ of\ \ each\ atom\ \ in\ the\ plane\ (124)]}{area\ \ of\ \ the\ plane\ \ (124)} = \frac{8.25}{21.952} = 0.376$

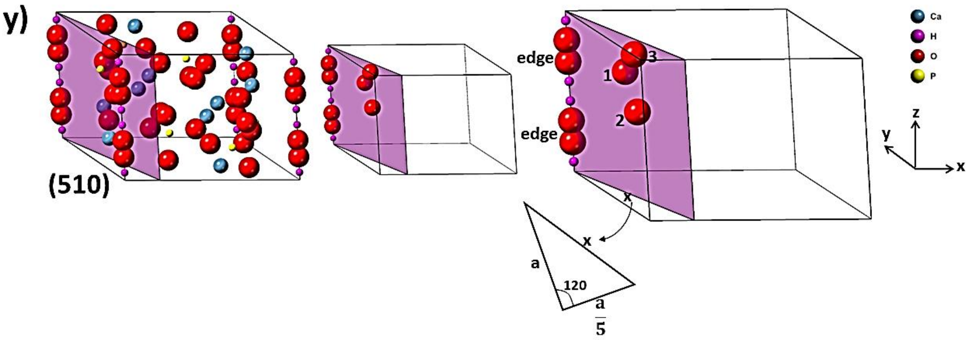

Cosine's rule: $x^2 = \left(\frac{a}{5}\right)^2 + a^2 - 2\left(\frac{a}{5}\right)(a)cos120^{\circ} = \left(\frac{9.4}{5}\right)^2 + (9.4)^2 - 2\times\left(\frac{9.4}{5}\right)\times(9.4)\times(-0.5) = 109.566 \rightarrow x = 10.467$

Area of the plane (510):$s = c\times x = (6.93)\times 10.467 = 72.536\ A^{\circ 2}$

Number of atoms in the plane (510) ×area of each atom in the plane (510)

$$=\left[(4\times 0.5)+\left(\frac{76}{128}\right)+\left(\frac{52}{128}\right)+\left(\frac{52}{128}\times\frac{139}{148}\right)\right]\times\pi\times(1.4)^2 + \underbrace{(4\times 0.5\times\pi\times(0.1)^2)}_{\text{H atom}} =$$

O atom edge — O atom no.1 — O atom no.2 — O atom no.3

$20.822 + 0.0628 = 20.885\ A^{\circ 2}$

Planar density= $\frac{[Number\ \ of\ atoms\ \ in\ plane\ (510)\times area\ \ of\ \ each\ atom\ \ in\ the\ plane\ (510)]}{area\ \ of\ \ the\ plane\ \ (510)} = \frac{20.885}{72.536} = 0.288$

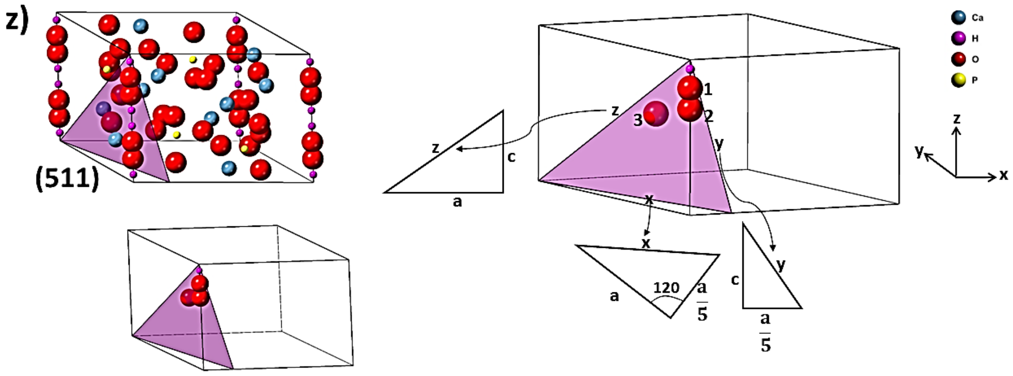

Cosine's rule: $x^2 = \left(\frac{a}{5}\right)^2 + a^2 - 2(a)\left(\frac{a}{5}\right)cos120^{\circ} = \left(\frac{9.4}{5}\right)^2 + (9.4)^2 - 2(9.4) \times \left(\frac{9.4}{5}\right) \times (-0.5) = 109.566 \rightarrow x = 10.467$

$$y^2 = \left(\frac{a}{5}\right)^2 + c^2 = \left(\frac{9.4}{5}\right)^2 + (6.93)^2 = 51.559 \rightarrow y = 7.18$$

$$Z^2 = a^2 + c^2 = (9.4)^2 + (6.93)^2 = 136.385 \rightarrow z = 11.678$$

Area of the plane (511):$s \xrightarrow{Hero\,n's\,law} s = \sqrt{p(p-x)(p-y)(p-z)}$

$$p = \frac{x+y+y}{2} = \frac{10.467 + 7.18 + 11.678}{2} = 14.663$$

$$s = \sqrt{14.663 \times (14.663 - 10.467) \times (14.663 - 7.18) \times (14.663 - 11.678)} = 37.07\ A^{\circ 2}$$

Number of atoms in the plane (511)×area of each atom in the plane (511)

$$=\left[\underbrace{\left(\frac{60}{114} \times 0.5\right)}_{\text{O atom no.1}} + \underbrace{\left(\frac{24}{114} \times 0.5\right)}_{\text{O atom no.2}} + \underbrace{\left(\frac{20}{114}\right)}_{\text{O atom no.3}}\right] \times \pi \times (1.4)^2 + \underbrace{\left(\frac{24}{42} \times 0.5 \times \pi \times (0.1)^2\right)}_{\text{H atom}} =$$

$$3.349 + 0.009 = 3.358\ A^{\circ 2}$$

Planar density$=\frac{[Number\ of\ atoms\ in\ plane\ (511) \times area\ of\ each\ atom\ in\ the\ plane\ (511)]}{area\ of\ the\ plane\ (511)} = \frac{3.358}{37.07} = 0.091$

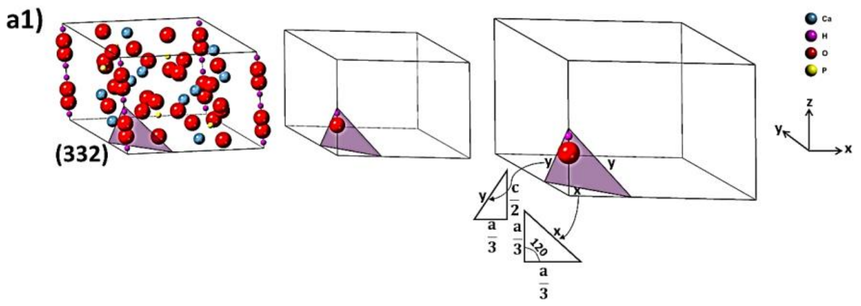

Cosine's rule: $x^2 = \left(\frac{a}{3}\right)^2 + \left(\frac{a}{3}\right)^2 - 2\left(\frac{a}{3}\right)\left(\frac{a}{3}\right) cos120^\circ = \left(\frac{9.4}{3}\right)^2 + \left(\frac{9.4}{3}\right)^2 - 2\left(\frac{9.4}{3}\right)\left(\frac{9.4}{3}\right) \times (-0.5) = 29.453 \rightarrow x = 5.247$

$$y^2 = \left(\frac{a}{3}\right)^2 + \left(\frac{c}{2}\right)^2 = \left(\frac{9.4}{3}\right)^2 + \left(\frac{6.93}{2}\right)^2 = 21.824 \rightarrow y = 4.672$$

Area of the plane (332): $s \xrightarrow{Hero\,n's\,law} s = \sqrt{p(p-x)(p-y)(p-y)}$

$$p = \frac{x+y+y}{2} = \frac{5.427 + 4.672 + .4.672}{2} = 7.385 \rightarrow$$

$$s = \sqrt{7.385(7.385 - 5.427) \times (7.385 - 4.672) \times (7.385 - 4.672)} = 10.317\ A^{\circ 2}$$

Number of atoms in the plane (332)×area of each atom in the plane (332)

$$= \underbrace{\left[\left(\frac{8}{100} \times 0.5\right) \times \pi \times (1.4)^2\right]}_{\text{O atom}} + \underbrace{\left[\frac{4}{19} \times 0.5 \times \pi \times (0.1)^2\right]}_{\text{H atom}} = 0.246 + 0.003 = 0.249\ A^{\circ 2}$$

$$\text{Planar density} = \frac{[Number\ \ of\ atoms\ \ in\ plane\ (332) \times area\ \ of\ each\ atom\ \ in\ the\ plane\ (332)]}{area\ \ of\ the\ plane\ \ (332)} = \frac{0.249}{10.317} = 0.024$$

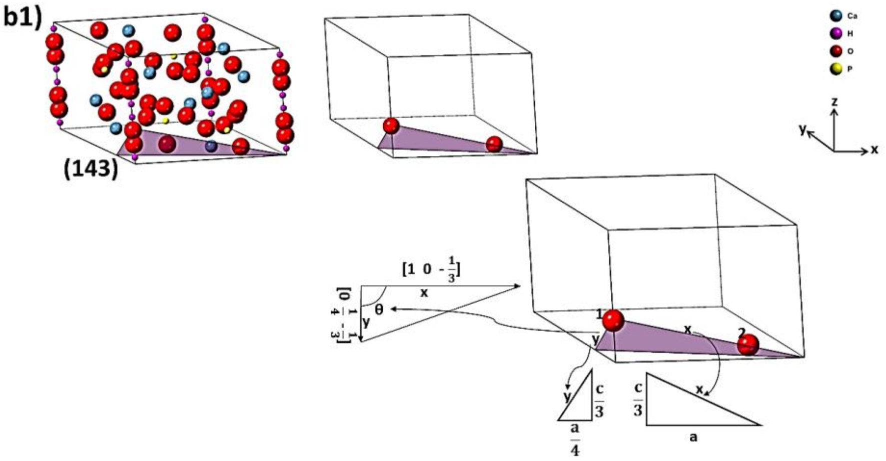

$$x^2 = \left(\frac{c}{3}\right)^2 + a^2 = \left(\frac{6.93}{3}\right)^2 + (9.4)^2 = 93.696 \rightarrow x = 9.680$$

$$y^2 = \left(\frac{c}{3}\right)^2 + \left(\frac{a}{4}\right)^2 = \left(\frac{6.93}{3}\right)^2 + \left(\frac{9.4}{4}\right)^2 = 10.859 \rightarrow y = 3.295$$

Angle between two miller hexagonal directions:

$$[u_1\ v_1\ w_1] < [u_2\ v_2\ w_2] \quad \left[1\ \ 0\ \ \frac{-1}{3}\right] \overset{?}{<} \left[0\ \ \frac{1}{4}\ \ \frac{-1}{3}\right]$$

$$cos\theta = \frac{a^2\left[u_1u_2 + v_1v_2 - \frac{1}{2}(u_1v_2 + v_1u_2)\right] + c^2w_1w_2}{\sqrt{a^2(u_1^2 - u_1v_1 + v_1^2) + c^2w_1^2}\ \sqrt{a^2(u_2^2 - u_2v_2 + v_2^2) + c^2w_2^2}}$$

$$= \frac{(9.4)^2\left[0 + 0 - \frac{1}{2}\left((1)(\frac{1}{4}) + 0\right)\right] + (6.93)^2(\frac{-1}{3})(\frac{-1}{3})}{\sqrt{(9.4)^2(1 - 0 + 0) + (6.93)^2\left(\frac{-1}{3}\right)^2}\ \sqrt{(9.4)^2\left(0 - 0 + \left(\frac{-1}{4}\right)^2\right) + (6.93) \times \left(\frac{-1}{3}\right)^2}}$$

$$= \frac{(9.4)^2 \times \left(\frac{-1}{8}\right) + \frac{(6.93)^2}{9}}{\sqrt{(9.4)^2 + (6.93)^2 \times (\frac{1}{9})}\sqrt{(9.4)^2\left(\frac{1}{16}\right) + (6.93)^2(\frac{1}{9})}} = \frac{-5.71}{(9.68) \times (3.295)} = -0.179$$

$$\rightarrow \theta = 100.313°$$

Area of the plane (143): $S = 0.5 \times (x) \times (y) \times \sin\theta = 0.5 \times (9.68) \times (3.295) \times \sin(100.313) = 15.96\ \text{A}^{\circ 2}$

Number of atoms in the plane (143) ×area of each atom in the plane (143)

$$= \left[\left(\underset{\text{O atom no.1}}{\frac{88}{102} \times \frac{100.313}{360}}\right) + \left(\underset{\text{O atom no.2}}{\frac{28}{102} \times \frac{139}{148}}\right)\right] \times \pi \times (1.4)^2 = 3.068\ A^{\circ 2}$$

Planar density$= \frac{[Number\ \ of\ \ atoms\ \ in\ plane\ (143) \times area\ \ of\ \ each\ atom\ \ in\ the\ plane\ (143)]}{area\ \ of\ \ the\ plane\ \ (143)} = \frac{3.068}{15.69} = 0.196$

Figure 6. Array and position of the involved atoms such as (**a**) 210, (**b**) 211, (**c**) 112, (**d**) 300, (**e**) 202, (**f**) (301), (**g**) (130), (**h**) (310), (**i**) (032), (**j**) (040), (**k**) (023), (**l**) (222), (**m**) (320), (**n**) (230), (**o**) (213), (**p**) (321), (**q**) (042), (**r**) (033), (**s**) (004), (**t**) (050), (**u**) (501), (**v**) (331), (**w**) (043), (**x**) (124), (**y**) (510), (**z**) (511), (**a1**) (332) and (**b1**) (143) for unit-cell of hydroxyapatite.

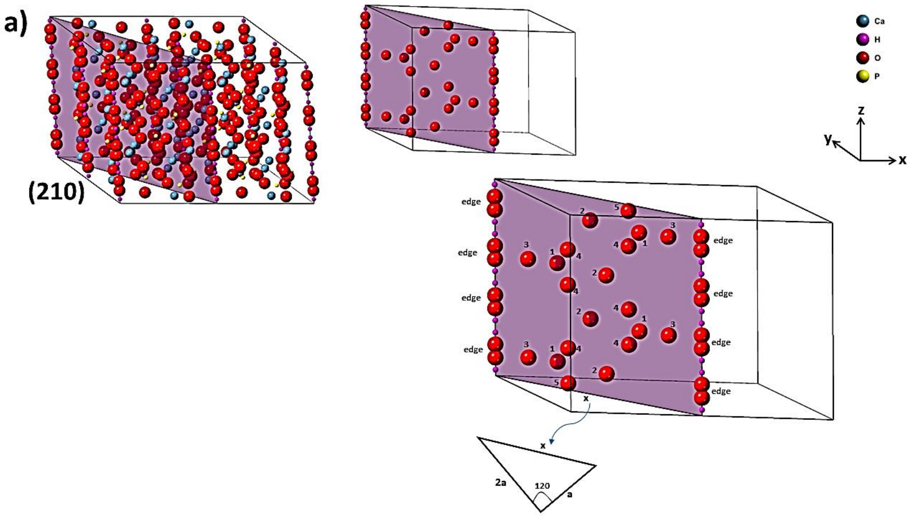

Cosine's law: $x^2 = (2a)^2 + (a)^2 - 2(2a)(a)cos120^{\circ} = (18.8)^2 + (9.4)^2 - 2(18.8)(9.4) \times (-0.5) = 618.52 \rightarrow x = 24.87\ A^{\circ}$

Area of the plane (210):$s = x \times (2c) = 24.87 \times (2 \times 6.93) = 344.7\ A^{\circ 2}$

Number of atoms in the plane (210)×area of each atom in the plane (210)

$$=\underbrace{\left[\left(16 \times \frac{1}{2}\right) \times \pi \times (0.1)^2\right]}_{\text{H atoms}} + \left[\underbrace{\left(16 \times \frac{1}{2}\right)}_{\text{O edge atom}} + \underbrace{\left(\frac{15}{61} \times 4\right)}_{\text{O atom no.1}} + \underbrace{\left(4 \times \frac{20}{61}\right)}_{\text{O atom no.2}} + \underbrace{\left(4 \times \frac{58}{61}\right)}_{\text{O atom no.3}} +\right.$$

$$\left.\underbrace{\left(6 \times \frac{42}{61}\right)}_{\text{O atom no.4}} + \underbrace{\left(2 \times \frac{42}{61} \times \frac{139}{148}\right)}_{\text{O atom no.5}}\right] \times \pi \times (1.4)^2 = 0.251 + 120.212 = 120.463\ A^{\circ 2}$$

$$\text{Planar density} = \frac{[Number\ \ of\ \ atoms\ \ in\ \ plane\ (210) \times area\ \ of\ \ each\ atom\ \ in\ the\ plane\ (210)]}{area\ \ of\ \ the\ plane\ \ (210)} = \frac{120.463}{344.7} = 0.349$$

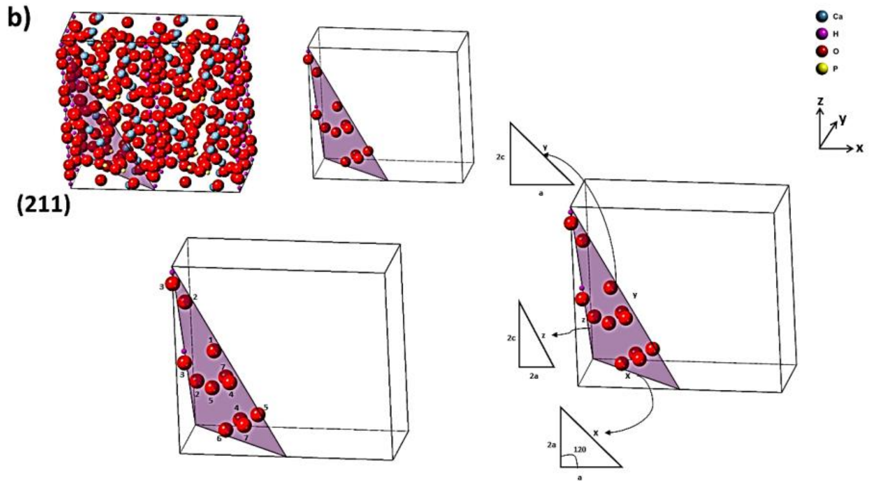

Cosine's law: $x^2 = (a)^2 + (2a)^2 - 2(a)(2a)cos120^{\circ} = (9.4)^2 + (18.8)^2 - 2 \times (9.4) \times (18.8) \times (-0.5) = 618.52 \rightarrow x = 24.87$

$y^2 = (2c)^2 + (a)^2 = (2 \times 6.93)^2 + (9.4)^2 = 280.46 \rightarrow y = 16.75$

$Z^2 = (2a)^2 + (2c)^2 = (18.8)^2 + (2 \times 6.93)^2 = 545.54 \rightarrow z = 23.36$

Area of the plane (211):$s \xrightarrow{Hero\,n'\,s\,law} s = \sqrt{p(p-x)(p-y)(p-z)}$

$$p = \frac{x+y+y}{2} = \frac{(24.87) + (16.75) + (23.36)}{2} = 32.49$$

$$s = \sqrt{32.49 \times (32.49 - 24.87) \times (32.49 - 16.75) \times (32.49 - 23.36)}$$

$s = 188.62\ A^{\circ 2}$

Number of atoms in the plane (211)×area of each atom in the plane (211)

$$=\left[\underbrace{\left(\frac{48}{50}\right)}_{\text{O atom no.1}} + \underbrace{\left(2 \times \frac{30}{50}\right)}_{\text{O atom no.2}} + \underbrace{\left(2 \times \frac{4}{50}\right)}_{\text{O atom no.3}} + \underbrace{\left(2 \times \frac{20}{50}\right)}_{\text{O atom no.4}} + \underbrace{(2 \times 1)}_{\text{O atom no.5}} + \underbrace{\left(\frac{48}{50} \times \frac{139}{148}\right)}_{\text{O atom no.6}} + \underbrace{\left(2 \times \frac{18}{50}\right)}_{\text{O atom no.7}}\right] \times$$

$$\pi \times (1.4)^2 + \underbrace{\left(2 \times \frac{1}{9}\right) \times \pi \times (0.1)^2}_{\text{H atom}} = 41.512 + 0.007 = 41.519\ A^{\circ 2}$$

$$\text{Planar density} = \frac{[Number\ of\ atoms\ in\ plane\ (211) \times area\ of\ each\ atom\ in\ the\ plane\ (211)]}{area\ of\ the\ plane\ (211)} = \frac{41.519}{188.62} = 0.22$$

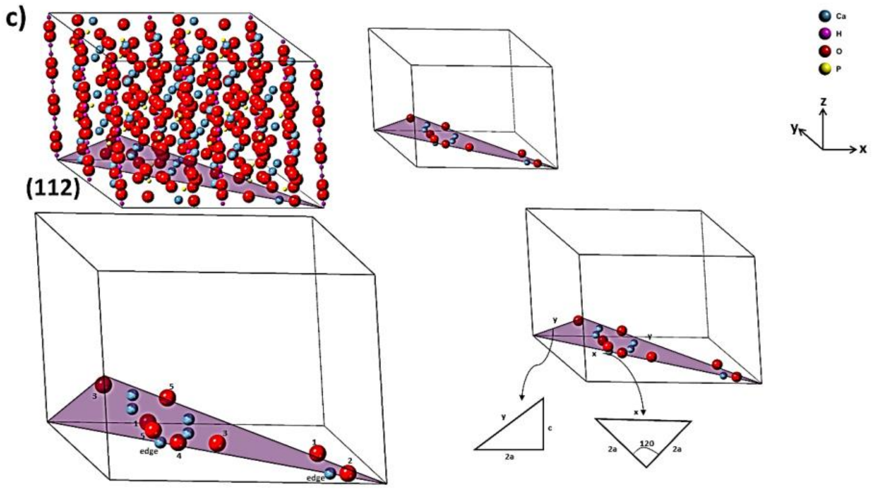

Cosine rule: $x^2 = (2a)^2 + (2a)^2 - 2 \times (2a) \times (2a) \times cos120^{\circ} = 12a^2$

$$\rightarrow x = 2\sqrt{3}a = 2 \times \sqrt{3} \times (9.4) = 32.563$$

$y^2 = (2a)^2 + c^2 = (18.8)^2 + (6.93)^2 = 401.465 \rightarrow y = 20.037$

Area of the plane (112): $s \xrightarrow{Hero\,n's\,law} s = \sqrt{p(p-x)(p-y)(p-y)}$

$$p = \frac{x+y+y}{2} = \frac{32.563 + 20.037 + 20.037}{2} \rightarrow p = 36.319$$

$$s = \sqrt{36.319(36.319 - 32.563)(36.319 - 20.037)^2} \rightarrow s = 190.168\ A^{\circ 2}$$

Number of atoms in the plane (112)×area of each atom in the plane (112)

$$= \Big[\underbrace{\left(0.5 \times \frac{60}{67} \times 2\right)}_{\text{Ca atom edge}} + \underbrace{(4 \times 1)}_{\text{Other Ca atoms}}\Big] \times \pi \times (1)^2 + \Big[\underbrace{\left(2 \times \frac{12}{51}\right)}_{\text{O atom no.1}} + \underbrace{\left(\frac{43}{51} \times \frac{139}{148}\right)}_{\text{O atom no.2}} + \underbrace{\left(2 \times \frac{2}{51}\right)}_{\text{O atom no.3}} +$$

$$\underbrace{\left(\frac{46}{51}\right)}_{\text{O atom no.4}} + \underbrace{\left(2 \times \frac{16}{51}\right)}_{\text{O atom no.5}}\Big] \times \pi \times (1.4)^2 = 15.380 + 17.678 = 33.054\ A^{\circ 2}$$

Planar density =

$$= \frac{[Number\ of\ atoms\ in\ plane(112) \times area\ of\ each\ atom\ in\ the\ plane(112)]}{area\ of\ the\ plane\ (112)}$$

$= 0.174$

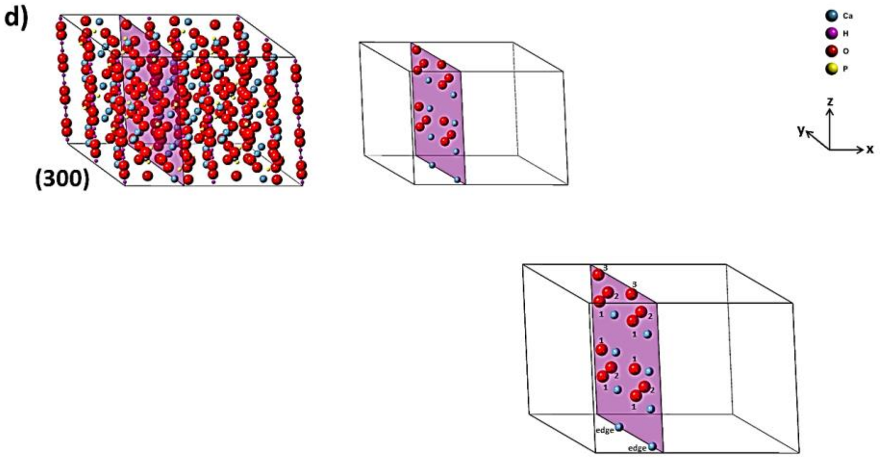

Area of the (300): $s = 2a \times 2c = (18.8) \times (6.93 \times 2) = 260.568\ \text{A}^{\circ 2}$

Number of atoms in the plane (300) × area of each atom in the plane (300)

$$= \left[\underbrace{\left(2 \times \frac{1}{2}\right)}_{\text{Ca atom edge}} + \underbrace{(6 \times 1)}_{\text{Other Ca atom}}\right] \times \pi \times (1)^2 + \left[\underbrace{\left(6 \times \frac{122}{139}\right)}_{\text{O atom no.1}} + \underbrace{\left(4 \times \frac{126}{139}\right)}_{\text{O atoms no.2}} + \underbrace{\left(2 \times \frac{122}{139} \times \frac{139}{148}\right)}_{\text{O atoms no.3}}\right] \times$$

$$\pi \times (1.4)^2 = 21.99 + 64.905 = 86.9\ A^{\circ 2}$$

Planar density

$$= \frac{[Number\ of\ atoms\ in\ plane(300) \times area\ of\ each\ atom\ in\ the\ plane(300)]}{area\ of\ the\ plane\ (300)}$$

$$= \frac{86.9}{260.568} = 0.33$$

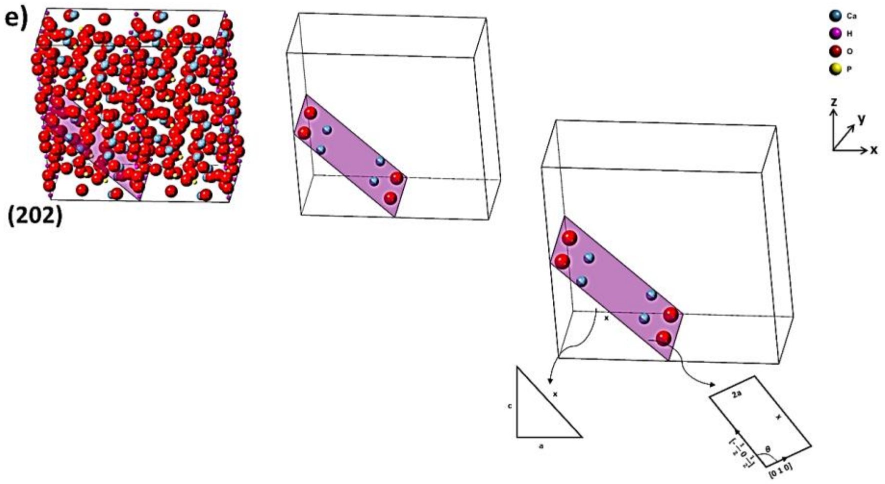

$x^2 = a^2 + c^2 = (9.4)^2 + (6.93)^2 = 136.385 \rightarrow x = 11.678$

Angle between two miller hexagonal directions:

$[u_1\ v_1\ w_1] < [u_2\ v_2\ w_2] \quad [0\ 1\ 0] \measuredangle \left[-\frac{1}{2}\ 0\ \frac{1}{2}\right]$

$$cos\theta = \frac{a^2\left[u_1u_2 + v_1v_2 - \frac{1}{2}(u_1v_2 + v_1u_2)\right] + c^2w_1w_2}{\sqrt{a^2(u_1^2 - u_1v_1 + v_1^2) + c^2w_1^2}\ \sqrt{a^2(u_2^2 - u_2v_2 + v_2^2) + c^2w_2^2}}$$

$$cos\theta = \frac{(9.4)^2\left[0 + 0 - \frac{1}{2}\left(0 - \frac{1}{2}\right)\right] + 0}{\sqrt{(9.4)^2(0 - 0 + 1^2) + (6.93)^2 * 0}\ \sqrt{(9.4)^2\left(\left(\frac{-1}{2}\right)^2 + 0 + 0\right) + (6.93)^2\left(\frac{1}{2}\right)^2}}$$

$$cos\theta = \frac{(9.4)^2\left(\frac{1}{4}\right)}{\sqrt{(9.4)^2}\sqrt{(9.4)^2\left(\frac{1}{2}\right)^2 + (6.93)^2\left(\frac{1}{2}\right)^2}} = 0.4 \rightarrow \theta = 66.3°$$

Area of the plane (202): $S = (2a) \times (x) \times \sin\theta = (18.8) \times (11.678) \times \sin(66.3°) = 201.03\ A^{\circ 2}$

Number of atoms in the plane (202)×area of each atom in the plane (202)

$$= \underbrace{4 \times 1 \times \pi \times (1)^2}_{\text{Ca atom}} + \underbrace{\left[\left(4 \times \frac{48}{54}\right)\right] \times \pi \times (1.4)^2}_{\text{O atoms}} = 12.566 + 21.893 = 34.459\ A^{\circ 2}$$

Planar density$= \frac{[Number\ of\ atoms\ in\ plane\ (202) \times area\ of\ each\ atom\ in\ the\ plane\ (202)]}{area\ of\ the\ plane\ (202)} = \frac{34.459}{201.03} = 0.171$

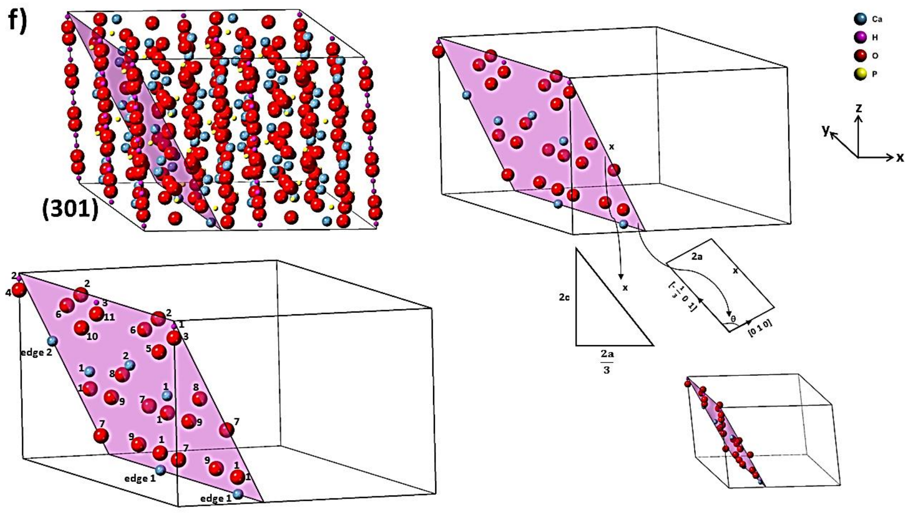

$$x^2 = \left(\frac{2a}{3}\right)^2 + (2c)^2 = \left(\frac{18.8}{3}\right)^2 + (2 * 6.93)^2 = 231.371$$

$\rightarrow x = 15.211$

Angle between two miller hexagonal directions:

$[u_1\ v_1\ w_1] < [u_2\ v_2\ w_2] \quad \left[\frac{-1}{3}\ 0\ 1\right] \measuredangle [0\ 1\ 0]$

$$cos\theta = \frac{a^2\left[u_1u_2 + v_1v_2 - \frac{1}{2}(u_1v_2 + v_1u_2)\right] + c^2w_1w_2}{\sqrt{a^2(u_1^2 - u_1v_1 + v_1^2) + c^2w_1^2}\ \sqrt{a^2(u_2^2 - u_2v_2 + v_2^2) + c^2w_2^2}}$$

$$= \frac{(9.4)^2\left[0 + 0 - \frac{1}{2}\left(\frac{-1}{3} + 0\right)\right] + (6.93)^2(0)}{\sqrt{(9.4)^2\left(\left(\frac{-1}{3}\right)^2 - \left(\frac{-1}{3}\times 0\right) + 0\right) + (6.93)^2(1)^2}\ \sqrt{(9.4)^2(0 - 0 + 1) + (6.93)^2 \times 0}}$$

$$= \frac{(9.4)^2\left(\frac{1}{6}\right)}{\sqrt{(9.4)^2\left(\frac{1}{9}\right) + (6.95)^2}\ \sqrt{(9.4)^2}} = 0.206 \rightarrow \theta = 78.112°$$

Area of the plane (301): $S = (2a) \times (x) \times \sin 78.112° = 279.833 A^{\circ 2}$

$$\Big[\underset{\text{O atom no.1}}{\left(\frac{44}{112}\times 4\right)} + \underset{\text{O atom no.2}}{\left(\frac{22}{112}\times\frac{139}{148}\times 2\right)} + \underset{\text{O atom no.3}}{\left(\frac{20}{112}\times\frac{139}{148}\right)} + \underset{\text{O atom no.4}}{\left(\frac{6}{112}\times\frac{139}{148}\right)} + \underset{\text{O atom no.5}}{\left(\frac{24}{112}\right)}$$

$$+ \underset{\text{O atom no.6}}{\left(\frac{60}{112}\times 2\right)} + \underset{\text{O atom no.7}}{\left(\frac{12}{112}\times 4\right)} + \underset{\text{O atom no.8}}{\left(\frac{64}{112}\times 2\right)} + \underset{\text{O atom no.9}}{\left(\frac{108}{112}\times 4\right)} + \underset{\text{O atom no.10}}{\left(\frac{28}{112}\right)}$$

$$+ \underset{\text{O atom no.11}}{\left(\frac{20}{112}\right)}\Big] \times \pi \times (1.4)^2$$

$$+ \Big[\underset{\text{Ca atom edge 1}}{\left(2 \times 0.5 \times \frac{76}{78}\right)} + \underset{\text{Ca atom edge 2}}{\left(0.56 \times \frac{64}{78}\right)} + \underset{\text{Ca atom no.1}}{(2 \times 1)} + \underset{\text{Ca atom no.2}}{\left(\frac{72}{78}\right)}\Big] \times \pi \times (1)^2$$

$$+ \Big[\underset{\text{H atom no.1}}{\left(\frac{10}{27} * 0.5\right)} + \underset{\text{H atom no.2}}{\left(\frac{4}{27} * 0.5\right)} + \underset{\text{H atom no.3}}{\left(\frac{10}{27}\right)}\Big] * \pi * (0.1)^2$$

$$= 57.273 + 13.688 + 0.0198 = 70.981$$

Planar density= $\frac{[Number\ of\ atoms\ in\ plane(301) \times area\ of\ each\ atom\ in\ the\ plane(301)]}{area\ of\ the\ plane\ (301)} = \frac{70.981}{279.833} = 0.254$

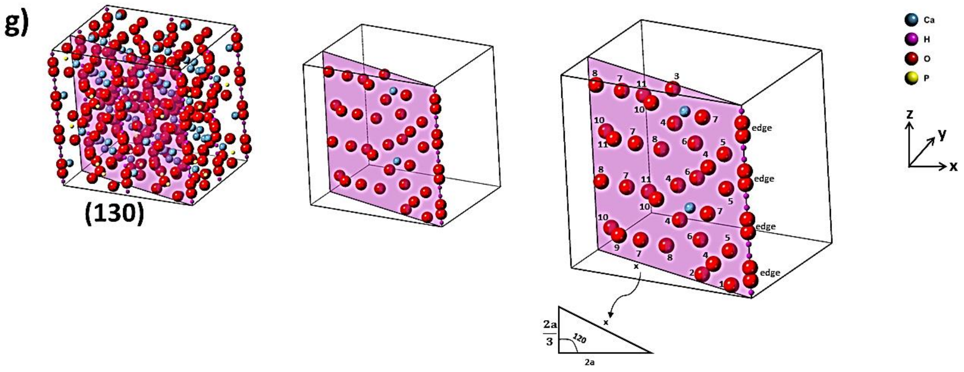

Cosine's rule: $x^2 = (2a)^2 + \left(\frac{2a}{3}\right)^2 - 2(2a)\left(\frac{2a}{3}\right)cos120^{\circ} = (18.8)^2 + \left(\frac{18.8}{3}\right)^2 - 2(18.8)\left(\frac{18.8}{3}\right)cos120^{\circ} = 510.524 \rightarrow x = 22.595$

Area of the plane (130):$s = x \times (2c) = 22.595 \times (2 \times 6.93) = 313.164A^{\circ 2}$

Number of atoms in the plane (130) ×area of each atom in the plane (130)

$$=\Big[\underbrace{\left(8\times\frac{1}{2}\right)}_{\text{O atom edge}}+\underbrace{\left(\frac{122}{125}\times\frac{139}{148}\right)}_{\text{O atom no.1}}+\underbrace{\left(\frac{68}{125}\times\frac{139}{148}\right)}_{\text{O atom no.2}}+\underbrace{\left(\frac{70}{125}\times\frac{139}{148}\right)}_{\text{O atom no.3}}+\underbrace{\left(\frac{70}{125}\times 5\right)}_{\text{O atom no.4}}+\underbrace{\left(\frac{122}{125}\times 3\right)}_{\text{O atom no.5}}+$$

$$\underbrace{\left(\frac{68}{125}\times 3\right)}_{\text{O atom no.6}}+\underbrace{\left(\frac{104}{125}\times 6\right)}_{\text{O atom no.7}}+\underbrace{\left(\frac{52}{125}\times 4\right)}_{\text{O atom no.8}}+\underbrace{\left(\frac{32}{125}\right)}_{\text{O atom no.9}}+\underbrace{\left(\frac{36}{125}\times 4\right)}_{\text{O atom no.10}}+\underbrace{\left(\frac{16}{125}\times 3\right)}_{\text{O atom no.11}}\Big]\times\pi\times$$

$$(1.4)^2+\underbrace{\left[2\times\left(\frac{8}{9}\right)\times\pi\times(1)^2\right]}_{\text{Ca atom}}+\underbrace{\left[8\times\frac{1}{2}\times\pi\times(0.1)^2\right]}_{\text{H atoms}}=133.997+5.585+$$

$0.126 = 139.708A^{\circ 2}$

Planar density=$\frac{[Number\ \ of\ \ atoms\ \ in\ plane\ (130)\times area\ \ of\ \ each\ atom\ \ in\ the\ plane\ (130)]}{area\ \ of\ \ the\ plane\ \ (130)} = \frac{139.708}{313.164} = 0.446$

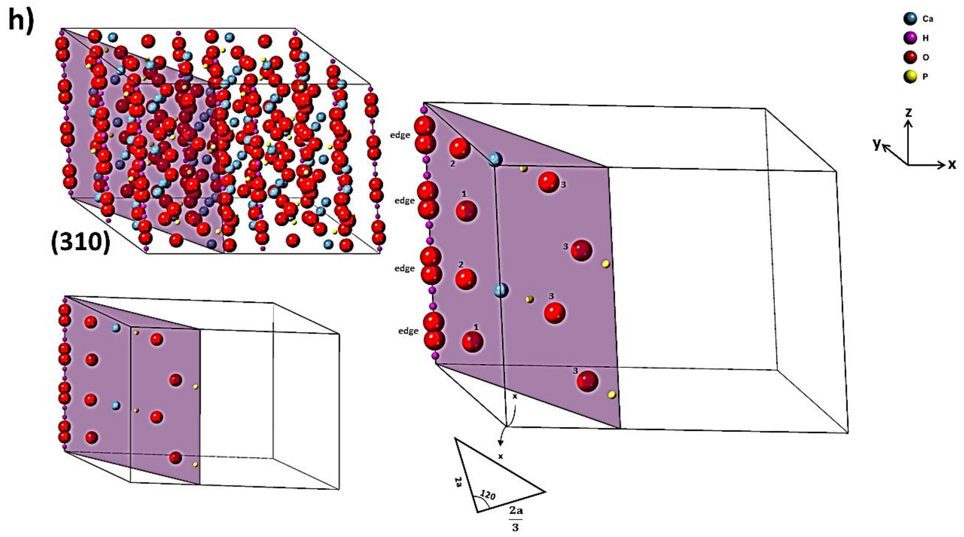

Cosine's rule: $x^2 = (2a)^2 + \left(\frac{2a}{3}\right)^2 - 2(2a)\left(\frac{2a}{3}\right)cos120^{\circ} = (18.8)^2 + \left(\frac{18.8}{3}\right)^2 - 2(18.8)\left(\frac{18.8}{3}\right) * (-0.5) = 510.524 \to x = 22.595$

Area of the plane (310):$s = x * (2c) = 22.595 * (2 * 6.93) = 313.167\ A^{\circ 2}$

Number of atoms in the plane (310) ×area of each atom in the plane (310)

$$=\Big[\underbrace{\left(8*\tfrac{1}{2}\right)}_{\text{O atom edge}} + \underbrace{\left(2*\tfrac{52}{125}\right)}_{\text{O atom no.1}} + \underbrace{\left(2*\tfrac{90}{125}\right)}_{\text{O atom no.2}} + \underbrace{\left(4*\tfrac{28}{125}\right)}_{\text{O atom no.3}}\Big] * \pi * (1.4)^2 + \underbrace{(2*1)*\pi*(1)^2}_{\text{Ca atom}} + \underbrace{\left(4*\tfrac{6}{23}\right)*\pi*(0.31)^2}_{\text{P atom}} + \underbrace{\left(8*\tfrac{1}{2}\right)*\pi*(0.1)^2}_{\text{H atoms}} = 44.137 + 6.283 + 0.315 + 0.126 = 50.861\ A^{\circ 2}$$

Planar density=$\frac{[Number\ \ of\ atoms\ \ in\ plane\ (310)*area\ \ of\ each\ atom\ in\ the\ plane\ (310)]}{area\ \ of\ the\ plane\ \ (310)} = \frac{50.861}{313.167} = 0.162$

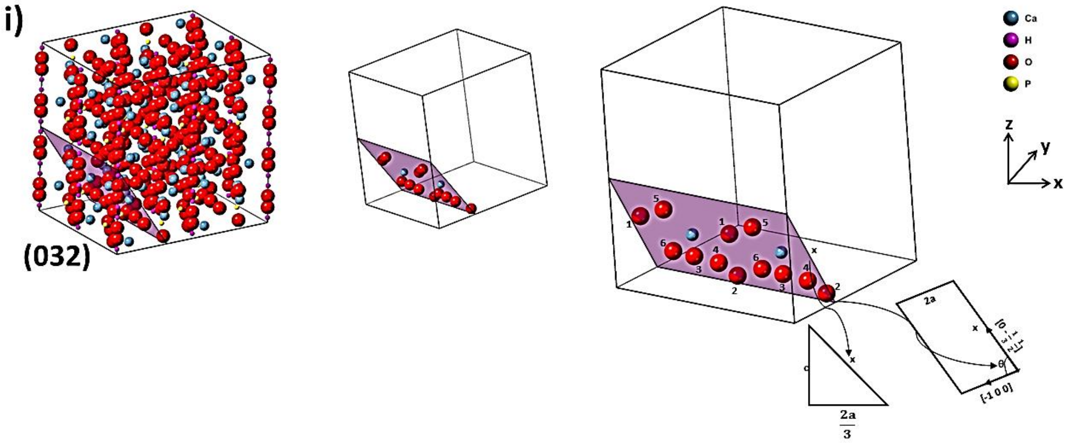

$$x^2 = \left(\frac{2a}{3}\right)^2 + (c)^2 = \left(\frac{18.8}{3}\right)^2 + (6.93)^2 = 87.296 \rightarrow x = 9.343$$

Angle between two miller hexagonal directions:

$$[u_1\, v_1\, w_1] < [u_2\, v_2\, w_2] \quad [-1\;0\;0] \lessdot \left[0\; \frac{-1}{3}\; \frac{1}{2}\right]$$

$$cos\theta = \frac{a^2\left[u_1u_2 + v_1v_2 - \frac{1}{2}(u_1v_2 + v_1u_2)\right] + c^2w_1w_2}{\sqrt{a^2(u_1^2 - u_1v_1 + v_1^2) + c^2w_1^2}\,\sqrt{a^2(u_2^2 - u_2v_2 + v_2^2) + c^2w_2^2}}$$

$$= \frac{(9.4)^2\left[0 + 0 - \frac{1}{2}\left((-1)(\frac{-1}{3}) + 0\right)\right] + (6.93)^2 * (0)}{\sqrt{(9.4)^2((-1)^2 - 0 + 0) + 0}\,\sqrt{(9.4)^2\left(0 - 0 + \left(\frac{-1}{3}\right)^2\right) + (6.93)^2 * \left(\frac{1}{2}\right)^2}}$$

$$= \frac{(9.4)^2\left(\frac{-1}{6}\right)}{(6.4)\sqrt{(9.4)^2\left(\frac{1}{9}\right) + (6.93)^2(\frac{1}{4})}} = -0.335 \rightarrow \theta = 109.573°$$

Area of the plane (032): $S = (2a) * (x) * \sin 78.112° = (18.8) * (9.343) * \sin(109.573°) = 165.499 A^{°2}$

Number of atoms in the plane (032)×area of each atom in the plane (032)

$$=\left[\underbrace{\left(\frac{76}{106} * 2\right)}_{\text{O atom no.1}} + \underbrace{\left(\frac{60}{106} * \frac{139}{148} * 2\right)}_{\text{O atom no.2}} + \underbrace{\left(\frac{62}{106} * 2\right)}_{\text{O atom no.3}} + \underbrace{\left(\frac{12}{106} * 2\right)}_{\text{O atom no.4}} + \underbrace{\left(\frac{96}{106} * 2\right)}_{\text{O atom no.5}} + \underbrace{\left(\frac{88}{106} * 2\right)}_{\text{O atom no.6}}\right] *$$

$$\pi * (1.4)^2 + \underbrace{[2 * 1 * \pi * (1)^2]}_{\text{Ca atom}} = 45.351 + 6.283 = 51.634\, A^{°2}$$

$$\text{Planar density} = \frac{[Number\ \ of\ \ atoms\ \ in\ plane\ (032) * area\ \ of\ each\ atom\ \ in\ the\ plane\ (032)]}{area\ \ of\ \ the\ plane\ \ (032)} = \frac{51.634}{165.499} = 0.312$$

j)

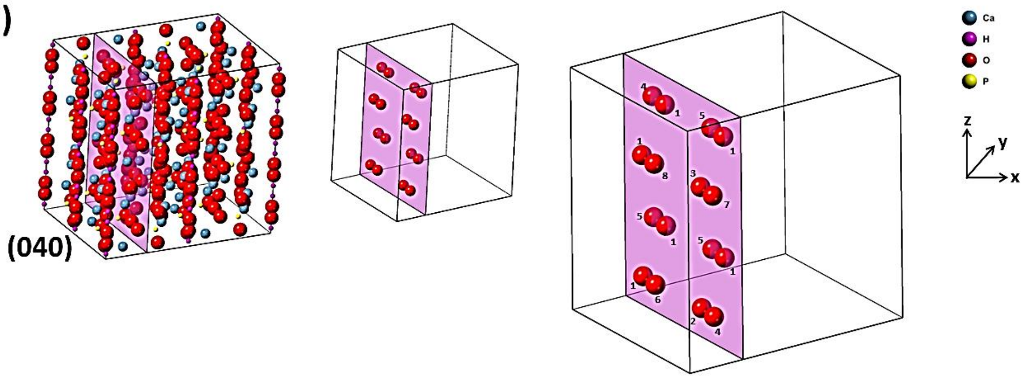

Area of the (040): s= (2a)×(2c) = (18.8)×(2×6.93) =260.568 $A^{°2}$

Number of atoms in the plane (040)×area of each atom in the plane (040)

$$=\left[\underbrace{\left(\frac{108}{38} * 6\right)}_{\text{O atom no.1}} + \underbrace{\left(\frac{122}{138}\right)}_{\text{O atom no.2}} + \underbrace{\left(\frac{112}{138}\right)}_{\text{O atom no.3}} + \underbrace{\left(\frac{72}{138} * 2\right)}_{\text{O atom no.4}} + \underbrace{\left(\frac{68}{138} * 3\right)}_{\text{O atom no.5}} + \underbrace{\left(\frac{88}{138}\right)}_{\text{O atom no.6}} + \underbrace{\left(\frac{76}{138}\right)}_{\text{O atom no.7}} + \underbrace{(\frac{64}{138})}_{\text{O atom no.8}}\right] *$$

$$\pi * (1.4)^2 = 65.056\, A^{°2}$$

$$\text{Planar density} = \frac{[Number\ \ of\ \ atoms\ \ in\ plane\ (040) * area\ \ of\ each\ atom\ \ in\ the\ plane\ (040)]}{area\ \ of\ \ the\ plane\ \ (040)} = \frac{65.056}{260.568} = 0.250$$

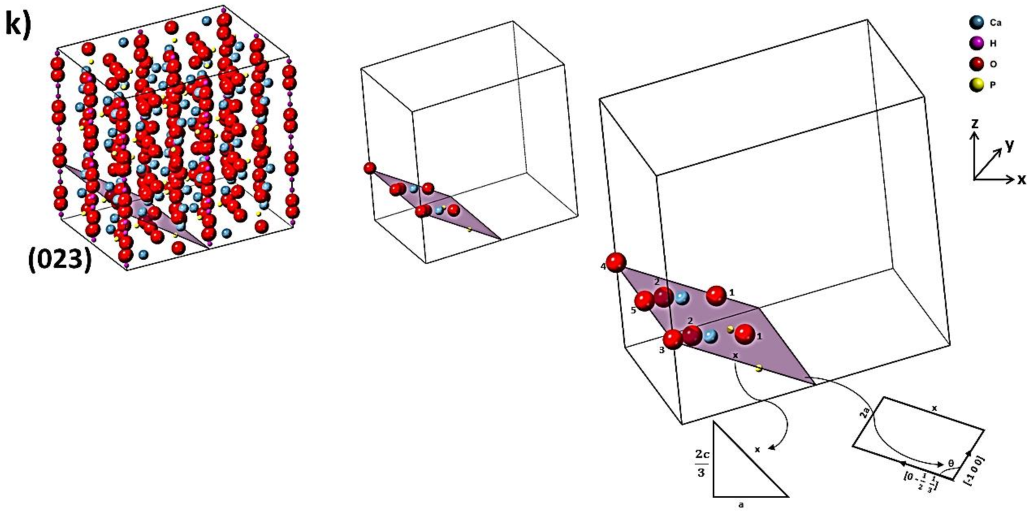

$$x^2 = (a)^2 + \left(\frac{2c}{3}\right)^2 = (9.4)^2 + \left(\frac{2*6.93}{3}\right)^2 = 109.704 \rightarrow x = 10.474$$

Angle between two miller hexagonal directions:

$[u_1\ v_1\ w_1] < [u_2\ v_2\ w_2]$ $[-1\ 0\ 0] \overset{?}{<} \left[0\ \ \frac{-1}{2}\ \ \frac{1}{3}\right]$

$$cos\theta = \frac{a^2\left[u_1u_2 + v_1v_2 - \frac{1}{2}(u_1v_2 + v_1u_2)\right] + c^2w_1w_2}{\sqrt{a^2(u_1^2 - u_1v_1 + v_1^2) + c^2w_1^2}\sqrt{a^2(u_2^2 - u_2v_2 + v_2^2) + c^2w_2^2}}$$

$$= \frac{(9.4)^2\left[0 + 0 - \frac{1}{2}\left((-1)(\frac{-1}{2}) + 0\right)\right] + (6.93)^2(0)}{\sqrt{(9.4)^2((-1)^2 - 0 + 0) + 0}\sqrt{(9.4)^2\left(0 - 0 + (\frac{-1}{2})\right) + (6.93)^2\left(\frac{1}{3}\right)^2}}$$

$$= \frac{(9.4)^2\left(\frac{-1}{4}\right)}{(9.4)\sqrt{(9.4)^2\left(\frac{1}{4}\right) + (6.93)^2 * \frac{1}{9}}} = -0.449 \rightarrow \theta = 116.662°$$

Area of the plane (023): $S = (2a) * (x) * \sin(116.662°) = (18.8) * (10.474) * \sin(116.662°) = 175.973\ Å^2$

Number of atoms in the plane (023) ×area of each atom in the plane (023)

$$= \Big[\underbrace{\left(\frac{76}{112} * 2\right)}_{\text{O atom no.1}} + \underbrace{\left(\frac{36}{112} * 2\right)}_{\text{O atom no.2}} + \underbrace{\left(\frac{63.338}{360} * \frac{92}{112}\right)}_{\text{O atom no.3}} + \underbrace{\left(\frac{116.662}{360} * \frac{92}{112}\right)}_{\text{O atom no.4}} + \underbrace{\left(\frac{92}{112} * 0.5\right)}_{\text{O atom no.5}}\Big] * \pi *$$

$$(1.4)^2 + \underbrace{\left[\left(2 * \frac{8}{41}\right)\right] * \pi * (1)^2}_{\text{Ca atom}} + \underbrace{\left[\left(\frac{12}{20}\right) * 2\right] * \pi * (0.31)^2}_{\text{P atom}} = 17.364 + 1.226 +$$

$$0.362 = 18.952 Å^2$$

Planar density= $\frac{[Number\ \ of\ \ atoms\ \ in\ plane\ (023)*area\ \ of\ \ each\ atom\ \ in\ the\ plane\ (023)]}{area\ \ of\ \ the\ plane\ \ (023)} = \frac{18.952}{175.973} = 0.108$

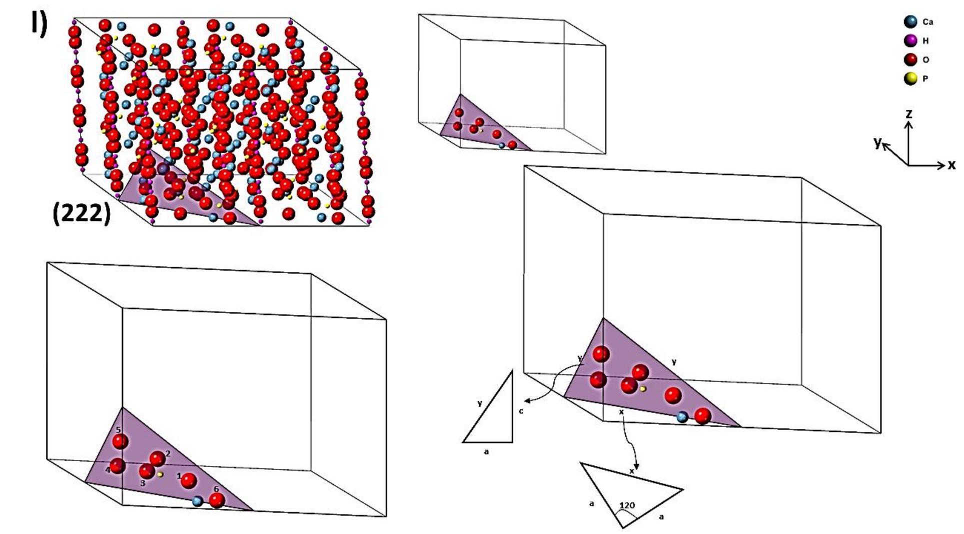

Cosine's rule: $x^2 = (a)^2 + (a)^2 - 2(a)(a)cos120^{\circ} = 3a^2$

$$\rightarrow x = \sqrt{3}a = 16.281$$

$y^2 = (a)^2 + (c)^2 = (9.4)^2 + (6.93)^2 = 136.385 \rightarrow y = 11.678$

Area of the plane (222):$s \xrightarrow{Heron's\ law} s = \sqrt{p(p-x)(p-y)(p-y)}$

$$p = \frac{x+y+y}{2} = 19.819$$

$$s = \sqrt{19.819 * (19.819 - 16.281) * (19.819 - 11.678)^2} = 68.171\ A^{\circ 2}$$

Number of atoms in the plane (222)×area of each atom in the plane (222)

$$= \underbrace{0.57 * \left(\frac{29}{34}\right) * \pi * (1)^2}_{\text{Ca atom}} + \underbrace{\frac{14}{18} * \pi * (0.31)^2}_{\text{P atom}} + \Bigg[\underbrace{\left(\frac{18}{49}\right)}_{\text{O atom no.1}} + \underbrace{\left(\frac{40}{49}\right)}_{\text{O atom no.2}} + \underbrace{\left(\frac{28}{49}\right)}_{\text{O atom no.3}} + \underbrace{\left(\frac{22}{49}\right)}_{\text{O atom no.4}} + \underbrace{(1)}_{\text{O atom no.5}} +$$

$$\underbrace{\left(\frac{14}{49} * \frac{139}{148}\right)}_{\text{O atom no.6}}\Bigg] * \pi * (1.4)^2 = 1.527 + 0.235 + 21.385 = 23.144\ A^{\circ 2}$$

Planar density =

$$= \frac{[Number\ of\ atoms\ in\ plane(222) * area\ of\ each\ atom\ in\ the\ plane(222)]}{area\ of\ the\ plane\ (222)}$$

$$= \frac{23.144}{68.171} = 0.339$$

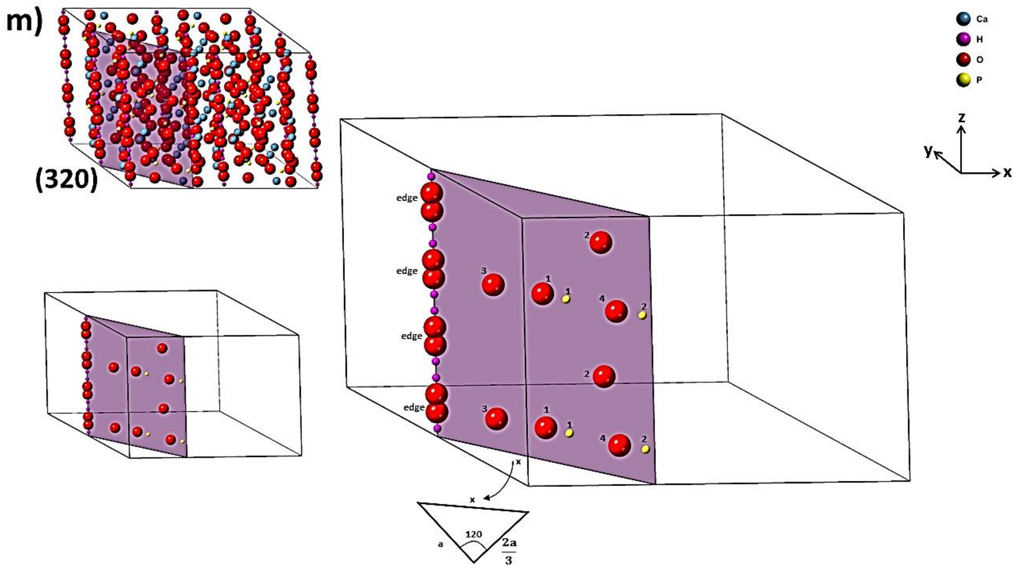

Cosine's rule: $x^2 = (a)^2 + \left(\frac{2a}{3}\right)^2 - 2(a)\left(\frac{2a}{3}\right)cos120^{\circ} = (9.4)^2 + \left(\frac{2*9.4}{3}\right)^2 - 2*(9.4)\left(\frac{2*9.4}{3}\right)cos120^{\circ} = 186.537 \rightarrow x = 13.658$

Area of the plane (320):$s = x*(2c) = 13.658*(2*6.93) = 189.298\ A^{\circ 2}$

Number of atoms in the plane (320)×area of each atom in the plane (320)

$=\left[\underbrace{\left(8*\frac{1}{2}\right)}_{\text{O atom edge}} + \underbrace{\left(2*\frac{52}{60}\right)}_{\text{O atom no.1}} + \underbrace{\left(\frac{2*16}{60}\right)}_{\text{O atom no.2}} + \underbrace{\left(2*\frac{24}{60}\right)}_{\text{O atom no.3}} + \underbrace{\left(\frac{2*32}{60}\right)}_{\text{O atom no.4}}\right]*\pi*(1.4)^2 + \left[\underbrace{\left(2*\frac{6}{23}\right)}_{\text{P atom no.1}} + \underbrace{\left(2*\frac{14}{23}\right)}_{\text{P atom no.2}}\right]*\pi*(0.31)^2 + \underbrace{\left[8*\frac{1}{2}\right]}_{\text{H atoms}}*\pi*(0.1)^2 = 50.081 + 0.525 + 0.126 =$

$50.732\ A^{\circ 2}$

Planar density$=\frac{[Number\ of\ atoms\ in\ plane\ (320)*area\ of\ each\ atom\ in\ the\ plane\ (320)]}{area\ of\ the\ plane\ (320)} = \frac{50.732}{189.298} = 0.268$

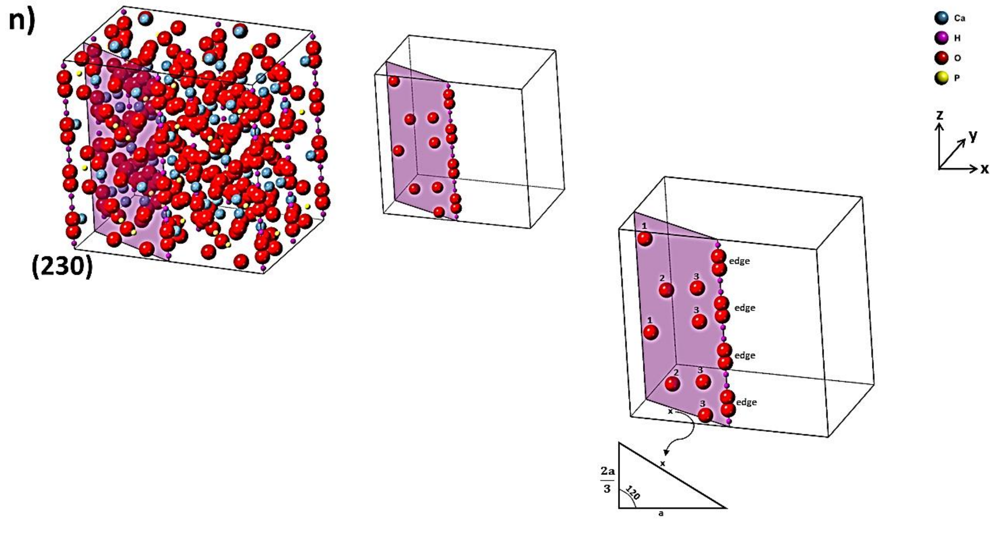

Cosine's rule: $x^2 = (a)^2 + \left(\frac{2a}{3}\right)^2 - 2(a)\left(\frac{2a}{3}\right)cos120^{\circ} = (9.4)^2 + \left(\frac{18.8}{3}\right)^2 - 2(9.4)\left(\frac{18.8}{3}\right)cos120^{\circ} = 186.538 \rightarrow x = 13.659$

Area of the plane (230):$s = x * (2c) = (13.659) * (6.93 * 2) = 189.298 A^{\circ 2}$

Number of atoms in the plane (230) ×area of each atom in the plane (230)

$$=\Big[\underbrace{\left(8 * \frac{1}{2}\right)}_{\text{O atom edge}} + \underbrace{\left(2 * \frac{56}{60}\right)}_{\text{O atom no.1}} + \underbrace{\left(2 * \frac{40}{60}\right)}_{\text{O atom no.2}} + \underbrace{\left(4 * \frac{8}{60}\right)}_{\text{O atom no.3}}\Big] * (\pi * (1.4)^2) + \underbrace{\left[8 * \frac{1}{2} * \pi * (0.1)^2\right]}_{\text{H atom}} =$$

$47.618 + 0.126 = 47.744\ A^{\circ 2}$

Planar density=$\frac{[Number\ \ of\ atoms\ \ in\ plane\ (230) * area\ \ of\ \ each\ atom\ \ in\ the\ plane\ (230)]}{area\ \ of\ \ the\ plane\ \ (230)} = \frac{47.744}{189.298} = 0.252$

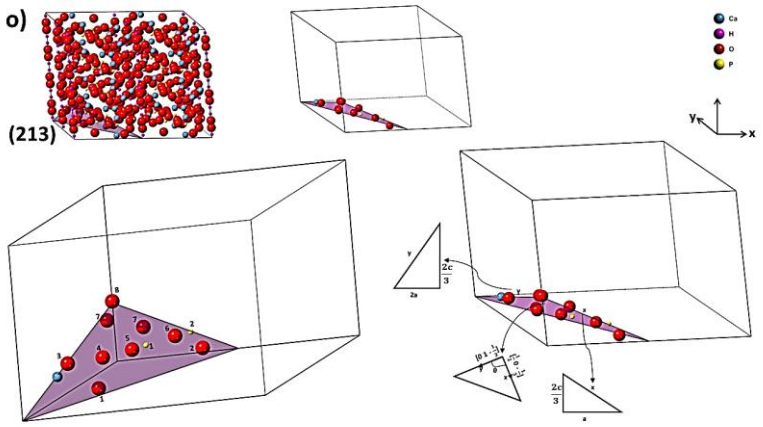

Angle between two miller hexagonal directions:

$[u_1\ v_1\ w_1] < [u_2\ v_2\ w_2]$ $\left[\frac{+1}{2}\ 0\ \frac{-1}{3}\right] \overset{?}{<} \left[0\ 1\ \frac{-1}{3}\right]$

$$cos\theta = \frac{a^2\left[u_1u_2 + v_1v_2 - \frac{1}{2}(u_1v_2 + v_1u_2)\right] + c^2w_1w_2}{\sqrt{a^2(u_1^2 - u_1v_1 + v_1^2) + c^2w_1^2}\ \sqrt{a^2(u_2^2 - u_2v_2 + v_2^2) + c^2w_2^2}}$$

$$= \frac{(9.4)^2\left[0 + 0 - \frac{1}{2}\left(\frac{1}{2}(1) + 0\right)\right] + (6.93)^2 * (\frac{-1}{3})}{\sqrt{(9.4)^2\left(\left(\frac{1}{2}\right)^2 - 0 + 0\right) + (6.93)^2\left(\frac{-1}{3}\right)^2}\ \sqrt{(9.4)^2(0 - 0 + 1^2) + (6.93)^2 * \left(\frac{-1}{3}\right)^2}}$$

$$= \frac{-16.754}{9.679 * 5.237} = -0.33 \rightarrow \theta = 109.3°$$

$$x^2 = (a)^2 + \left(\frac{2c}{3}\right)^2 = (9.4)^2 + \left(\frac{2 * 6.93}{3}\right)^2 = 109.704 \rightarrow x = 10.474$$

$$y^2 = (2a)^2 + \left(\frac{2c}{3}\right)^2 = (18.8)^2 + \left(\frac{2 * 6.93}{3}\right)^2 = 374.784 \rightarrow y = 19.360$$

Area of the plane (213): $S = y * x * 0.5 * \sin(109.3°) = (19.360) * (10.474) * (0.5) * \sin(109.3°) = 95.690\ A^{\circ 2}$

Number of atoms in the plane (213) ×area of each atom in the plane (213)

$$=\underbrace{0.56*\left(\frac{58}{69}\right)*\pi*(1)^2}_{\text{Ca atom}}+\Bigg[\underset{\substack{\text{O atom}\\\text{no.1}}}{\left(\frac{26}{52}*\frac{139}{148}\right)}+\underset{\substack{\text{O atom}\\\text{no.2}}}{\left(\frac{16}{52}\right)}+\underset{\substack{\text{O atom}\\\text{no.3}}}{\left(0.5*\frac{42}{52}\right)}+\underset{\substack{\text{O atom}\\\text{no.4}}}{\left(\frac{8}{52}\right)}+\underset{\substack{\text{O atom}\\\text{no.5}}}{\left(\frac{32}{52}\right)}+\underset{\substack{\text{O atom}\\\text{no.6}}}{\left(\frac{42}{52}\right)}+$$

$$\underset{\substack{\text{O atom}\\\text{no.7}}}{\left(\frac{14}{52}*2\right)}+\underset{\substack{\text{O atom}\\\text{no.8}}}{\left(\frac{35}{52}*\frac{109.3}{360}\right)}\Bigg]*\pi*(1.4)^2+\Bigg[\underset{\substack{\text{P atom}\\\text{no.1}}}{\left(\frac{4}{19}\right)}+\underset{\substack{\text{P atom}\\\text{no.2}}}{\left(\frac{12}{19}\right)}\Bigg]*\pi*(0.31)^2=1.479+$$

$$21.557+0.254=23.29\ \mathring{A}^2$$

Planar density= $\frac{[Number\ \ of\ \ atoms\ \ in\ plane\ (213)*area\ \ of\ \ each\ atom\ \ in\ the\ plane\ (213)]}{area\ \ of\ \ the\ plane\ \ (213)}=$

$\frac{23.29}{95.69}=0.243$

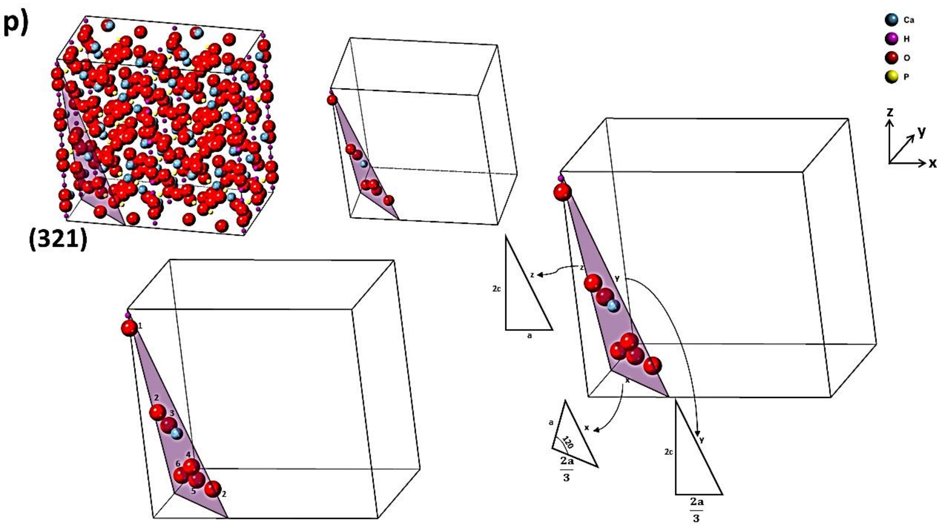

Cosine's rule: $x^2 = \left(\frac{2a}{3}\right)^2 + (a)^2 - 2\left(\frac{2a}{3}\right)(a)cos120^{\circ} = \left(\frac{18.8}{3}\right)^2 + (9.4)^2 - 2\left(\frac{18.8}{3}\right)(9.4)cos120^{\circ} = 186.538 \rightarrow x = 13.658$

$$y^2 = \left(\frac{2a}{3}\right)^2 + (2c)^2 = \left(\frac{18.8}{3}\right)^2 + (2 * 6.93)^2 = 231.371 \rightarrow y = 15.211$$

$$Z^2 = a^2 + (2c)^2 = (9.4)^2 + (2 * 6.93)^2 = 280.460 \rightarrow z = 16.747$$

Area of the plane (321): $s \xrightarrow{Hero\,n's\;law} s = \sqrt{p(p-x)(p-y)(p-z)}$

$$p = \frac{x+y+y}{2} = \frac{(13.658) + (15.211) + (16.747)}{2} \qquad p = 22.808$$

$$s = \sqrt{22.808(22.808 - 13.658)^2(22.808 - 15.211)(22.808 - 16.747)}$$

$$s = 98.027A^{\circ 2}$$

Number of atoms in the plane (321) ×area of each atom in the plane (321)

$$=\Bigg[\underbrace{\left(\frac{22}{53} * \frac{139}{148}\right)}_{\text{O atom no.1}} + \underbrace{\left(\frac{30}{53} * 2\right)}_{\text{O atom no.2}} + \underbrace{\left(\frac{10}{53}\right)}_{\text{O atom no.3}} + \underbrace{\left(\frac{48}{53}\right)}_{\text{O atom no.4}} + \underbrace{\left(\frac{20}{53}\right)}_{\text{O atom no.5}} + \underbrace{\left(\frac{42}{53}\right)}_{\text{O atom no.6}}\Bigg] * \pi * (1.4)^2 +$$

$$\underbrace{\left[\frac{36}{38} * \pi * (1)^2\right]}_{\text{Ca atom}} + \underbrace{\left[\frac{16}{22} * \pi * (0.1)^2\right]}_{\text{H atom}} = 23.313 + 2.976 + 0.031 = 26.320A^{\circ 2}$$

Planar density= $\frac{[Number\ of\ atoms\ in\ plane\,(321) * area\ of\ each\ atom\ in\ the\ plane\ (321)]}{area\ of\ the\ plane\ (321)} = \frac{26.320}{98.027} = 0.269$

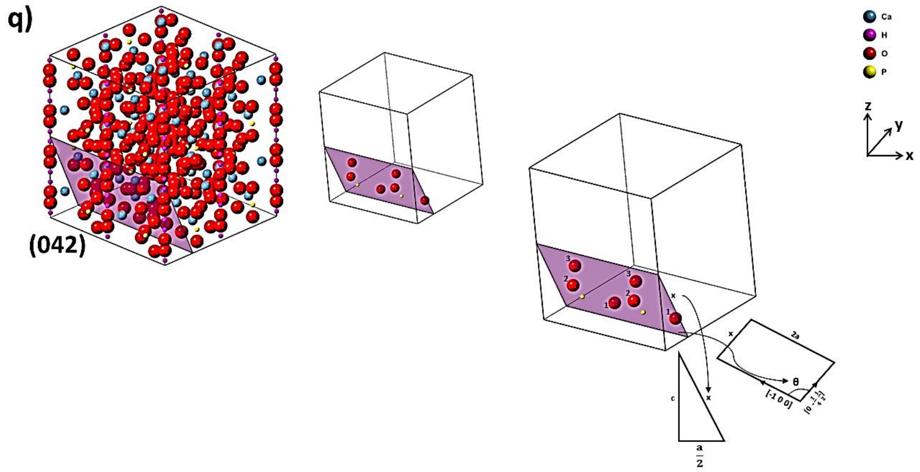

$$x^2 = \left(\frac{a}{2}\right)^2 + (c)^2 = \left(\frac{9.4}{2}\right)^2 + (6.93)^2 = 70.115 \to x = 8.373$$

Angle between two miller hexagonal directions:

$$[u_1\ v_1\ w_1] < [u_2\ v_2\ w_2] \quad [-1\ 0\ 0] \measuredangle \left[0\ \frac{-1}{4}\ \frac{1}{2}\right]$$

$$cos\theta = \frac{a^2\left[u_1u_2 + v_1v_2 - \frac{1}{2}(u_1v_2 + v_1u_2)\right] + c^2w_1w_2}{\sqrt{a^2(u_1^2 - u_1v_1 + v_1^2) + c^2w_1^2}\ \sqrt{a^2(u_2^2 - u_2v_2 + v_2^2) + c^2w_2^2}}$$

$$cos\theta = \frac{(9.4)^2\left[0 + 0 - \frac{1}{2}\left((-1)\left(\frac{-1}{4}\right) + 0\right)\right] + 0}{\sqrt{(9.4)^2((-1)^2 - 0 + 0) + 0}\ \sqrt{(9.4)^2\left(0 - 0 + \left(\frac{-1}{4}\right)^2\right) + (6.93)^2\left(\frac{1}{2}\right)^2}}$$

$$cos\theta = \frac{(9.4)^2\left(\frac{-1}{8}\right)}{\sqrt{(9.4)^2}\sqrt{(9.4)^2\left(\frac{1}{16}\right) + (6.93)^2 * \frac{1}{4}}} = -0.281 \to \theta = 106.299°$$

Area of the plane (032): $S = (2a) * (x) * \sin\theta = (18.8) * (8.373) * \sin(106.299°) = 151.086 A^{°2}$

Number of atoms in the plane (042) ×area of each atom in the plane (042)

$$=\left[\underbrace{\left(2 * \frac{48}{108}\right)}_{\text{O atom no.1}} + \underbrace{\left(2 * \frac{72}{108}\right)}_{\text{O atom no.2}} + \underbrace{\left(2 * \frac{64}{108}\right)}_{\text{O atom no.3}}\right] * \pi * (1.4)^2 + \underbrace{\left[2 * \frac{14}{20} * \pi * (0.31)^2\right]}_{\text{P atom}} =$$

$$20.981 + 0.423 = 21.404\ A^{°2}$$

Planar density$= \frac{[Number\ of\ atoms\ in\ plane\ (042) * area\ of\ each\ atom\ in\ the\ plane\ (042)]}{area\ of\ the\ plane\ (042)} = \frac{21.404}{151.086} = 0.142$

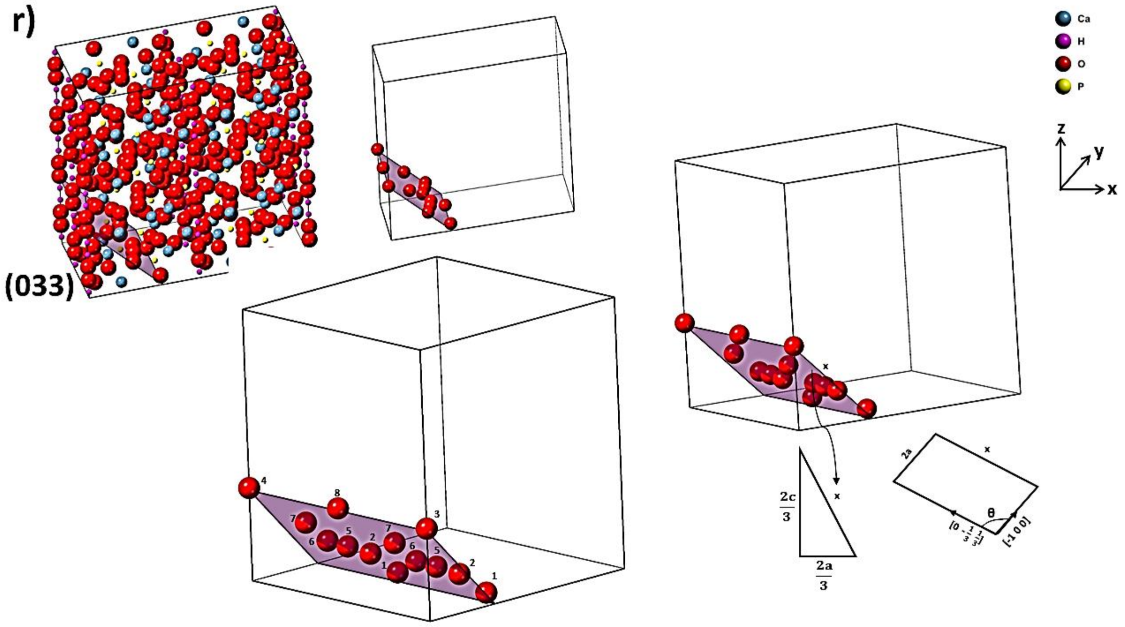

$$x^2 = \left(\frac{2a}{3}\right)^2 + \left(\frac{2c}{3}\right)^2 = \left(\frac{18.8}{3}\right)^2 + \left(\frac{2 * 6.93}{3}\right)^2 = 60.616 \rightarrow x = 7.786$$

Angle between two miller hexagonal directions:

$$[u_1\ v_1\ w_1] < [u_2\ v_2\ w_2] \quad [-1\ 0\ 0] \sphericalangle \left[0\ \frac{-1}{3}\ \frac{1}{3}\right]$$

$$cos\theta = \frac{a^2\left[u_1u_2 + v_1v_2 - \frac{1}{2}(u_1v_2 + v_1u_2)\right] + c^2w_1w_2}{\sqrt{a^2(u_1^2 - u_1v_1 + v_1^2) + c^2w_1^2}\ \sqrt{a^2(u_2^2 - u_2v_2 + v_2^2) + c^2w_2^2}}$$

$$= \frac{(9.4)^2\left[0 + 0 - \frac{1}{2}\left((-1)\left(\frac{-1}{3}\right) + 0\right)\right] + (6.93)^2 * (0)}{\sqrt{(9.4)^2((-1)^2 - 0 + 0) + (6.93)^2(0)^2}\ \sqrt{(9.4)^2\left(0 - 0 + \left(\frac{-1}{3}\right)^2\right) + (6.93)^2 * \left(\frac{1}{3}\right)^2}}$$

$$cos\theta = \frac{(9.4)^2\left(\frac{-1}{6}\right)}{\sqrt{(9.4)^2}\sqrt{(9.4)^2\left(\frac{1}{9}\right) + (6.93)^2\left(\frac{1}{9}\right)}} = -0.402 \rightarrow \theta = 113.732°$$

Area of the plane (033): $S = (2a) * (x) * \theta = (18.8) * (7.786) * \sin(113.732°) = 133.992\ A^{\circ 2}$

Number of atoms in the plane (033) × area of each atom in the plane (033)

$$=\Bigg[\underbrace{\left(\frac{44}{107} * \frac{139}{148} * 2\right)}_{\text{O atom no.1}} + \underbrace{\left(\frac{104}{107} * 2\right)}_{\text{O atom no.2}} + \underbrace{\left(\frac{92}{107} * \frac{113.732}{360}\right)}_{\text{O atom no.3}} + \underbrace{\left(\frac{92}{107} * \frac{66.268}{360}\right)}_{\text{O atom no.4}} + \underbrace{\left(\frac{60}{107} * 2\right)}_{\text{O atom no.5}} +$$

$$\underbrace{\left(\frac{40}{17} * 2\right)}_{\text{O atom no.6}} + \underbrace{\left(\frac{88}{107} * 2\right)}_{\text{O atom no.7}} + \underbrace{\left(\frac{92}{107} * 0.5\right)}_{\text{O atom no.8}}\Bigg] * \pi * (1.4)^2 = 43.657\ A^{\circ 2}$$

Planar density $= \frac{[Number\ of\ atoms\ in\ plane(033) * area\ of\ each\ atom\ in\ the\ plane\ (033)]}{area\ of\ the\ plane\ (033)} = \frac{43.657}{133.992} = 0.326$

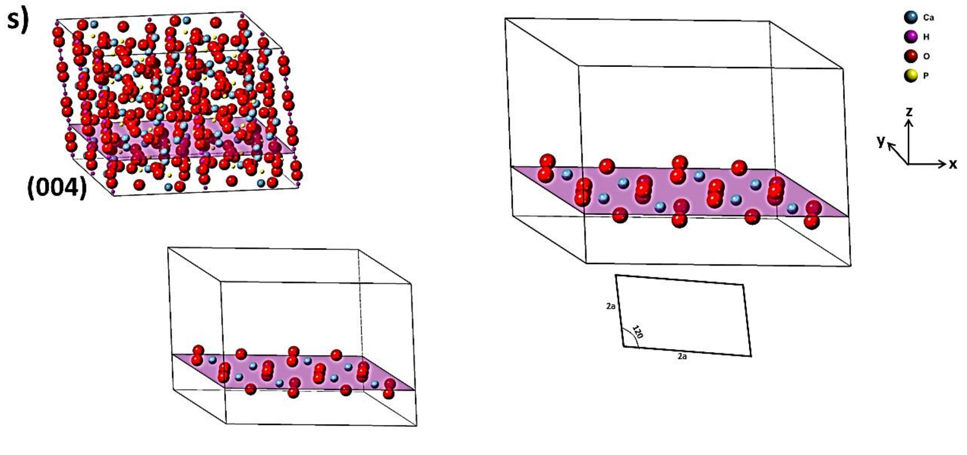

Area of the (004): s= (2a)×(2a)×sin120°= (18.8)×(18.8)×sin120°=306.088 $A^{\circ 2}$

Number of atoms in the plane (004)×area of each atom in the plane (004)

$$=\underbrace{\left(24*\frac{10}{81}\right)*\pi*(1.4)^2}_{\text{O atom}}+\underbrace{(8)*\pi*(1)^2}_{\text{Ca atom}}=18.245+25.133=43.378\ A^{\circ 2}$$

Planar density=$\frac{[Number\ \ of\ atoms\ \ in\ plane\ (004)*area\ \ of\ \ each\ atom\ \ in\ the\ plane\ (004)]}{area\ \ of\ \ the\ plane\ (004)}=$

$\frac{43.378}{306.088}=0.142$

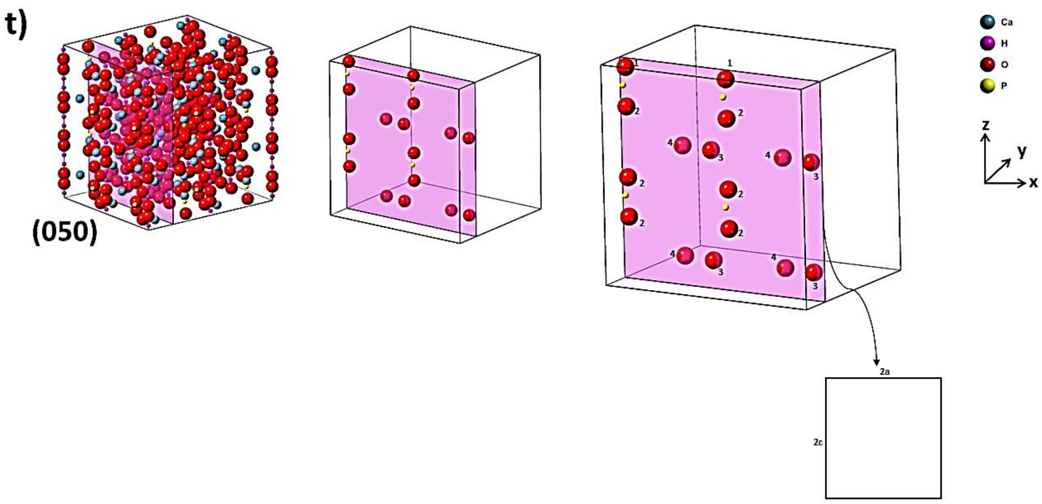

Area of the (050): s= (2a)×(2c) = (18.8)×(2×6.93) =260.568 $A^{\circ 2}$

Number of atoms in the plane (050)×area of each atom in the plane (050)

$$=\left[\underbrace{\left(2*\frac{22}{138}*\frac{139}{148}\right)}_{\text{O atom no.1}}+\underbrace{\left(6*\frac{22}{138}\right)}_{\text{O atom no.2}}+\underbrace{\left(\frac{92}{138}*4\right)}_{\text{O atom no.3}}+\underbrace{\left(\frac{8}{138}*4\right)}_{\text{O atom no.4}}\right]*\pi*(1.4)^2+$$

$$\underbrace{\left[4*\frac{20}{25}*\pi*(0.31)^2\right]}_{\text{P atom}}=25.582+0.966=26.548\ A^{\circ 2}$$

Planar density=$\frac{[Number\ \ of\ atoms\ \ in\ plane\ (050)*area\ \ of\ \ each\ atom\ \ in\ the\ plane\ (050)]}{area\ \ of\ \ the\ plane\ \ (050)}=$

$\frac{26.548}{260.568}=0.102$

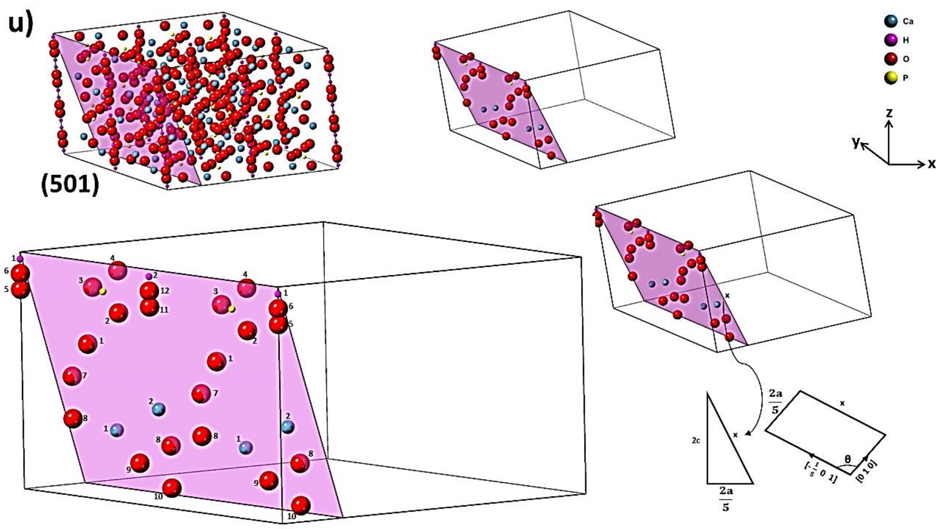

$$x^2 = \left(\frac{2a}{5}\right)^2 + (2c)^2 = \left(\frac{18.8}{5}\right)^2 + (2 * 6.93)^2 = 206.237 \rightarrow x = 14.361$$

Angle between two miller hexagonal directions:

$$[u_1\ v_1\ w_1] < [u_2\ v_2\ w_2] \quad [0\ 1\ 0] \overset{?}{<} \left[\frac{-1}{5}\ 0\ \ 1\right]$$

$$cos\theta = \frac{a^2\left[u_1u_2 + v_1v_2 - \frac{1}{2}(u_1v_2 + v_1u_2)\right] + c^2w_1w_2}{\sqrt{a^2(u_1^2 - u_1v_1 + v_1^2) + c^2w_1^2}\ \sqrt{a^2(u_2^2 - u_2v_2 + v_2^2) + c^2w_2^2}}$$

$$= \frac{(9.4)^2\left[0 + 0 - \frac{1}{2}\left(0 + (1 * \frac{-1}{5})\right)\right] + (6.93)^2 * (0)}{\sqrt{(9.4)^2(0 - 0 + 1^2) + (6.93)^2 * 0}\ \sqrt{(9.4)^2\left(\left(\frac{-1}{5}\right)^2 + 0 + 0\right) + (6.93)^2 * 1^2}}$$

$$= \frac{(9.4)^2\left(\frac{1}{10}\right)}{(9.4)\sqrt{(9.4)^2\left(\frac{1}{25}\right) + (6.93)^2}} = 0.131 \rightarrow \theta = 82.473°$$

Area of the plane (501): $S = (2a) * (x) * \sin\theta = (18.8) * (14.361) * \sin(82.473°) = 267.66\ Å^2$

Number of atoms in the plane (501) ×area of each atom in the plane (501)

$$=\Big[\underbrace{\left(\frac{106}{119} * 2\right)}_{\text{O atom no.1}} + \underbrace{\left(\frac{116}{119} * 2\right)}_{\text{O atom no.2}} + \underbrace{\left(\frac{6}{119} * 2\right)}_{\text{O atom no.3}} + \underbrace{\left(\frac{2}{119} * \frac{139}{148} * 2\right)}_{\text{O atom no.4}} + \underbrace{\left(2 * \frac{16}{119} * 0.5\right)}_{\text{O atom no.5}} +$$

$$\underbrace{\left(2 * \frac{30}{119} * 0.5\right)}_{\text{O atom no.6}} + \underbrace{\left(\frac{48}{119} * 2\right)}_{\text{O atom no.7}} + \underbrace{\left(\frac{72}{119} * 4\right)}_{\text{O atom no.8}} + \underbrace{\left(\frac{80}{119} * 2\right)}_{\text{O atom no.9}} + \underbrace{\left(\frac{48}{119} * \frac{139}{148} * 2\right)}_{\text{O atom no.10}} + \underbrace{\left(\frac{16}{119}\right)}_{\text{O atom no.11}} +$$

$$\underbrace{\left(\frac{30}{119}\right)}_{\text{O atom no.12}}\Big] * \pi * (1.4)^2 + \Big[\underbrace{\left(\frac{16}{43} * 2\right)}_{\text{Ca atom no.1}} + \underbrace{\left(\frac{42}{43} * 2\right)}_{\text{Ca atom no.2}}\Big] * \pi * (1)^2 + \underbrace{\left(\frac{8}{43} * 2 * \pi * (0.31)^2\right)}_{\text{P atom}} +$$

$$\Big[\underbrace{\left(0.5 * \frac{22}{28} * 2\right)}_{\text{H atom no.1}} + \underbrace{\left(\frac{22}{28}\right)}_{\text{H atom no.2}}\Big] * \pi * (0.1)^2 = 61.364 + 8.475 + 0.112 + 0.049 =$$

$70.00\ Å^2$

Planar density$=\frac{[Number\ \ of\ atoms\ \ in\ plane\ (501) * area\ \ of\ \ each\ atom\ \ in\ the\ plane\ (501)]}{area\ \ of\ \ the\ plane\ (501)} = \frac{70}{267.66} = 0.262$

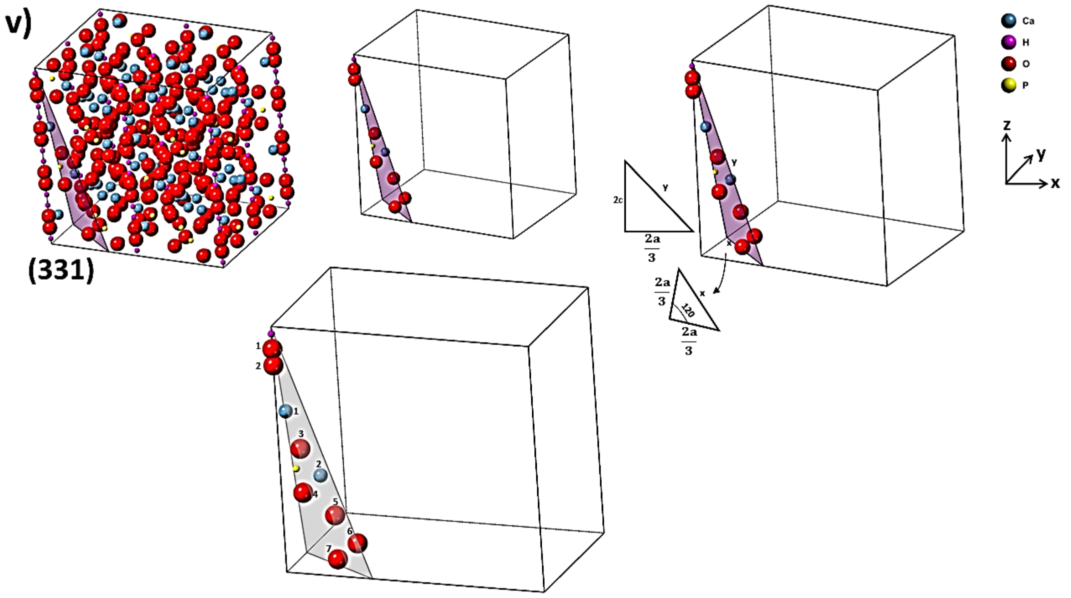

Cosine's law: $x^2 = \left(\frac{2a}{3}\right)^2 + \left(\frac{2a}{3}\right)^2 - 2\left(\frac{2a}{3}\right)\left(\frac{2a}{3}\right) cos120^{\circ} = \left(\frac{18.8}{3}\right)^2 + \left(\frac{18.8}{3}\right)^2 + (\frac{18.8}{3})\left(\frac{18.8}{3}\right) = 117.813 \rightarrow x = 10.854$

$$y^2 = \left(\frac{2a}{3}\right)^2 + (2c)^2 = \left(\frac{18.8}{3}\right)^2 + (2 * 6.93)^2 = 231.371 \rightarrow y = 15.211$$

Area of the plane (331): $s \xrightarrow{Heron's\ law} s = \sqrt{p(p-x)(p-y)(p-y)}$

$$p = \frac{x+y+y}{2} = 20.638 \rightarrow$$

$$s = \sqrt{(20.638)(20.638 - 10.854) * (20.638 - 15.211)^2} = 77.117 A^{\circ 2}$$

Number of atoms in the plane (331) ×area of each atom in the plane (331)

$$= \Big[\underbrace{\left(0.5 * \frac{56}{108}\right)}_{\text{O atom no.1}} + \underbrace{\left(0.5 * \frac{16}{108}\right)}_{\text{O atom no.2}} + \underbrace{\left(\frac{48}{108}\right)}_{\text{O atom no.3}} + \underbrace{\left(\frac{64}{108}\right)}_{\text{O atom no.4}} + \underbrace{\left(\frac{40}{108}\right)}_{\text{O atom no.5}} + \underbrace{\left(\frac{44}{108}\right)}_{\text{O atom no.6}} + \underbrace{(\frac{68}{108} * \frac{139}{148})}_{\text{O atom no.7}}\Big] * \pi *$$

$$(1.4)^2 + \Big[\underbrace{\left(0.56 * \frac{34}{40}\right)}_{\text{Ca atom no.1}} + \underbrace{\left(\frac{8}{40}\right)}_{\text{Ca atom no.2}}\Big] * \pi * (1)^2 + \underbrace{\left(\frac{16}{19}\right) * \pi * (0.31)^2}_{\text{P atom}} + \underbrace{\left(0.5 * \frac{10}{19}\right)}_{\text{H atom}} * \pi *$$

$$(0.1)^2 = 16.868 + 2.124 + 0.254 + 0.0083 = 19.254\ A^{\circ 2}$$

Planar density $= \frac{[Number\ of\ atoms\ in\ plane\ (331) * area\ of\ each\ atom\ in\ the\ plane\ (331)]}{area\ of\ the\ plane\ (331)} =$
$\frac{19.254}{77.117} = 0.250$

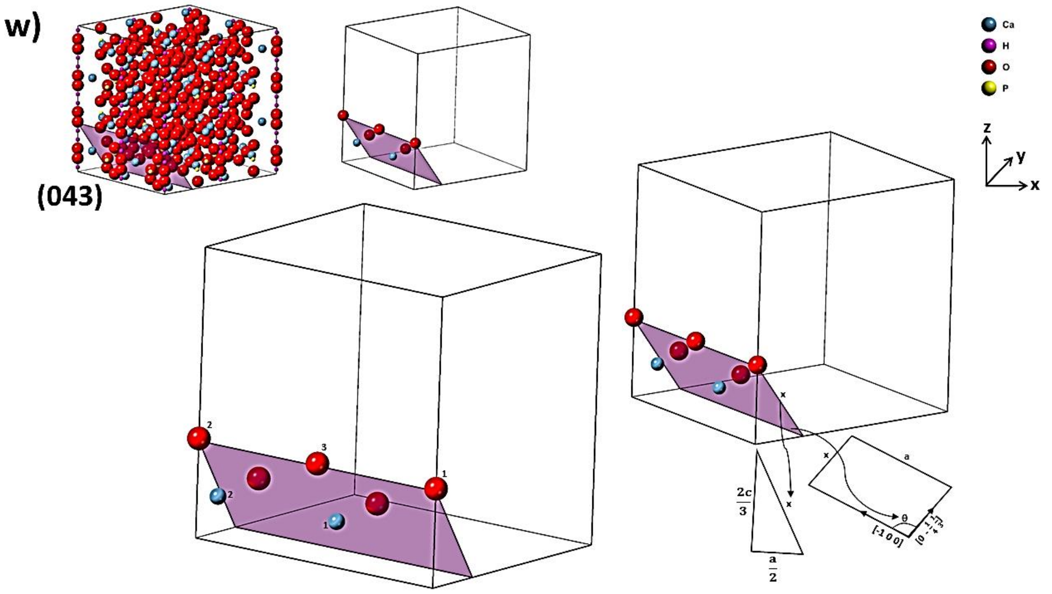

$$x^2 = \left(\frac{a}{2}\right)^2 + \left(\frac{2c}{3}\right)^2 = \left(\frac{9.4}{2}\right)^2 + \left(\frac{2 * 6.93}{3}\right)^2 = 43.434 \rightarrow x = 6.59$$

Angle between two miller hexagonal directions:

$$[u_1\ v_1\ w_1] < [u_2\ v_2\ w_2] \quad [-1\ 0\ 0] \sphericalangle \left[0\ \frac{-1}{4}\ \frac{1}{3}\right]$$

$$cos\theta = \frac{a^2\left[u_1u_2 + v_1v_2 - \frac{1}{2}(u_1v_2 + v_1u_2)\right] + c^2w_1w_2}{\sqrt{a^2(u_1^2 - u_1v_1 + v_1^2) + c^2w_1^2}\sqrt{a^2(u_2^2 - u_2v_2 + v_2^2) + c^2w_2^2}}$$

$$cos\theta = \frac{(9.4)^2\left[0 + 0 - \frac{1}{2}\left((-1)\left(\frac{-1}{4}\right) + 0\right)\right] + (0)}{\sqrt{(9.4)^2((-1)^2 - 0 + 0) + 0}\ \sqrt{(9.4)^2\left(0 - 0 + \left(\frac{-1}{4}\right)^2\right) + (6.93)^2 * \left(\frac{1}{3}\right)^2}}$$

$$cos\theta = \frac{(9.4)^2\left(\frac{-1}{8}\right)}{(9.4)\sqrt{(9.4)^2\left(\frac{1}{16}\right) + (6.93)^2\left(\frac{1}{9}\right)}} = -0.357 \rightarrow \theta = 110.9°$$

Area of the plane (043): $S = (2a) * x * \sin\theta = (18.8) * (6.59) * \sin(110.9°)$

$$s = 115.740\ A^{°2}$$

Number of atoms in the plane (043) × area of each atom in the plane (043)

$$= \left[\left(\frac{92}{106} * 0.5 * \frac{110.9}{360}\right) + \left(\frac{92}{106} * 0.5 * \frac{69.1}{360}\right) + \left(\frac{92}{106} * 0.5\right) + \left(2 * \frac{16}{106}\right)\right] * \pi * (1.4)^2 +$$

O atom no.1   O atom no.2   O atom no.3   O atom no.4

$$\left[\left(\frac{4}{38}\right) + (0.56) * \left(\frac{4}{38}\right)\right] * \pi * (1.4)^2 = 5.867 + 0.516 = 6.383\ A^{°2}$$

Ca atom no.1   Ca atom no.2

Planar density $= \frac{[Number\ of\ atoms\ in\ plane\ (043) * area\ of\ each\ atom\ in\ the\ plane\ (043)]}{area\ of\ the\ plane\ (043)} =$

$$\frac{6.383}{115.740} = 0.055$$

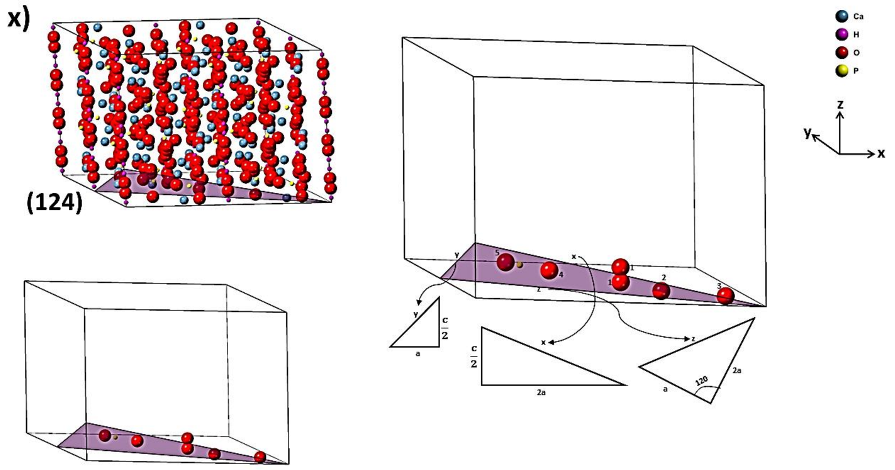

$$x^2 = (\frac{c}{2})^2 + (2a)^2 = \left(\frac{6.93}{2}\right)^2 + (18.8)^2 = 356.446 \rightarrow x = 19.117$$

$$y^2 = \left(\frac{c}{2}\right)^2 + (a)^2 = \left(\frac{6.93}{2}\right)^2 + (9.4)^2 = 100.366 \rightarrow y = 10.018$$

Cosine's rule: $z^2 = (2a)^2 + a^2 - 2(a)(2a)cos120° = (18.8)^2 + (9.4)^2 - 2(9.4)(18.8)cos120° = 618.52 \rightarrow z = 24.870$

Area of the plane (124): $s \xrightarrow{Hero\,n's\ law} s = \sqrt{p(p-x)(p-y)(p-z)}$

$$p = \frac{x+y+y}{2} = \frac{19.117 + 10.018 + 24.870}{2} = 27.003$$

$$s = \sqrt{27.003(27.003 - 19.117) * (27.003 - 10.018) * (27.003 - 24.870)} = 87.833\ A^{\circ 2}$$

Number of atoms in the plane (124)×area of each atom in the plane (124)

$$=\left[\underbrace{\left(2 * \frac{32}{108} * 0.5\right)}_{\text{O atom no.1}} + \underbrace{\left(\frac{68}{108} * \frac{139}{148}\right)}_{\text{O atom no.2}} + \underbrace{\left(\frac{52}{108} * \frac{139}{148}\right)}_{\text{O atom no.3}} + \underbrace{\left(\frac{46}{108}\right)}_{\text{O atom no.4}} + \underbrace{\left(\frac{23}{108}\right)}_{\text{O atom no.5}}\right] * \pi * (1.4)^2 + \left(\frac{4}{44} * \pi * (0.31)^2\right) = 12.184 + 0.027 = 12.211\ A^{\circ 2}$$

Planar density $= \frac{[Number\ of\ atoms\ in\ plane\ (124) * area\ of\ each\ atom\ in\ the\ plane\ (124)]}{area\ of\ the\ plane\ (124)} = \frac{12.211}{87.833} = 0.139$

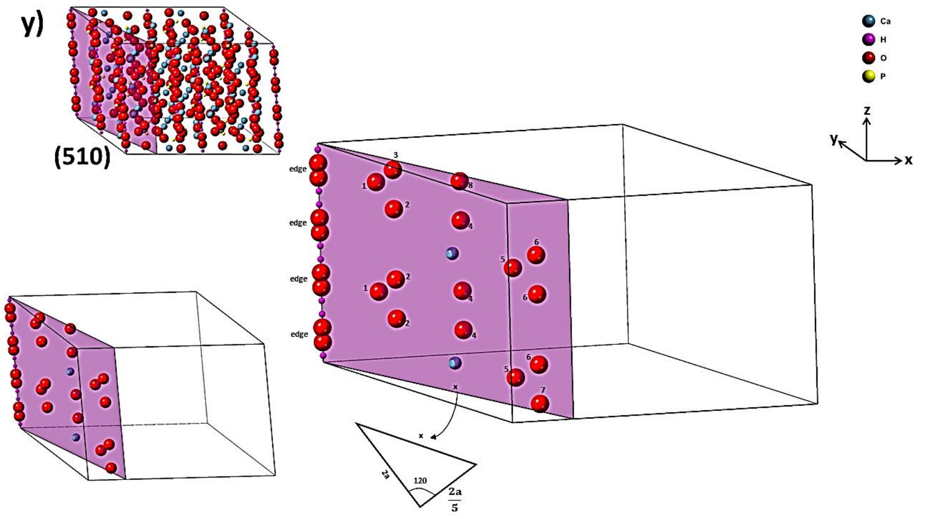

Cosine's rule: $x^2 = \left(\frac{2a}{5}\right)^2 + (2a)^2 - 2\left(\frac{2a}{5}\right)(2a)cos120^{\circ} = \left(\frac{18.8}{5}\right)^2 + (18.8)^2 - 2\left(\frac{18.8}{5}\right) * (18.8) * (-0.5) = 438.266 \rightarrow x = 20.935$

Area of the plane (510): $s = (2c) * (x) = (2 * 6.93) * 20.935 = 290.159\ A^{\circ 2}$

Number of atoms in the plane (510) ×area of each atom in the plane (510)

$$= \Big[\underbrace{(8 * 0.5)}_{\text{O atom edge}} + \underbrace{\left(\frac{76}{128} * 2\right)}_{\text{O atom no.1}} + \underbrace{\left(\frac{52}{128} * 3\right)}_{\text{O atom no.2}} + \underbrace{\left(\frac{52}{128} * \frac{139}{148}\right)}_{\text{O atom no.3}} + \underbrace{\left(\frac{60}{128} * 3\right)}_{\text{O atom no.4}} + \underbrace{\left(\frac{88}{128} * 2\right)}_{\text{O atom no.5}} + \underbrace{\left(\frac{120}{128} * 3\right)}_{\text{O atom no.6}} + \underbrace{\left(\frac{120}{128} * \frac{139}{148}\right)}_{\text{O atom no.7}} + \underbrace{\left(\frac{60}{128} * \frac{139}{148}\right)}_{\text{O atom no.8}}\Big] * \pi * (1.4)^2 + \underbrace{\left(2 * \frac{12}{46} * \pi * (1)^2\right)}_{\text{Ca atom}} + \underbrace{(8 * 0.5 * \pi * (0.1)^2)}_{\text{H atom}} = 84.372 + 1.639 + 0.126 = 86.137\ A^{\circ 2}$$

Planar density $= \frac{[Number\ of\ atoms\ in\ plane\ (510) * area\ of\ each\ atom\ in\ the\ plane\ (510)]}{area\ of\ the\ plane\ (510)} = \frac{86.137}{290.159} = 0.297$

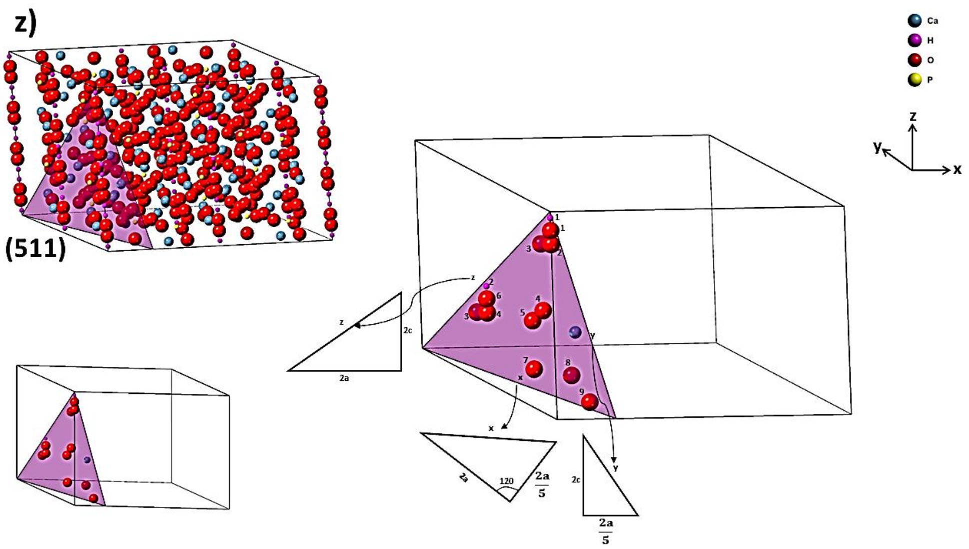

Cosine's rule: $x^2 = \left(\frac{2a}{5}\right)^2 + (2a)^2 - 2\left(\frac{2a}{5}\right)(2a)cos120^{\circ} = \left(\frac{18.8}{5}\right)^2 + (18.8)^2 - 2 * \left(\frac{18.8}{5}\right) * (18.8) * cos120^{\circ} = 438.266 \rightarrow x = 20.935$

$$y^2 = \left(\frac{2a}{5}\right)^2 + (2c)^2 = \left(\frac{18.8}{5}\right)^2 + (2 * 6.93)^2 = 206.237 \rightarrow y = 14.361$$

$$Z^2 = (2a)^2 + (2c)^2 = (18.8)^2 + (2 * 6.93)^2 = 545.540 \rightarrow z = 23.357$$

Area of the plane (511): $s \xrightarrow{Hero\,n'\,s\ law} s = \sqrt{p(p-x)(p-y)(p-z)}$

$$p = \frac{x+y+y}{2} = \frac{20.935 + 14.361 + 23.357}{2} = 29.327$$

$$s = \sqrt{29.327(29.327 - 20.935) * (29.327 - 14.361) * (29.327 - 23.357)} = 148.288A^{\circ 2}$$

Number of atoms in the plane (511) ×area of each atom in the plane (511)

$$=\left[\underbrace{\left(\frac{60}{114} * 0.5\right)}_{\text{O atom no.1}} + \underbrace{\left(\frac{24}{114} * 0.5\right)}_{\text{O atom no.2}} + \underbrace{\left(\frac{20}{114} * 2\right)}_{\text{O atom no.3}} + \underbrace{\left(\frac{24}{114} * 2\right)}_{\text{O atom no.4}} + \underbrace{\left(\frac{88}{114}\right)}_{\text{O atom no.5}} + \underbrace{\left(\frac{60}{114}\right)}_{\text{O atom no.6}} + \underbrace{\left(\frac{32}{114}\right)}_{\text{O atom no.7}} + \underbrace{\left(\frac{8}{114}\right)}_{\text{O atom no.8}} + \underbrace{\left(\frac{104}{114} * \frac{139}{148}\right)}_{\text{O atom no.9}}\right] * \pi * (1.4)^2 + \underbrace{\left[\left(\frac{4}{41}\right) * \pi * (1)^2\right]}_{\text{Ca atom}} + \left[\underbrace{\left(\frac{24}{42} * 0.5\right)}_{\text{H atom no.1}} + \underbrace{\left(\frac{24}{42} * 1\right)}_{\text{H atom no.2}}\right] * \pi * (0.1)^2 = 22.452 + 0.306 + 0.857 = 23.615\ A^{\circ 2}$$

$$\text{Planar density} = \frac{[Number\ \ of\ \ atoms\ \ in\ plane\ (511) * area\ \ of\ \ each\ atom\ \ in\ the\ plane\ (511)]}{area\ \ of\ \ the\ plane\ \ (511)} = \frac{23.615}{148.288} = 0.159$$

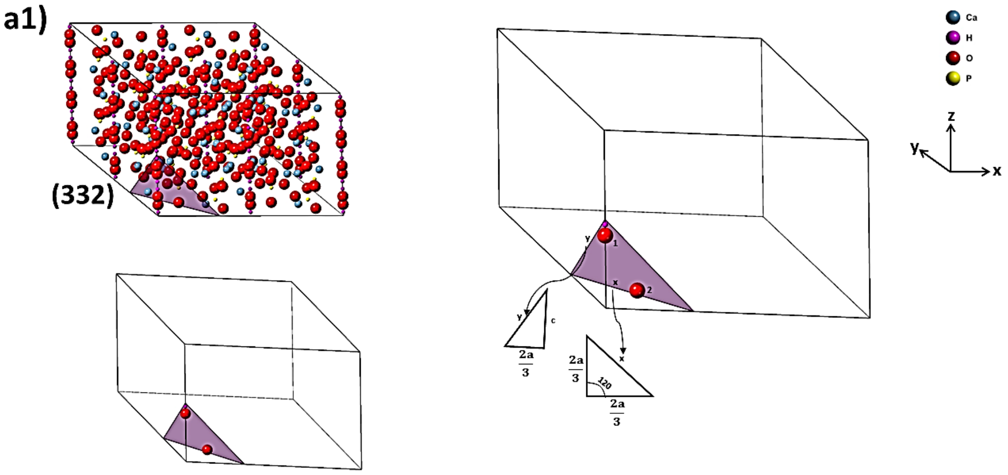

Cosine's rule: $x^2 = \left(\frac{2a}{3}\right)^2 + \left(\frac{2a}{3}\right)^2 - 2\left(\frac{2a}{3}\right)\left(\frac{2a}{3}\right) cos120^{\circ} = \left(\frac{18.8}{3}\right)^2 + \left(\frac{18.8}{3}\right)^2 - 2\left(\frac{18.8}{3}\right)\left(\frac{18.8}{3}\right) * (-0.5) = 117.813 \rightarrow x = 10.854$

$$y^2 = \left(\frac{2a}{3}\right)^2 + (c)^2 = \left(\frac{18.8}{3}\right)^2 + (6.93)^2 = 87.296 \rightarrow y = 9.343$$

Area of the plane (332):$s \xrightarrow{Hero\,n^{'}s\ law} s = \sqrt{p(p-x)(p-y)(p-y)}$

$$p = \frac{x+y+y}{2} = \frac{10.854 + 9.343 + 9.343}{2} = 14.77 \rightarrow$$

$$s = \sqrt{14.77(14.77 - 10.854) * (14.77 - 9.343) * (14.77 - 9.343)} = 41.274\ A^{\circ 2}$$

Number of atoms in the plane (332) ×area of each atom in the plane (332)

$$= \left[\underbrace{\left(\frac{8}{100} * 0.5\right)}_{\text{O atom no.1}} + \underbrace{\left(\frac{84}{100} * \frac{139}{148}\right)}_{\text{O atom no.2}}\right] * \pi * (1.4)^2 + \underbrace{\left[\frac{4}{19} * 0.5 * \pi * (0.1)^2\right]}_{\text{H atom}} = 5.104 + 0.003 = 5.107\ A^{\circ 2}$$

Planar density=$\frac{[Number\ of\ atoms\ in\ plane\ (332) * area\ of\ each\ atom\ in\ the\ plane\ (332)]}{area\ of\ the\ plane\ (332)} = \frac{5.107}{41.274} = 0.124$

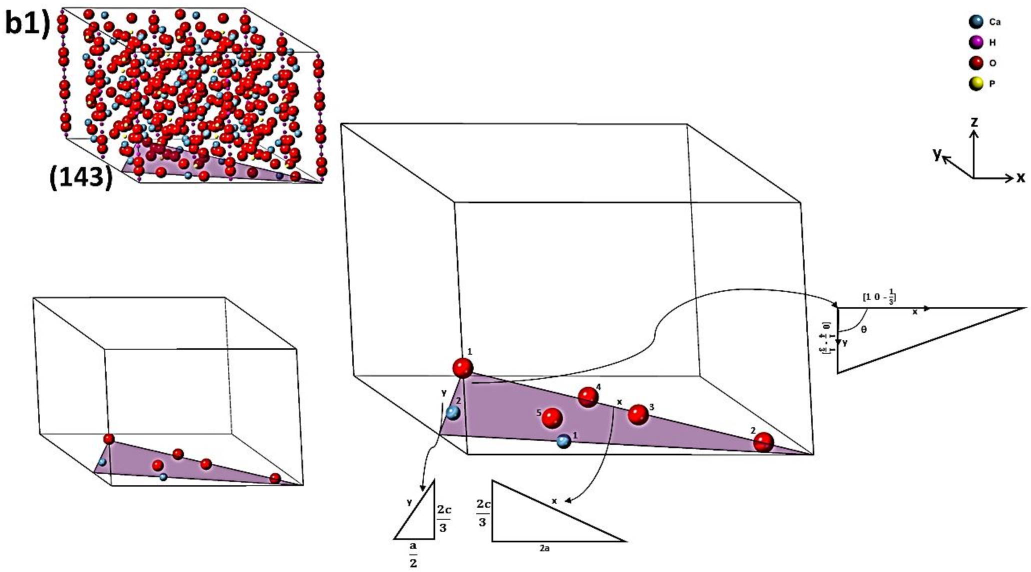

$$x^2 = \left(\frac{2c}{3}\right)^2 + (2a)^2 = \left(\frac{2*6.93}{3}\right)^2 + (18.8)^2 = 374.784 \rightarrow x = 19.359$$

$$y^2 = \left(\frac{2c}{3}\right)^2 + \left(\frac{a}{2}\right)^2 = \left(\frac{2*6.93}{3}\right)^2 + \left(\frac{9.4}{2}\right)^2 = 43.343 \rightarrow y = 6.590$$

Angle between two miller hexagonal directions:

$$[u_1\ v_1\ w_1] < [u_2\ v_2\ w_2] \quad \left[1\ 0\ \frac{-1}{3}\right] < \left[0\ \frac{1}{4}\ \frac{-1}{3}\right]$$

$$cos\theta = \frac{a^2\left[u_1u_2 + v_1v_2 - \frac{1}{2}(u_1v_2 + v_1u_2)\right] + c^2w_1w_2}{\sqrt{a^2(u_1^2 - u_1v_1 + v_1^2) + c^2w_1^2}\sqrt{a^2(u_2^2 - u_2v_2 + v_2^2) + c^2w_2^2}}$$

$$= \frac{(9.4)^2\left[0 + 0 - \frac{1}{2}\left((1)(\frac{1}{4}) + 0\right)\right] + (6.93)^2(\frac{-1}{3})(\frac{-1}{3})}{\sqrt{(9.4)^2(1 - 0 + 0) + (6.93)^2\left(\frac{-1}{3}\right)^2}\sqrt{(9.4)^2\left(0 - 0 + \left(\frac{-1}{4}\right)^2\right) + (6.93) * \left(\frac{-1}{3}\right)^2}}$$

$$= \frac{(9.4)^2 * \left(\frac{-1}{8}\right) + \frac{(6.93)^2}{9}}{\sqrt{(9.4)^2 + (6.93)^2 * (\frac{1}{9})}\sqrt{(9.4)^2\left(\frac{1}{16}\right) + (6.93)^2(\frac{1}{9})}} = \frac{-5.71}{(9.68) * (3.295)} = -0.179$$

$$\rightarrow \theta = 100.313°$$

Area of the plane (143): $S = 0.5 * (x) * (y) * \sin(100.313°) = 0.5 * (19.559) * (6.590) * \sin(100.313) = 62.757\ A^{°2}$

Number of atoms in the plane (143) ×area of each atom in the plane (143)

$$=\left[\left(\frac{88}{102} * \frac{100.313}{360}\right) + \left(\frac{28}{102} * \frac{139}{148}\right) + \left(\frac{88}{102} * 0.5\right) + \left(\frac{12}{102}\right) + \left(\frac{40}{102}\right)\right] * \pi * (1.4)^2 +$$

O atom no.1 | O atom no.2 | O atom no.3 | O atom no.4 | O atom no.5

$$\left[(0.5 * 1) * \left(\frac{4}{18} * 0.57\right)\right] * \pi * (1)^2 = 8.863 + 1.969 = 10.832\ A^{°2}$$

Ca atom no.1 | Ca atom no.2

Planar density= $\frac{[Number\ of\ atoms\ in\ plane\ (143) * area\ of\ each\ atom\ in\ the\ plane\ (143)]}{area\ of\ the\ plane\ (143)} = \frac{10.832}{62.757} = 0.173$

Figure 7. Array and position of the involved atoms such as (**a**) 210, (**b**) 211, (**c**) 112, (**d**) 300, (**e**) 202, (**f**) (301), (**g**) (130), (**h**) (310), (**i**) (032), (**j**) (040), (**k**) (023), (**l**) (222), (**m**) (320), (**n**) (230), (**o**) (213), (**p**) (321), (**q**) (042), (**r**) (033), (**s**) (004), (**t**) (050), (**u**) (501), (**v**) (331), (**w**) (043), (**x**) (124), (**y**) (510), (**z**) (511), (**a1**) (332) and (**b1**) (143) for super-cell (2×2×2) of hydroxyapatite.

Table S3. Young's modulus values of hydroxyapatite related to the literatures and present study.

(hkl)	Young Modulus (E), (Gpa) Extracted by (hkl)				
	Theory. (Ching et al)	Theory. (Leeuw et al)	Experiment. (Katz et al)	Theory. This Study	Experiment. This Study
020	116.18447	103.93059	114.25529	112.59849	109.57044
111	120.48590	121.24439	107.30784	123.31862	108.39178
002	137.53147	133.50251	138.41771	141.12597	144.56056
102	132.54964	135.23285	123.61714	138.17877	129.28471
210	116.18447	103.93059	114.25529	112.59849	109.57044
211	118.21117	113.33961	109.06593	118.28854	107.87865
112	127.20060	133.36361	112.73424	133.33722	117.15015
300	116.18447	103.93059	114.25529	112.59849	109.57044
202	125.61747	131.70198	110.50149	131.45553	114.37075
301	117.78437	111.56711	109.78139	117.20329	108.01888
130	116.18447	103.93059	114.25529	112.59849	109.57044
310	116.18447	103.93059	114.25529	112.59849	109.57044
032	121.60703	124.30894	107.34352	125.41965	109.22865

040	116.18447	103.93059	114.25529	112.59849	109.57044
023	129.97919	134.95579	117.76339	136.11104	122.93008
222	120.48590	121.24439	107.30784	123.31862	108.39178
320	116.18447	103.93059	114.25529	112.59849	109.57044
230	116.18447	103.93059	114.25529	112.59849	109.57044
213	127.00021	133.18648	112.42518	133.11216	116.77725
321	116.96204	107.85168	111.66391	114.95348	108.58244
042	119.5493	118.28301	107.68201	121.38559	107.95993
033	125.61747	131.70198	110.50149	131.45553	114.37075
004	137.53147	133.50251	138.41771	141.12597	144.56056
050	116.18447	103.93059	114.25529	112.59849	109.57044
501	116.77849	106.96407	112.19366	114.41932	108.77045
331	116.73515	106.75115	112.32552	114.29131	108.81863
043	122.65824	126.75128	107.76429	127.20659	110.27998
124	130.05772	134.97962	117.92523	136.18079	123.10982
510	116.18447	103.93059	114.25529	112.59849	109.57044
511	116.665	106.40389	112.54463	114.08263	108.89975
332	118.28095	113.61989	108.96335	118.46111	107.86429
143	121.45516	123.92263	107.31185	125.14745	109.09707

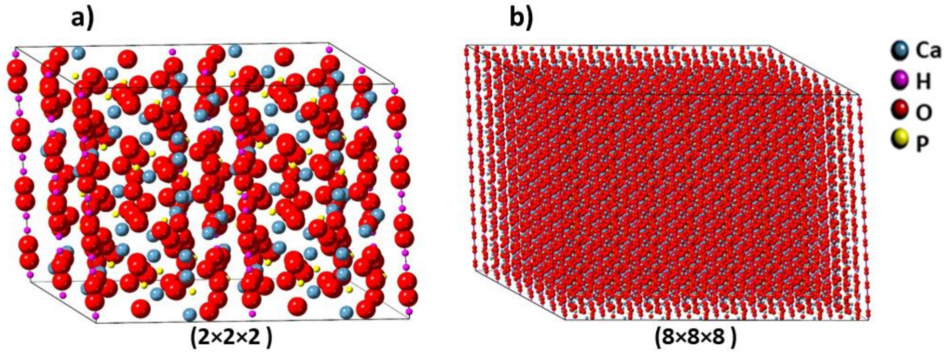

Figure S8. (**a**) The un-symmetry of two unit cells and (**b**) symmetry of eight unit cells of hydroxyapatite.

7. Uncertainty in Measuring Elastic Modulus

To measure the Young's modulus by ultrasonic, we need velosity values to obtain longitudinal and transverse ultrasound waves. Error model is an algebraic expression that quantifies the total error of the result based on combination all the errors of the measurement variables. To form the error model, the equation used to measure the Young's modulus must be derived with respect to all parameters with summation over all those together [11].

$$E = f(L, t_s, t_l) \tag{1}$$

$$\varepsilon_E = \frac{\partial E}{\partial L}\varepsilon_L + \frac{\partial E}{\partial t_s}\varepsilon_{t_s} + \frac{\partial E}{\partial t_l}\varepsilon_{t_l} = a_L\varepsilon_L + a_{t_s}\varepsilon_{t_s} + a_{t_l}\varepsilon_{t_l} \tag{2}$$

The above Equations (1) and (2) mean that the measurement of errors in L, t_s and t_l leads to measurement error in E. In Equation (3), constant density is considered and therefore a derivative with respect to the density is zero. The density value is obtained 2937.87 Kg/m^3. If n factors creat a measurement error, the uncertainty of the result R is obtained according to the Equation (3) [12]:

$$u_R = t_{\vartheta,95} = \left[\sum_{1}^{n}\left(a_{x_i}u_{x_i}\right)^2\right]^{\frac{1}{2}} \tag{3}$$

In Equation (3), u_{xi} is uncertainty of x_i and a_{xi} is sensitivity coefficient of x_i. The degree of participation of x_i in uncertainty of R property is estimated by $a_{xi}u_{xi}$. $t_{v,95}$ and ν are the distribution coefficient and the degree of freedom, respectively and they are determined based on the number of repetitions of the measurements. Equation (3) is utilized to determine the uncertainty in measuring the Young's modulus. This equation by considering the sources of error in measuring the Young's modulus will be as follows (Equation (4)):

$$u_E = t_{\vartheta,95}\ \left[(a_L u_L)^2 + (a_{t_s} u_{t_s})^2 + (a_{t_l} u_{t_l})^2\right]^{\frac{1}{2}} \quad (4)$$

t_l and t_s, L are measured independently with respect to each other, therefore there is no correlation between errors. Identify the errors of a measurement to estimate the uncertainty is essential. Measuring Young's modulus by ultrasonic method involves errors causes by measured t_l and t_s, L as stated as follows:

$$\varepsilon_L = \varepsilon_{L,ran} + \varepsilon_{L,sys} \quad (5)$$

$$\varepsilon_{t_s} = \varepsilon_{t_s,ran} + \varepsilon_{t_s,sys} \quad (6)$$

$$\varepsilon_{t_l} = \varepsilon_{t_l,ran} + \varepsilon_{t_l,sys} \quad (7)$$

In Equations (5) to (7) the sys subtitle indicates a systematic error and the ran subtitle indicates a random error. The accuracy of length and time measuring instruments is equal to 0.01.

Uncertainty of measurement, such as measurement errors are divided into two categories: systematic and random. Therefore, each source of error has two uncertainties:

$U_{L,sys}$, $U_{ts,sys}$ and $U_{tl,sys}$ are systematic uncertainties. These uncertainties are due to the accuracy of the device and by the manufacturer of the device is announced. Systematic uncertainties are considered equal to half the accuracy of the device [7], so we will have:

$$u_{t_{s,sys}} = u_{t_{l,sys}} = 0.005\ \mu s \quad (8)$$

$$u_{L,sys} = 0.005\ mm \quad (9)$$

$u_{L,ran}$, $u_{ts,ran}$ and $u_{tl,ran}$ are random uncertainties. This type of uncertainty are determined by statistical calculations of measurement data of Table S4. Because of number of repetitions are less than 30, so for statistical calculations, finite-sized statistics and Student's t-distribution are used. Hence the random uncertainties are calculated by following equations:

In Equations (10) to (12), S_i are standard deviation of mean values of i source and is calculated by Equation (13).

$$u_{L,ran} = S_{\bar{L}} \quad (10)$$

$$u_{t_{s,ran}} = S_{\bar{t_s}} \quad (11)$$

$$u_{t_{l,ran}} = S_{\bar{t_l}} \quad (12)$$

$$S_{\bar{i}} = \frac{S_i}{\sqrt{5}} \quad (13)$$

In Equation (13), S_i are deviation of 5 measurement repetitions for each source of error. Consequently, random uncertainties are as follows:

$$u_{L,ran} = 0.01\ mm \quad (14)$$

$$u_{t_{s,ran}} = 0.005\ \mu s \quad (15)$$

$$u_{t_{l,ran}} = 0.007\ \mu s \quad (16)$$

To calculate the uncertainty of an error source, the summation of the roots square of the systematic and random uncertainties of that source of the error is used [7]:

$$u_L = \sqrt{u_{L,sys}^2 + u_{L,ran}^2} = 0.011 \text{ mm} \quad (17)$$

$$u_{t_s} = \sqrt{u_{t_s,sys}^2 + u_{t_s,ran}^2} = 0.0071 \mu s \quad (18)$$

$$u_{t_l} = \sqrt{u_{t_l,sys}^2 + u_{t_l,ran}^2} = 0.0087 \ \mu s \quad (19)$$

Sensitivity coefficients for these error sources in Equation (2), are also calculated by:

$$a_L = \left(\frac{\partial E}{\partial L}\right)_{(L,t_s,t_l)=(\overline{L},\overline{t_s},\overline{t_l})} = -3.25 \times 10^9 \text{ Pa/mm} \quad (20)$$

$$a_{t_s} = \left(\frac{\partial E}{\partial t_s}\right)_{(L,t_s,t_l)=(\overline{L},\overline{t_s},\overline{t_l})} = -3.0 \times 10^{15} \text{ Pa/s} \quad (21)$$

$$a_{t_l} = \left(\frac{\partial E}{\partial t_l}\right)_{(L,t_s,t_l)=(\overline{L},\overline{t_s},\overline{t_l})} = 3.0 \times 10^{15} \text{ Pa/s} \quad (22)$$

Finally, given that the number of repetitions of the measurements is equal to 5, so the degree of freedom υ is equal to 4 and the distribution coefficient is equal to $t_{4.95} = 2.77$.

Uncertainties calculated in Equations (17) to (19) and the sensitivity coefficients calculated in Equations (20) to (22) are replaced in Equation (4), and the uncertainty of the Young's modulus measurement is obtained by ultrasonic method. Therefore, the values of the Young's modulus and the uncertainty of its measurement by ultrasound is as follows:

$$u_E = t_{4,95}\left[(a_l u_l)^2 + (a_{t_s} u_{t_s})^2 + (a_{t_l} u_{t_l})^2\right]^{\frac{1}{2}} = 0.14 \text{ GPa } (95\%)$$

$$E = 113.08 \pm 0.14 \text{ GPa } (95\%) \quad (23)$$

References

1. Lee, S.; Chun, L.Y.; Soo, Y.Y. Characteristics of calcium phosphate powders synthesized from cuttlefish bone and phosphoric acid. *J. Ceram. Process. Res.* **2007**, *8*, 427–430.
2. Characterization of Hydroxyapatite derived from Bovine Bone | Asian Journal of Applied Sciences Available online: https://www.ajouronline.com/index.php/AJAS/article/view/2777 (accessed on 19 November 2020).
3. Herliansyah, M.K.; Nasution, D.A.; Bin Abdul Shukor, M.H.; Ide-Ektessabi, A.; Wildan, M.W.; Tontowi, A.E. Preparation and Characterization of Natural Hydroxyapatite: A Comparative Study of Bovine Bone Hydroxyapatite and Hydroxyapatite from Calcite. *Mater. Sci. Forum* **2007**, *561–565*, 1441–1444, doi:10.4028/www.scientific.net/msf.561-565.1441.
4. Salleh, E.M.; Ramakrishnan, S.; Hussain, Z. Synthesis of Biodegradable Mg-Zn Alloy by Mechanical Alloying: Effect of Milling Time. *Procedia Chem.* **2016**, *19*, 525–530, doi:10.1016/j.proche.2016.03.048.
5. Cullity, B. *Elements of X-ray diffraction*; Reading, Mass.: Addison-Wesley Pub. Co., c1956; Boston, USA.
6. Preston, G.D. Elements of X-ray Diffraction by B. D. Cullity . *Acta Crystallogr.* **1957**, *10*, 389–389, doi:10.1107/s0365110x57001255.
7. Wilson, R.M.; Elliott, J.C.; Dowker, S.E.P. Rietveld refinement of the crystallographic structure of human dental enamel apatites. *Am. Mineral.* **1999**, *84*, 1406–1414, doi:10.2138/am-1999-0919.
8. Yuan, Z.; Li, S.; Liu, J.; Kong, X.; Gao, T. Structural, electronic, dynamical and thermodynamic properties of Ca10(PO4)6(OH)2 and Sr10(PO4)6(OH)2: First-principles study. *Int. J. Hydrogen Energy* **2018**, *43*, 13639–13648, doi:10.1016/j.ijhydene.2018.03.096.
9. Bhat, S.S.; Waghmare, U. V.; Ramamurty, U. First-principles study of structure, vibrational, and elastic properties of stoichiometric and calcium-deficient hydroxyapatite. *Cryst. Growth Des.* **2014**, *14*, 3131–3141, doi:10.1021/cg5004269.
10. Moradi, K.; Sabbagh Alvani, A.A. First-principles study on Sr-doped hydroxyapatite as a biocompatible filler for photo-cured dental composites. *J. Aust. Ceram. Soc.* **2020**, *56*, 591–598, doi:10.1007/s41779-019-00369-9.
11. NASA-HDBK-8739.19-3 | NASA Technical Standards System (NTSS) Available online: https://standards.nasa.gov/standard/osma/nasa-hdbk-873919-3 (accessed on 24 November 2020).
12. Theory and Design for Mechanical Measurements, 7th Edition | Wiley Available online: https://www.wiley.com/en-us/Theory and+Design+for+Mechanical+Measurements%2C+7th+Edition-p-9781119475651 (accessed on 19 November 2020).
